# Atomically precise surface chemistry of zirconium and hafnium metal oxo clusters beyond carboxylate ligands†

**DOI:** 10.1039/d4sc03859b

**Published:** 2024-10-07

**Authors:** Ajmal Roshan Unniram Parambil, Rohan Pokratath, Muhammed Jibin Parammal, Evert Dhaene, Dietger Van den Eynden, Sandor Balog, Alessandro Prescimone, Ivan Infante, Patrick Shahgaldian, Jonathan De Roo

**Affiliations:** a Institute of Chemistry and Bioanalytics, School of Life Sciences, University of Applied Sciences and Arts Northwestern Switzerland 4132 Muttenz Switzerland; b Department of Chemistry, University of Basel Mattenstrasse 22 4058 Basel Switzerland jonathan.deroo@unibas.ch; c Swiss Nanoscience Institute Klingelbergstrasse 82 4056 Basel Switzerland; d Department of Chemistry, University of Ghent Krijgslaan 281 9000 Ghent Belgium; e Adolphe Merkle Institute, University of Fribourg 1700 Fribourg Switzerland; f BCMaterials Spain

## Abstract

The effectiveness of nanocrystals in many applications depends on their surface chemistry. Here, we leverage the atomically precise nature of zirconium and hafnium oxo clusters to gain fundamental insight into the thermodynamics of ligand binding. Through a combination of theoretical calculations and experimental spectroscopic techniques, we determine the interaction between the M_6_O_8_^8+^ (M = Zr, Hf) cluster surface and various ligands: carboxylates, phosphonates, dialkylphosphinates, and monosubstituted phosphinates. We refute the common assumption that the adsorption energy of an adsorbate remains unaffected by the surrounding adsorbates. For example, dialkylphosphinic acids are too sterically hindered to yield complete ligand exchange, even though a single dialkylphosphinate has a high binding affinity. Monoalkyl or monoaryl phosphinic acids do replace carboxylates quantitatively and we obtained the crystal structure of M_6_O_8_H_4_(O_2_P(H)Ph)_12_ (M = Zr, Hf), giving insight into the binding mode of monosubstituted phosphinates. Phosphonic acids cause a partial structural reorganization of the metal oxo cluster into amorphous metal phosphonate as indicated by pair distribution function analysis. These results rationalize the absence of phosphonate-capped M_6_O_8_ clusters and the challenge in preparing Zr phosphonate metal–organic frameworks. We thus further reinforce the notion that monoalkylphosphinates are carboxylate mimics with superior binding affinity.

## Abstract 2

Computational and experimental investigation of metal oxo cluster surface chemistry *via* ligand exchange reactions with phosphinic and phosphonic acid ligands.

## Graphical abstract

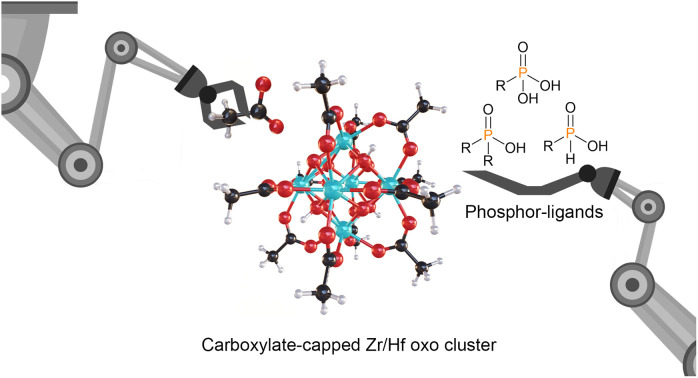

## Introduction

**Scheme 1:**
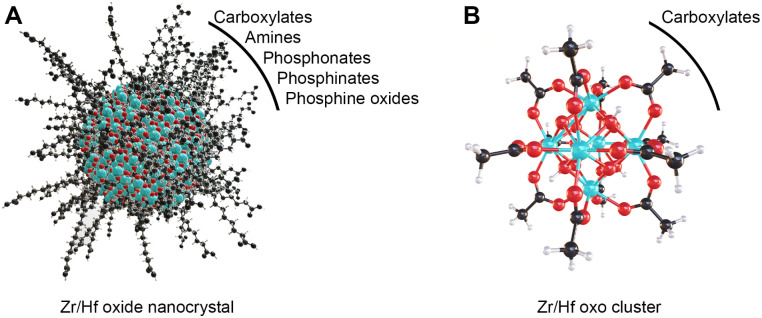
(A) Schematic representation of a colloidally stable zirconium/hafnium oxide nanocrystal depicting the inorganic core and organic ligand shell. (B) The structure of M_6_O_8_H_4_(OOCR)_12_, where the M_6_O_8_ core is capped with four protons and twelve carboxylate ligands, M = Zr/Hf. The ligands that have been reported to cap nanocrystals and clusters in nonpolar media are listed. Cyan atoms represent zirconium or hafnium, all other atoms follow conventional CPK coloring.

Group 4 metal oxo clusters are the building blocks of metal–organic frameworks (MOFs),^1–5^ while the discrete clusters are used in polymer composites,^6–8^ and as catalysts.^9–13^ Conceptually, discrete oxo clusters are close to metal oxide nanocrystals, see Scheme 1. Both have an inorganic core capped with an organic ligand shell, mostly carboxylates.^14,15^ One can regard the prototypical Zr_6_O_8_H_4_(OOCR)_12_ (Zr6) cluster as the smallest possible nanoparticle, with the advantage that the cluster is atomically precise while nanocrystals have a size distribution.

Zr6 clusters feature six zirconium atoms arranged in an octahedron and eight μ_3_-oxygen atoms, one on every facet of the octahedron. Half of these μ_3_-oxygens are protonated.^16,17^ The cationic core is coordinated by twelve carboxylate ligands (either nine in bridging and three in chelating or all in bridging mode).^14^ While Zr6 is most common, there are zirconium clusters with a nuclearity of 3–10, 12, 18, 26 or 36 depending on the organic ligands and reaction conditions.^14,18,19^Zr6 clusters can dimerize to Zr12 clusters through four intercluster bridging ligands, if the ligands provide little sterical hindrance.^12,14^ In such Zr12 clusters, there are 4 distinct types of ligand environments: chelating, belt bridging, intercluster bridging and inner-face bridging.^17^ Hafnium forms the same types of oxo clusters, following the same rules on dimerization.^12,14^ The octahedral oxo cluster motif is also relevant for other tetravalent metals such as cerium and thorium.^20–23^ Titanium is an exception in the tetravalent series due to its reluctance to support a coordination number of eight. It rather forms Ti_8_O_8_(OOCR)_18_,^24^ or Ti_6_O_6_(OR)_6_(OOCR)_6_ clusters.^25^

Colloidal nanocrystals constitute a broad materials class ranging from metal colloids,^26–29^ over quantum dots,^30,31^ to metal oxide nanocrystals.^14,32^ Their uses are equally broad from optoelectronic devices to biomedical applications.^33–36^ In each of these applications the surface chemistry plays a crucial role. Ligands can be either inorganic or organic,^37,38^ with the latter being mostly used in nanocrystal synthesis.^39,40^ The surface of oxide nanocrystals is highly similar to that of oxo clusters. The ability of the surface oxygen atoms to bind protons in nonpolar solvents was also demonstrated for oxide nanocrystals.^41,42^ On the other hand, anions or neutral Lewis bases coordinate to the metal sites of nanocrystal surfaces, and their binding is usually confirmed through nuclear magnetic resonance (NMR) spectroscopy.^38,43,44^ Focusing on nonpolar solvents and zirconium/hafnium oxides, the ligand binding affinity can be roughly ranked as phosphonic acids (RPO(OH)_2_) ≈ monoalkylphosphinic acids (R(H)PO(OH)) ≫ carboxylic acids (RCOOH).^45,46^ Highly acidic alkylphosphonic acids (p*K*_a1_ = 2.38, p*K*_a2_ = 7.74 in water) and moderately acidic alkylphosphinic acids (p*K*_a_ = 3.34 in water) bind stronger compared to their carboxylate counterparts (*e.g.*, p*K*_a_ of acetic acid = 4.74 in water).^47^ Neutral Lewis bases such as amines, alcohols, and phosphine oxides typically bind much weaker.^42,48,49^ In addition to the binding group, also sterics play a role, with sterically hindered ligands binding weakly or only at facet edges.^50,51^ Finally, nanocrystal surfaces have many different binding sites, each with their own equilibrium binding constant, rendering thermodynamic analyses highly complex.^52,53^

The atomically precise nature of oxo clusters is appealing for surface chemistry studies.^54^ Focusing on nonpolar solvents and zirconium/hafnium oxo clusters, only carboxylate ligands were explored.^15^ Carboxylate-for-carboxylate exchange was studied. It was shown that at room temperature, there is no interconversion between Zr6 and Zr12, while at elevated temperatures the ligand becomes structure-directing.^12,55^ The absence of any reports on ligand exchanges with phosphinic or phosphonic acids is striking. By direct synthesis from zirconium alkoxide and phosphonic acids, various oxoalkoxy clusters are formed instead of the Zr_6_O_8_ unit.^56,57^ Only from ZrCl_4_ and dimethylphosphate ligands in dimethylformamide, a Zr_6_O_8_ cluster was prepared.^58^ In case of titanium, only various oxoalkoxy species are produced by reacting titanium alkoxides with phosphonic or diphenylphosphinic acid.^59–62^ Additionally, Ce_6_O_8_ clusters were formed with mixed ligand shells consisting of eight pivalate and four diphenylphosphinate ligands.^63^ Overall, phosphorus-based ligands are rarely explored in the oxo cluster field.^64,65^ A Zr MOF with M_6_O_8_ as secondary building unit and phosphonate linkers was synthesized from their parent carboxylate MOF by solvent assisted linker exchange for a phosphinate ligand and subsequent oxidation to phosphonate.^66^

Here, we study the surface chemistry of Zr_6_O_8_ and Hf_6_O_8_ oxo clusters through ligand exchange reactions, both experimentally and computationally. Choosing carboxylate-capped Zr12 and Zr6 clusters as starting points, we modify their surface with a variety of phosphonate and mono/di-substituted phosphinate ligands in chloroform. The extent of exchange is monitored through solution ^1^H NMR, ^31^P NMR, and Fourier-transform infrared (FTIR) spectroscopy. The structural integrity of the core is assessed by X-ray total scattering and Pair Distribution Function (PDF) analysis. We find that a single exchange of a carboxylate for a phosphonate or phosphinate is thermodynamically favorable. However, full exchange of all 12 carboxylates per Zr_6_O_8_ core is sterically impeded for di-substituted phosphinic acids. Monoalkyl- or monoarylphosphinic acids quantitatively exchange all carboxylates while phosphonates cause disintegration of the Zr_6_O_8_ core and form zirconium phosphonate gels. In case of monophenylphosphinate-capped Zr6 clusters, single crystal X-ray diffraction (SCXRD) revealed the all-bridging binding mode of the ligand.

## Results

### *In silico* ligand exchange

**Fig. 1:**
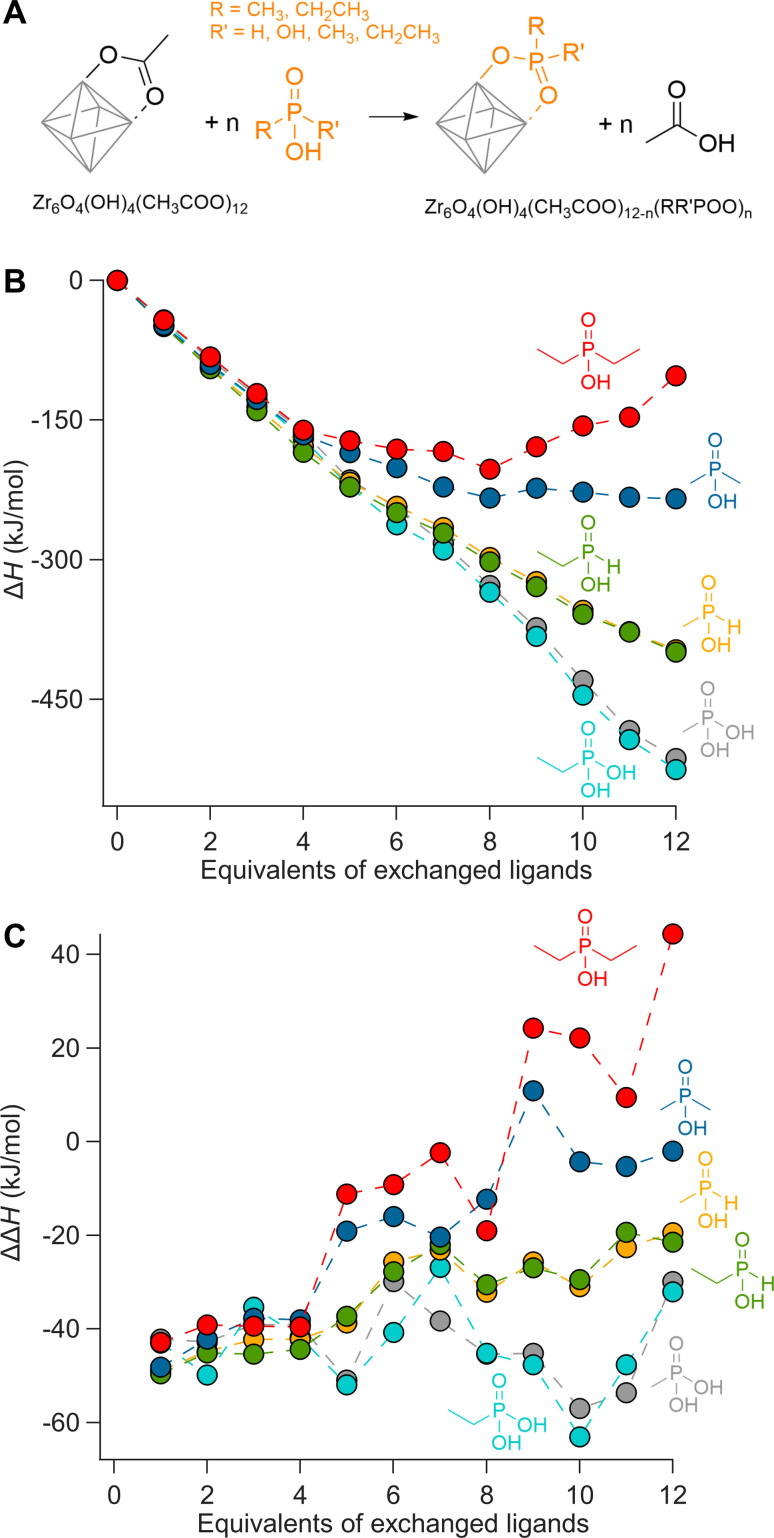
(A) Scheme representing the exchange of acetate ligands for phosphorus-based ligands on a fully bridged Zr6 cluster. (B) Enthalpy of ligand exchange reactions as a function of equivalents of exchanged ligands, Δ*H*. (C) The enthalpy change for every step, ΔΔ*H*.

We first evaluate ligand exchanges *in silico* by performing a set of density functional theory (DFT) calculations. We choose the crystal structure of Zr_6_O_8_H_4_(OOCMe)_12_ (Zr6-acetate) as the starting model due to its simplicity, featuring twelve identical bridging acetate ligands (Fig. S1†).^67^ The structure is an exception to the dimerization rule owing to its crystallization from an aqueous solution.^14^ We exchange the twelve acetate ligands in twelve steps, each time placing the new ligands on the cluster as far away as possible from each other to minimize interactions (Fig. S4–S9†). As incoming ligand, we explore ethyl and methylphosphonic acid, monoethyl and monomethylphosphinic acid, and diethyl and dimethylphosphinic acid (Fig. 1). The geometry of all structures are optimized at DFT/PBE/DZVP level of theory (more computational details in the Methods) and the Δ*H* for the exchanges are plotted in Fig. 1B. All optimized structures are available as xyz-files in the ESI.† The same calculations were performed for hafnium oxo clusters and the results are identical (Fig. S3†).

Phosphonic acids present a clear downhill path from the first to the last exchange and have an average; Δ*H* = −43 kJ per mole of exchanged ligand. Monoalkylphosphinic acids behave similarly but have a slightly lower average; Δ*H* = −33 kJ per mole of exchanged ligand. The difference between the average values for phosphonic and monoalkylphosphinic acids (10 kJ mol^−1^) is slightly larger than the experimentally determined free energy of exchange on hafnium oxide nanocrystals; Δ*G* = 2 kJ mol^−1^ in favor of phosphonic acids.^46^ Apart from entropic contributions, the lower energy of phosphonate-capped clusters can be attributed to the regular pattern of hydrogen bonds on the cluster (see Fig. S8 and S9†). There is little difference between the ethyl and methyl variants, giving confidence that longer chains will have little additional sterical impact. For dimethylphosphinic acid, the average exchange enthalpy is lower, Δ*H* = −20 kJ per mole of exchanged ligand. However, for the first four exchanges, the Δ*H* for each step (*i.e.*, ΔΔ*H*) is about the same as for monoalkylphosphinic acids (see Fig. 1C). From the fifth exchange, ΔΔ*H* becomes less negative and there is hardly any driving force for the last four exchanges. The effect is exacerbated for diethylphosphinic acid. The discontinuities at the fifth exchange and the ninth exchange are a consequence of our ligand positioning strategy. The first four exchanges happen on the edges of the equatorial plane of the Zr6 octahedron (Fig. S4†). The fifth incoming ligand cannot avoid interaction with the previously exchanged ligands. We predict that, experimentally, dialkylphosphinic acids will feature a mixed ligand shell with carboxylates. There is a clear thermodynamic minimum which lies at 8 exchanged ligands for diethylphosphinic acid. For other disubstituted phosphinic acids, its precise location will depend on the steric bulk of the substituents. This is in line with the literature reports on Ce6 oxo clusters with mixed ligand shells of pivalate and diphenylphosphinate.^63^ Comparing Zr6 clusters with nanocrystals, clusters have a higher surface curvature. Any particle larger than Zr6 has even less space to accommodate sterically hindered ligands, thus decreasing the potential ligand density of dialkylphosphinate ligands. Also for Ti4 clusters with an even higher curvature, the ligand shell is mixed with diphenylphosphinate and alkoxy ligands.^62,64^

Our theoretical approach clearly outlines that one common assumption in surface chemistry (*e.g.*, the Langmuir model) is invalid: that the adsorption energy of an adsorbate is independent of the surrounding adsorbates. Interestingly, every binding site is equal until the first ligand exchange happens. This is thus different from the previously observed binding site heterogeneity on CdSe,^52^ but rather agrees with composition-dependent thermodynamics.^53^ To assess the intrinsic binding affinity of different ligands, we thus compare the enthalpy change for the first exchanges only. Surprisingly, all studied ligands have about the same ΔΔ*H* for the first four exchanges; averaging −45 kJ mol^−1^.

**Fig. 2:**
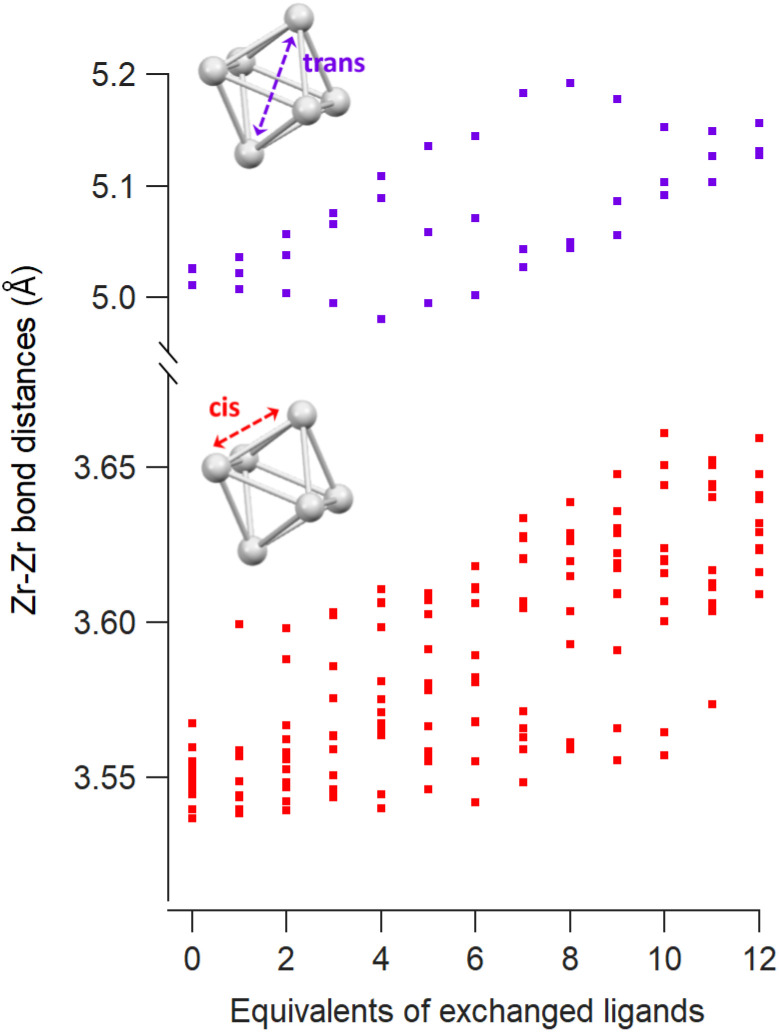
*Cis* and *trans* Zr–Zr distances as a function of the equivalents of exchanged methylphosphinate ligands obtained from DFT calculations.

The Zr6 core structure undergoes expansion during phosphinate/phosphonate ligand exchanges, a trend visualized by plotting all the *cis* and *trans* Zr–Zr distances within an oxo cluster core, see Fig. 2. When a first (bridging) acetate is exchanged with a (bridging) monomethylphosphinate, there is a clear outlier in the *cis* Zr–Zr distances, representing one longer distance of 3.6 Å. This data point is assigned to the Zr–Zr pair that is bridged by the phosphinate. The second exchange yields another long *cis* Zr–Zr distance, and there is asymmetry in the *trans* Zr–Zr distances with two longer distances and one shorter distance compared to the starting position. On average, both *cis* and *trans* distances are increasing with progressing ligand exchange. While the final structure is highly symmetric, the structures with mixed ligand shells are highly distorted and we find the analysis in Fig. 2 useful to objectively assess the structure and its symmetry. The analysis is made convenient through a python script that extracts all the distances, see Methods for more details. The above trend is consistent for both zirconium and hafnium clusters across all studied ligands (Fig. S10–S21†). We conclude that phosphinate and phosphonate ligands exert expansive strain onto the oxo clusters.

**Fig. 3:**
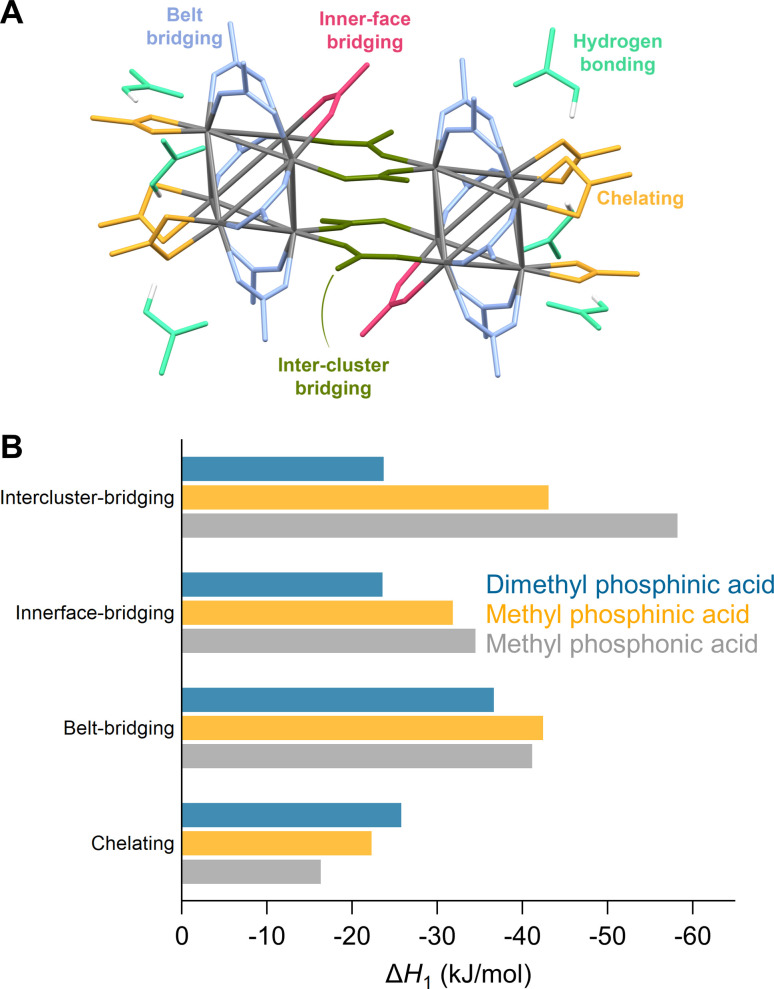
(A) Different binding modes of acetate ligands in the structure of Zr_12_O_16_H_8_(OOCMe)_24_·6MeCOOH – CCDC-604528.^5,17,55^ (B) Enthalpy of ligand exchange reactions at different binding sites.

When synthesized from nonaqueous solvents, the acetate ligands stabilize a Zr12 cluster, the dimer of two Zr6 clusters.^5,16,17^ This makes the ligand shell highly complex with multiple binding sites (Fig. 3A). To explore this complexity, we exchanged one acetate ligand at different positions for phosphinate and phosphonate ligands (Fig. 3B and S22†). All exchanges are exothermic but the extent depends on the binding site and the incoming ligand. The belt-bridging position provides generally the most favorable exchange whereas the inner-face bridging position clearly shows a smaller Δ*H* for the sterically hindered dimethylphosphinic acid. The intercluster-bridging position shows the largest variation being less exothermic for dimethylphosphinic acid and very exothermic for methylphosphonic acid. The latter makes hydrogen bonds with a remaining acetate ligand through the second acidic group, stabilizing the product. Exchanging a chelating acetate with a chelating phosphinate or phosphonate is the least exothermic. One can understand this through analysis of bond distances and bond angles. The chelating binding mode compresses the O–C–O bond angle of acetate from 122° (in free acetic acid) to 118°, yielding a strain of 3.3%. For methylphosphinate, the O–P–O bond angle is compressed from 116° to 106°, yielding a strain of 8.6%. The P–O bond distance is generally larger than the C–O bond distance due to the larger size of the phosphorus atom. This places the two oxygen atoms farther apart from one another in phosphinate (≈2.6 Å) than in carboxylate (≈2.3 Å). To obtain the best coordination environment, zirconium pulls the oxygen atoms together and achieves a distance of 2.20 Å for acetate and 2.45 Å for methylphosphinate. Furthermore, the Zr–O bond distance is shorter (*i.e.*, stronger) for chelating acetate (2.32 Å) compared to chelating methylphosphinate (2.37 Å). Hence the phosphinate ligand is more strained and Zr achieves a less favorable coordination environment, thus destabilizing the chelating mode for phosphinates. In contrast, in bridging acetate, the O–C–O angle expands from 122° to 126°, whereas the O–P–O bond angle experiences no strain from free (116°) to bound (116°) phosphinate. The Zr–O bond length is the same for bridging carboxylate and phosphinate (2.20 Å), which is stronger than the chelating mode.

Finally, we studied cluster dimerization by computing Δ*H* for the dimerization reaction. We find that the conversion of Zr6-acetate to Zr12-acetate is exothermic (Fig. S23†). However, the conversion of Zr6-methylphosphinate to Zr12-methylphosphinate is highly endothermic, particularly when the phosphinate ligands keep the original binding mode of the carboxylates. When the binding mode of the phosphinates is relaxed to the bridging configuration, the dimerization becomes less endothermic but is still unfavorable. We conclude that the calculation predicts a preference for the monomer upon exchanging carboxylate ligands for phosphinates.

### Ligand exchange for dialkylphosphinic acid

**Fig. 4:**
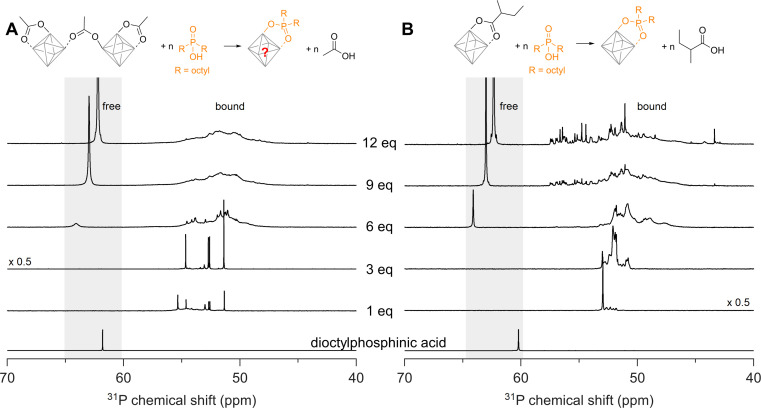
^31^P NMR spectra of the titrations of (A) Zr12-acetate and (B) Zr6-methylbutanoate with dioctylphosphinic acid (expressed as equivalents with respect to a monomer unit). The cluster concentration is 20 mg mL^−1^ in CDCl_3_. The reference ^31^P NMR spectrum of dioctylphosphinic acid with one equivalent acetic acid is also provided.

We now seek to confirm our computational results with experimental data and we first turn our attention to disubstituted phosphinic acids. As cluster model systems, we choose Zr12-acetate and Zr6-methylbutanoate, which were synthesized according to our earlier report.^12^Zr6-methylbutanoate features a ligand shell that includes both chelating and bridging ligands (Fig. S2†) whereas Zr12-acetate dimer exhibits a greater variety of binding modes, as shown in Fig. 3A.^12^ We titrate the clusters with dioctylphosphinic acid and express the latter's quantity as equivalents relative to a Zr_6_O_8_H_4_^12+^ octahedron (Fig. 4). There are twelve carboxylate ligands per Zr_6_O_8_H_4_^12+^ unit, both for the Zr6 and the Zr12 cluster. Upon addition of one equivalent of dioctylphosphinic acid to Zr12-acetate, one can discern six different resonances between 51 and 55 ppm in the ^31^P NMR spectrum (Fig. 4A). The different resonances are assigned to bound phosphinate and are a reflection of the binding site complexity on Zr12 clusters. The addition of one equivalent dioctylphosphinic acid to Zr6-methylbutanoate results in one predominant resonance at 53 ppm (Fig. 4B), indicating that the majority of bound phosphinate has the same environment. This is expected for the more symmetrical Zr6 cluster. The resonance for free dioctylphosphinic acid is absent in both cases, indicating a quantitative replacement of acetate, in line with the predictions by DFT.

Upon the addition of three equivalents dioctylphosphinic acid to Zr12-acetate, the most downfield resonance (at 55 ppm) disappears (Fig. 4A). The same result is found in the titration with diethylphosphinic acid (Fig. S24†) and from the ^1^H NMR spectrum (Fig. S25B†), we notice the disappearance of the characteristic pattern for Zr12-acetate. We infer that the cluster undergoes a structural reorganization, possibly into a Zr6 structure. For six equivalents of dioctylphosphinic acid, we observe the resonance of free phosphinic acid, which further increases in intensity for 9 and 12 equivalents (Fig. 4A). The same trend is observed for diethylphosphinic acid (Fig. S24B†), although the signal of free diethylphosphinic acid is less intense and appears only after 9 equivalents. This demonstrates that the composition of the mixed ligand shell depends on steric hindrance. For example, at 9 equivalents added, we integrate the free and bound resonances and derive that 7 dioctylphosphinates are bound, while 8.5 diethylphosphinates are bound. At 12 equivalents added, this becomes 7.4 bound dioctylphosphinates and 10 bound diethylphosphinates. Similar results are obtained for the Zr6-methylbutanoate clusters. For dialkylphosphinic acid, complete exchange is clearly not achieved, as predicted by DFT. Note that the resonance of free phosphinic acid is shifting slightly during the titration, which is due to its sensitivity to the acetic acid concentration (Fig. S26†).

### Ligand exchange for aryl or alkyl phosphinic acids

**Fig. 5:**
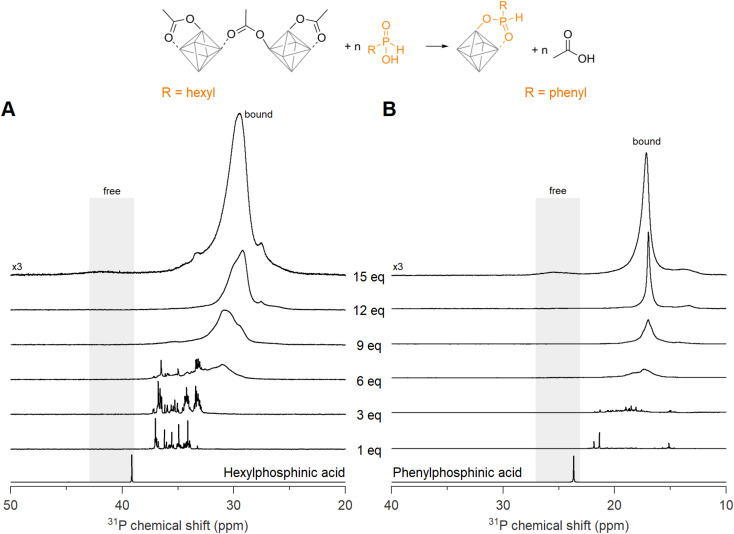
^31^P NMR spectra of the titrations of Zr12-acetate with (A) hexyl- and (B) phenylphosphinic acid. The cluster concentration is 20 mg mL^−1^ in CDCl_3_. ^31^P NMR spectra of the free phosphinic acids with an equivalent of acetic acid are provided as references.

Now we turn to the less sterically hindered monosubstituted phosphinic acids. We recently introduced them in nanocrystal synthesis,^68^ and showed that their binding affinity for nanocrystal surfaces is comparable with that of phosphonic acids.^46^ Here we titrate Zr12-acetate with phenyl- or hexylphosphinic acid (Fig. 5). Note that the ^31^P NMR spectrum has a shifted range compared to Fig. 4 due to the different chemical shift of monosubstituted phosphinic acids, but the spectral width is the same in both graphs. Upon titrating Zr12-acetate with hexylphosphinic acid, the first equivalent leads to multiple resonances, indicating a multitude of binding sites, similar to the titration with dioctylphosphinic acid. In contrast to dioctylphosphinic acid, we do not observe free hexylphosphinic acid at any point, indicating an irreversible and complete exchange. At 12 equivalents, the resonance of bound hexylphosphinate is more narrow (fwhm = 1.8 ppm, 372 Hz), compared to the resonance of bound dioctylphosphinate (fwhm = 3.2 ppm, 645 Hz), indicating a more homogeneous chemical environment for hexylphosphinate. The spectrum with 15 equivalents added shows a broad but discernible resonance for free hexylphosphinic acid (Fig. 5A and S30†). The observed broadening of this resonance can be attributed to the dynamic equilibrium between free and coordinated hexylphosphinic acid.^17^

A similar conclusion is reached for the titration with phenylphosphinic acid (Fig. 5B). At 12 equivalents, the resonance of bound phenylphosphinate is even narrower (fwhm = 0.5 ppm), indicating a highly symmetric cluster structure. Similar to hexylphosphinic acid, the spectrum with 15 equivalents added shows a broad resonance for free phenylphosphinic acid (Fig. S31†). For both hexyl and phenylphosphinic acid, the ^1^H NMR spectra (Fig. S27†) show the disappearance of the bound acetate resonances and only a singlet at ∼2 ppm, pertaining to free acetic acid, remains at the end of the titration. The above results are further generalized with titrations of Zr12-acetate with tetradecylphosphinic acid (Fig. S28†) and of Zr6-methylbutanoate with hexylphosphinic acid (Fig. S29†). The results are similar to the ones discussed above.

**Fig. 6:**
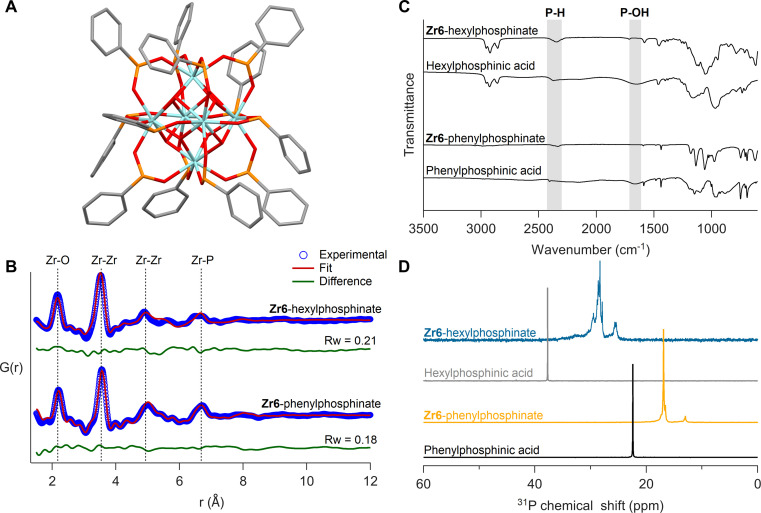
(A) Crystal structure of Zr6-phenylphosphinate cluster – Zr_6_O_8_H_4_(O_2_PHPh)_12_. Cyan atoms represent zirconium and all other atoms follow conventional CPK coloring. The co-crystallized dichloromethane molecules and hydrogen atoms are omitted for clarity. (B) PDF fit for Zr6-phenylphosphinate cluster with its crystal structure. PDF fit of Zr6-hexylphosphinate cluster with distorted Zr6 phosphinate cluster is also shown. (C) FTIR spectra of Zr6 phosphinate clusters. IR spectra of free ligands are also provided for reference. (D) ^31^P NMR of purified Zr6 phosphinate clusters. ^31^P NMR of free acids are provided as reference.

Given that monosubstituted phosphinic acids irreversibly exchange all twelve carboxylate ligands, we sought to isolate the fully phosphinate-capped clusters. The Zr12-acetate clusters were exchanged with 13.2 equivalents (per octahedron) of phenylphosphinic acid in dry DCM at room temperature. The free acetic acid was removed under vacuum and the phosphinate clusters were purified through precipitation. We obtained single crystals of Zr6-phenylphosphinate (CCDC-2358676) and Hf6-phenylphosphinate (CCDC-2358675). Single crystal XRD yielded unprecedented insight into the coordination mode of monoarylphosphinate adsorbed onto an oxide surface (see Fig. 6A, S32A and Table S1†). The M_6_O_8_H_4_^12+^ core is preserved with twelve phosphinate ligands binding to the cluster in bridging mode. No hydrogen-bonded phosphinic acid or chelating phosphinates were found, in agreement with our DFT calculations, and unlike the reported Zr6-benzoate cluster which has both chelating and bridging ligands.^69^ Compared to carboxylate-capped clusters, the metal–metal distances in the core slightly increase (by 1%), along with improved cluster symmetry upon exchange for phosphinate ligands (Fig. S33 and Table S2†). The DFT calculations predicted an expansion of 2%, thus slightly overestimating the core size.

To ensure that the bulk sample has the same structure as the single crystals, we analyzed the bulk powder with X-ray total scattering and PDF analysis (Fig. 6B). The PDF data was refined using the crystal structure as input model and we obtain a good fit (*R*_w_ = 0.18), confirming the sample's structural homogeneity. We could not crystallize Zr6-hexylphosphinate and thus analyzed its PDF. While the basic features of a Zr6 cluster are recognized, there are differences in the second Zr–Zr peak (5 Å) and the Zr–P peak (6.7 Å), indicating a higher degree of disorder in the hexylphosphinate-capped cluster. This agrees with the ^31^P NMR peak width analysis. We attempted to fit the PDF data of the hexylphosphinate-capped cluster with the structure of Zr6-phenylphosphinate and the result was unsatisfactory (Fig. S36 and Table S3†). The most significant misfit was observed for the Zr–Zr peak at ∼5 Å. This peak represents the longest Zr–Zr distance and is the distinctive feature of Zr6 clusters. Upon detailed examination, we conclude that the peak is split into two distinct peaks, probably due to a highly asymmetric core. For the same reason, the Zr–P peak at 6.6 Å is also broader and of lower intensity. We do not detect peaks at higher distances which would be characteristic for a dimer. Dynamic light scattering (DLS) experiments in comparison with Zr12-hexanoate clusters confirm that hexylphosphinate-capped clusters are indeed Zr6 (Fig. S40 and S41†), since the average solvodynamic radius is 0.91 nm for Zr12-hexanoate and 0.64 nm for Zr6-hexylphosphinate. We further refined the PDF to identify the most probable structure (Fig. S34†) by moving atoms within a chemically sensible range. The average Zr–Zr, Zr–O, and P–O bond distances in Zr6-phenylphosphinate are 3.58 Å, 2.21 Å and 1.50 Å, respectively, whereas those in the distorted Zr6-phosphinate structure are 3.53 Å, 2.17 Å and 1.47 Å. The asymmetry after core distortion is depicted in Fig. S35.† Similar results are obtained for tetradecylphosphinate-capped clusters (Fig. S37†). The hafnium clusters gave identical results, see Fig. S32B, S38, S39 and Table S4†.

The isolated clusters were also analyzed with FTIR and NMR spectroscopy (Fig. 6C and D). The references for free phosphinic acid were also given. In the FTIR spectrum, the broad P–OH stretch (1500–1850 cm^−1^) disappears upon binding and the P–H stretch (2350 cm^−1^) remains unaffected. In ^31^P NMR, the purified clusters feature no resonance of free phosphinic acid. Whereas Zr6-phenylphosphinate shows more narrow resonances compared to Zr6-hexylphosphinate, the pattern is largely the same: a downfield shoulder to the main peak and a smaller separate resonance upfield. The ^1^H NMR spectra show broadened resonances as expected for bound ligands (Fig. S42, S44 and S46†).^38^Zr6 and Hf6-phenylphosphinate clusters were further characterized with electrospray ionization-high resolution mass spectrometry (ESI-HRMS). Zr6-phenylphosphinate was detected as both proton adduct [M + H]^+^ and dehydroxylated ion [M − OH]^+^, with their isotopic patterns matching the simulated patterns (Fig. S48†). In contrast, Hf6-phenylphosphinate was detected as [Hf_6_O_4_(OH)_3_O(C_6_H_5_P(H)OO)_10_]^+^, after losing two ligands upon ionization (Fig. S49†). Ligand stripping experiments with trifluoroacetic acid,^68,70,71^ indicated a percentage of 0.4% and 0.07% acetate ligands in Zr6-hexylphosphinate and Zr6-tetradecylphosphinate, respectively, which confirms the complete ligand exchange (Fig. S51–S52†). The purified hafnium clusters showed similar results (Fig. S32C, D, S43, S45, and S47†).

### Ligand exchange with phosphonic acids

**Fig. 7:**
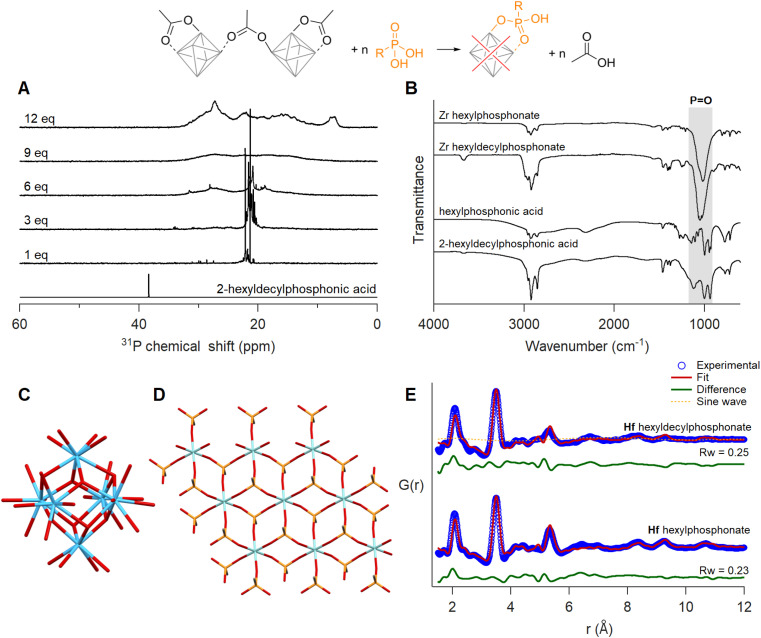
Ligand exchange of Zr12-acetate with 2-hexyldecylphosphonic acid. (A) ^31^P spectra of the titration, with the reference spectrum of 2-hexyldecylphosphonic acid with one equivalent of acetic acid added. The concentration of the cluster was 20 mg mL^−1^ in CDCl_3_. (B) IR spectra of phosphonate exchanged zirconium clusters after isolation and purification. IR spectra of free acids are provided as a reference. (C) Structure model of Hf6 cut from the crystal structure of Hf12-acetate. (D) Structure of a 3 × 3 layer zirconium phenylphosphonate (JPCDS: 44-2000). Cyan and blue atoms represent zirconium and hafnium, respectively; all other atoms follow conventional CPK coloring. Only the carbon bonded to phosphonate is shown, and the rest of the phenyl ring is omitted for clarity. (E) Dual-phase PDF fit for phosphonate exchanged Hf clusters with a 3 × 3 layer of Hf phenylphosphonate (contains 9 hafnium atoms in total) and Hf6 chelating bridging acetate.

When adding one equivalent of hexylphosphonic acid, a solution of Zr12-acetate immediately formed a gel. For 2-ethylhexylphosphonic acid and oleylphosphonic acid, the solution formed a gel after six equivalents (Fig. S53, S55 and S56†). Since the gelation was delayed with increasing steric bulk, we titrated Zr12-acetate with 2-hexyldecylphosphonic acid.^45^ No visible macroscopic gel was observed and the first equivalent results in two main narrow resonances in the ^31^P NMR spectrum (Fig. 7A). Upon addition of more phosphonic acid, the resonances broaden, and at 12 equivalents added, the bound phosphonates span a very large chemical shift range (26 ppm), even larger than typical for phosphonate-capped nanocrystals.^45^^1^H NMR showed the replacement of acetate by phosphonate ligands (Fig. S57†). The same gelation behaviour was observed when starting from Zr6-methylbutanoate (Fig. S54 and S58†). It is thus independent of the initial cluster structure and only depends on the phosphonate side chain.

Since phosphonic acids have two acidic protons, one phosphonic acid could replace two acetate ligands. This is uncommon on nanocrystal surfaces but observed for In_37_P_20_(OOCR)_51_ clusters.^72^ On the smaller Zr6 clusters, double deprotonation and binding to a single cluster would be associated with significant strain. It is however plausible that the second acidic group connects to a second cluster, resulting in a disordered network of clusters. This could explain the gelation behaviour. The ^31^P NMR spectrum does not feature free phosphonic acid upon addition of more than six equivalents, indicating an additional process and we hypothesize that the cluster decomposes and forms a new compound. To elucidate more structural information, the exchange product of Zr12-acetate with either hexyl or 2-hexyldecyl phosphonic acid, was purified by precipitation or trituration. The hexylphosphonate product was insoluble while the hexyldecylphosphonate product was soluble in organic solvents and showed broad signals in both ^1^H and ^31^P NMR (Fig. S61†). We investigated the phosphonate binding mode *via* infrared spectroscopy. The disappearance of two characteristic broad P–OH vibrations of free phosphonic acids around 2700–2100 cm^−1^ upon exchanging with clusters confirms their double deprotonation and coordination to the metal (Fig. 7B).^73^ The three absorption bands in the region 950–1200 cm^−1^ turn into one intense band at 1050 cm^−1^ upon exchange. The absence of the two flanking bands confirms that the phosphonate binds in tridentate mode.^74,75^ The strong band is assigned to the asymmetric stretch of phosphonate (P=O), and is characteristic for organozirconium phosphonate compounds.^76–78^ For example, zirconium phenylphosphonate is a layered compound with zirconium and tridentate phosphonate making up one layer and the phenyl substituents separating the layers, see Fig. 7D and S63.† Zirconium phosphonates are not molecular compounds but extended solids and are generally very insoluble, which would agree with our observation of gel formation.^79^

We analyzed the PDF of the equivalent phosphonate-exchanged hafnium oxo clusters, due to the higher atomic form factor of hafnium. The relative intensity of the first Hf–Hf and Hf–O peaks is too low for a Hf_6_O_8_ cluster. Therefore, we attempted to fit the experimental PDF with the reported structures of different cluster structures namely, Zr3-acetate isopropoxide,^80^Zr3-acetate *tert*-butoxide,^80^Zr4-formate isopropoxide,^80^Zr6-isobutyrate,^81^Zr6-acetate,^67^Zr12-acetate,^55^Zr10-salicylate,^82^ and Zr26-formate.^83^ For each of these structures, we replaced zirconium with hafnium prior to fitting. The models showed poor agreement with the experimental data, with a goodness of fit (*R*_w_) ranging from 0.61–0.87 (Fig. S64 and Table S5†). The most prominent peak around 3.5 Å fitted well for cluster models with Zr6 core or higher. The other atomic distances within the exchanged product align reasonably well with the reported structure of zirconium phenylphosphonate (JCPDS: 44-2000, CSD: WEBGEP),^84^ but there is a mismatch in the peak intensities. A single zirconium phenylphosphonate layer (containing 9 zirconium atoms, see Fig. 7D) provides a poor fit with an *R*_w_ of 0.73 (Fig. S65†). Finally, we explored a dual-phase fitting strategy in high throughput (Fig. 7E and Table S6†). The first phase is a metal phosphonate layer of various sizes (3 × 3 to 7 × 7) and the second phase is one of the clusters mentioned above. Out of over 200 combinations, we obtained the best fit where phase I is a 3 × 3 hafnium phosphonate layer and phase II a Hf6 core, extracted from the crystal structure of Hf12-acetate (Fig. 7C).^55^ Phosphonate exchanged zirconium clusters showed identical results, see Fig. S66, S67 and Table S7.† We conclude that upon ligand exchange, a significant fraction of the clusters decomposes into metal phosphonates, whereas the rest of the clusters get trapped in the gel with an intact M_6_O_8_ core.

Note that the exchange product with 2-hexyldecylphosphonic acid does not form a macroscopic gel. However, dynamic light scattering analysis detected particles with an average solvodynamic diameter of 22.47 nm (Fig. S68†). We thus conclude that the exchange product with 2-hexyldecylphosphonic acid forms amorphous nanoparticles of zirconium phosphonate and trapped Zr6 clusters, and the large steric bulk of the ligand precludes further gelation. Hafnium oxo clusters exchanged with either hexyl or 2-hexyldecylphosphonic acid also provide identical results, see Fig. S62, S69 and S70.†

## Discussion

We conclude that phosphonic acids are not suitable ligands for Zr6 or Hf6 oxo clusters. This behavior stands in contrast to the results on oxide nanocrystal surfaces where phosphonic acids were found to be excellent ligands with a high binding affinity. While larger sizes of ZrO_2_ crystals (*e.g.*, 3 nm) are stronger, the core of Zr6 clusters is still prone to restructuring. In addition, on nanocrystals, phosphonic acids were found to behave as monobasic ligands, exchanging only a single carboxylate per phosphonic acid equivalent.^46,53,85,86^ Interestingly for InP clusters, di-anionic binding of phosphonate was reported.^72^ For the few clusters with phosphonate ligands reported in the literature, the phosphonate is always present as a tridentate ligand. However, these are not Zr6 clusters but rather two Zr_3_O(μ_2_-OR)_3_(OR)_3_ units bridged by four phosphonates.^56,57^ Such a structure can be regarded as the first step of the decomposition of the Zr6 clusters where the two Zr3 units are pushed apart. The tridentate coordination of phosphonates was not considered in our theoretical calculations, neither was the decomposition of the oxo cluster. In the absence of experiments, one could have concluded that phosphonates were the most desirable ligands for oxo clusters. Finally, while Zr MOFs with phosphonate ligands are highly desired because of their superior stability, it is frequently hypothesized that they are difficult to make due to the poor reversibility of the phosphonate coordination bond.^87^ However, here we exposed the real reason; the Zr_6_O_8_ oxo cluster core is unstable in the presence of phosphonic acids.

Disubstituted phosphinic acids were also not suitable ligands to fully coordinate Zr6 oxo clusters since they are too sterically hindered. Only mixed ligand shells with less sterically hindered carboxylates are possible. Given that space is even more limited on flatter nanocrystal surfaces, we suggest that they are also poor ligands for nanocrystals. In the absence of stronger ligands, they can still bind, albeit with a lower ligand density.^49^ Monosubstituted phosphinic acids are the most promising ligands for oxo clusters. They are monobasic, have a high binding affinity and their binding mode is highly similar to that of carboxylates, with the difference that phosphinates prefer the bridging over the chelating mode.

## Conclusion

We have studied ligand exchange on atomically precise oxo clusters both computationally and experimentally. We find that phosphinates/phosphonates exhibit superior binding affinity compared to carboxylates. However, dialkylphosphinic acids can only partially cover the cluster surface due to sterical hindrance, which results in mixed-ligand shells. Monoalkyl- and monoarylphosphinates can quantitatively displace all carboxylates to yield fully phosphinate-capped clusters. We reported the crystal structures of the first phosphinate-capped zirconium and hafnium oxo clusters. Ligand exchange for phosphonates causes the partial decomposition of clusters to form metal phosphonates, ultimately forming a macroscopic or nanoscopic gel, depending on the sterical hindrance of the phosphonic acid. Even though metal oxo clusters are promising prototypes for oxide nanocrystals, we here identified important differences in their surface chemistries. Some insights are however transferable, for example the composition-dependent thermodynamics of ligand binding. Finally, these results gave important insights in MOFs syntheses and delineate the limited synthetic feasibility of Zr-phosphonate MOFs.

## Methods

### Materials

All chemical reagents and solvents were purchased from commercial sources and unless mentioned, used as received without further purification. Zirconium *n*-propoxide (70 w% in 1-propanol) and hafnium *n*-butoxide (99%) were purchased from Sigma-Aldrich and stored in a Straus flask upon arrival. Acetic acid (>99%) was purchased from Sigma-Aldrich and vacuum distilled to remove the absorbed water content. Methylbutanoic acid (98%), and hexylphosphonic acid (98%) were received from Sigma-Aldrich. Dry dichloromethane (99.8%), dry methanol (99.8%), dry tetrahydrofuran (99.5%), oleic acid (90%), and bromotrimethylsilane (98%) were purchased from Thermo Fisher Scientific. Acetone (100%), acetonitrile (99.9%), diethyl ether (99.5%), tetrahydrofuran (99.7%), anhydrous sodium sulfate (98%), and sodium hydroxide (97%) were obtained from VWR Chemicals. Lithium aluminium hydride (95%), Celite®545, triphenylphosphine (99%), sodium hypophosphite monohydrate (99%), potassium bisulfate, hexane (>97%), and dichloromethane (99.8%) were received from Sigma-Aldrich. Hexane (99%) and ethanol were bought from Honeywell Research Chemicals and Biosolve, respectively. 1-Hexene (97%), 1-tetradecene (90%), tetrabromomethane (99%), and triethylborane (1 M in THF, 11%) were purchased from TCI Chemicals. Diethylphosphinic acid (97%) was received from BLDPharm. Deuterated chloroform (CDCl_3_, 99.8%) was received from Eurisotop and treated with 4 Å molecular sieves (Sigma-Aldrich) for 24 hours before use. Milli-Q® water (resistivity of 18.2 MΩ cm at 25 °C) was dispensed from Merck Millipore Advantage A10 Water Purification System with Qpod. For size exclusion chromatography, Bio-Beads S-X3 was purchased from BIO-RAD.

### General instrumentation

Nuclear magnetic resonance (NMR) spectra were recorded at 298.15 K on a Bruker UltraShield 500 spectrometer operating at a ^1^H frequency of 500.13 MHz. Regular ^1^H and ^31^P NMR spectra were acquired using the standard pulse sequences with a 30° pulse (and a recycle delay of 1.5 and 1.0 seconds) from the Bruker library; zg30, zgpg30 respectively. ^1^H NMR spectra were acquired with 64 scans and post-processed with a line broadening of 1 Hz. ^31^P{^1^H} NMR spectra were acquired using inverse gated decoupling with 4096 scans, and processed with a line broadening of 2 Hz to reduce noise. All spectra are background-corrected. Chemical shifts (*δ*) are given in parts per million (ppm), and the residual solvent peak was used as an internal standard (CDCl_3_: *δ*_H_ = 7.26 ppm). The chemical shifts for other nuclei were referenced indirectly to the ^1^H NMR frequency of the sample with the “xiref”-macro in Topspin. The IR spectra were recorded on a PerklinElmer spectrum 2 ATR-FTIR with a diamond crystal.

### Synthesis of Zr/Hf oxo clusters, phosphonic and phosphinic acids

The Zr12-acetate (Zr_12_O_16_H_8_(OOCMe)_24_·6MeCOOH·3.5CH_2_Cl_2_), Hf12-acetate (Hf_12_O_16_H_8_(OOCMe)_24_·6MeCOOH·3CH_2_Cl_2_) and Zr6-methylbutanoate clusters (Zr_6_O_8_H_4_(OOCCH(CH_3_)C_2_H_5_)_12_) were synthesized according to Van den Eynden *et al.*^12^ 2-Ethylhexyl, 2-hexyldecyl and oleylphosphonic acids were synthesized according to the literature.^45,46^ Dioctylphosphinic acid was synthesized according to the procedure described by Wang *et al.*^88^ Phosphinic acid ligands (hexyl, tetradecyl) were synthesized according to the procedures of Dhaene *et al.*^68^

### Ligand exchange titrations

10–12 mg of Zr12-acetate cluster was dissolved in 500 μL CDCl_3_ in an NMR tube with moderate heating. In a second vial, 24 equivalents of phosphonic/phosphinic acid were dissolved in 120 μL CDCl_3_. 10 μL of the acid solution (2 equivalents per Zr12, 1 equivalent per Zr6 core) was added each time to the acetate cluster solution, and NMR (^1^H & ^31^P) spectra were recorded.

### Ligand exchange reactions

#### Zr6-phenylphosphinate cluster

500 mg (0.146 mmol, 1 eq.) of Zr12-acetate cluster was suspended in 10 mL dry dichloromethane in a glass vial. 546.47 mg (3.85 mmol, 1.1 eq. per acetate or 13.2 eq. per octahedron) of phenylphosphinic acid was added and stirred overnight at room temperature. The addition of phenylphosphinic acid made the cluster soluble in DCM. After the completion of the reaction, the mixture was evaporated under vacuum, dissolved in 5 mL of dry dichloromethane, stirred for 4 hours, and the product was precipitated with hexane. The precipitation was cycled thrice and finally, the product was dried *in vacuo*. Yield: 396.1 mg (57%). Zr_6_O_32_P_12_C_72_H_76_, found (calc.) C: 36.56 (36.45); H: 3.50 (3.23). Crystallization: the crystals were obtained through vapor diffusion technique. ∼25 mg of zirconium phenylphosphinate cluster was dissolved in 0.2 mL dry dichloromethane in a small vial. The vial was placed in a larger vial with 5 mL hexane, closed the lid, and kept undisturbed for over a week to obtain single crystals.

#### Hf6-phenylphosphinate cluster

100 mg (0.023 mmol, 1 eq.) of Hf12-acetate cluster was suspended in 2 mL dry dichloromethane in a glass vial. 84.5 mg (0.60 mmol, 1.1 eq. per acetate) of phenylphosphinic acid was added and stirred overnight at room temperature. The addition of phenylphosphinic acid made the cluster soluble in DCM. After the completion of the reaction, the mixture was evaporated under vacuum, dissolved in 3 mL of dry dichloromethane, and the product was precipitated with hexane. The precipitation was cycled thrice and finally, the product was dried *in vacuo*. Yield: 91.7 mg (70%). Hf_6_O_32_P_12_C_72_H_76_, found (calc.) C: 29.16 (29.86); H: 2.95 (2.65). Crystallization: the crystals were obtained through vapor diffusion technique. ∼25 mg of hafnium phenylphosphinate cluster was dissolved in 0.2 mL dry dichloromethane in a small vial. The vial was placed in a larger vial with 5 mL pentane, closed the lid, and kept undisturbed for over a week to obtain single crystals.

#### Zr6-hexylphosphinate cluster

200 mg (0.058 mmol, 1 eq.) of Zr12-acetate cluster was suspended in 4 mL dry dichloromethane in a glass vial. 231 mg (1.54 mmol, 1.1 eq. per acetate) of hexylphosphinic acid was added and stirred overnight at room temperature. The addition of hexylphosphinic acid made the cluster soluble in DCM. After the completion of the reaction, the mixture was evaporated under vacuum, followed by the addition of 2 mL dry DCM and stirring for 4 hours (equilibration). To remove excess ligands, the mixture was concentrated, and the solid was suspended in 2 mL acetonitrile, stirred overnight, collected through centrifugation, and dried *in vacuo* to obtain a white solid. Yield: 99 mg (34.4%). Zr_6_O_32_P_12_C_72_H_172_, found (calc.) C: 34.59 (35.02); H: 7.30 (7.02).

To remove H-bonded carboxylates, 0.5 equivalents of hexylphosphinic acid can be additionally added along with dry DCM while equilibration. Since the excess phosphinate makes the cluster soluble in acetonitrile, trituration-based purification is not possible. So final purification was performed with size exclusion chromatography. 15 g of Bio-Beads S-X3 was soaked in DCM overnight and subsequently packed in a column. 20 mg of the crude dissolved in 0.5 mL DCM was eluted.

#### Hf6-hexylphosphinate cluster

200 mg (0.045 mmol, 1 eq.) of Hf12-acetate cluster was suspended in 4 mL dry dichloromethane in a glass vial. 178.6 mg (1.19 mmol, 1.1 eq. per acetate) of hexylphosphinic acid was added and stirred overnight at room temperature. The addition of hexylphosphinic acid made the cluster soluble in DCM. After the completion of the reaction, the mixture was evaporated under vacuum, followed by the addition of 2 mL dry DCM and stirring for 4 hours. To remove excess ligands, the mixture was concentrated, and the solid was suspended in 2 mL acetonitrile, stirred overnight, collected through centrifugation, and dried *in vacuo* to obtain a white solid. Yield: 239 mg (88.6%). Hf_6_O_32_P_12_C_72_H_172_, found (calc.) C: 28.32 (28.90); H: 6.00 (5.79).

#### Zr6-tetradecylphosphinate cluster

200 mg (0.058 mmol, 1 eq.) of Zr12-acetate cluster was suspended in 4 mL dry dichloromethane in a glass vial. 404 mg (1.54 mmol, 1.1 eq. per acetate) of tetradecylphosphinic acid was added and stirred overnight at room temperature. The addition of tetradecylphosphinic acid made the cluster soluble in DCM. After the completion of the reaction, the mixture was evaporated under vacuum, followed by the addition of 2 mL dry DCM and stirring for 4 hours. The product was precipitated with acetonitrile. The precipitation was cycled thrice, and finally, the product was dried *in vacuo*. Yield: 215 mg (48.3%). Zr_6_O_32_P_12_C_168_H_364_, found (calc.) C: 53.39 (52.88); H: 9.93 (9.62).

To remove H-bonded carboxylates, 0.5 equivalents of tetradecylphosphinic acid can be additionally added along with dry DCM while equilibration.

#### Hf6-tetradecylphosphinate cluster

200 mg (0.045 mmol, 1 eq.) of Hf12-acetate cluster was suspended in 4 mL dry dichloromethane in a glass vial. 178.6 mg (1.19 mmol, 1.1 eq. per acetate) of tetradecylphosphinic acid was added and stirred overnight at room temperature. The addition of tetradecylphosphinic acid made the cluster soluble in DCM. After the completion of the reaction, the mixture was evaporated under vacuum, followed by the addition of 2 mL dry DCM and stirring for 4 hours. The product was precipitated with acetonitrile. The precipitation was cycled thrice and finally, the product was dried *in vacuo*. Yield: 329 mg (84.1%). Hf_6_O_32_P_12_C_168_H_364_, found (calc.) C: 46.88 (46.50); H: 8.72 (8.46).

#### Zr hexylphosphonate

200 mg (0.058 mmol, 1 eq.) of Zr12-acetate cluster was suspended in 4 mL dry dichloromethane in a glass vial. 256 mg (1.54 mmol, 1.1 eq. per acetate) of hexylphosphonic acid was added. Though the mixture gelled immediately, the stirring was continued overnight at room temperature. After the completion of the reaction, the mixture was evaporated under vacuum, followed by the addition of 2 mL dry DCM and stirring for 4 hours. To remove excess ligands, the mixture was concentrated, and the solid was suspended in 5 mL acetonitrile, stirred overnight, collected through centrifugation, and dried *in vacuo* to obtain a white solid. Yield: 249 mg.

#### Hf hexylphosphonate

200 mg (0.045 mmol, 1 eq.) of Hf12-acetate cluster was suspended in 4 mL dry dichloromethane in a glass vial. 198 mg (1.19 mmol, 1.1 eq. per acetate) of hexylphosphonic acid was added. Though the mixture gelled immediately, the stirring was continued overnight at room temperature. After the completion of the reaction, the mixture was evaporated under vacuum, followed by the addition of 2 mL dry DCM and stirring for 4 hours. To remove excess ligands, the mixture was concentrated, and the solid was suspended in 5 mL acetonitrile, stirred overnight, collected through centrifugation, and dried *in vacuo* to obtain a white solid. Yield: 287 mg.

#### Zr hexyldecylphosphonate

200 mg (0.058 mmol, 1 eq.) of Zr12-acetate cluster was suspended in 4 mL dry dichloromethane in a glass vial. 471 mg (1.54 mmol, 1.1 eq. per acetate) of 2-hexyldecylphosphinic acid dissolved in 2 mL dry dichloromethane was added and stirred overnight at room temperature. The addition of 2-hexyldecylphosphinic acid made the cluster soluble in DCM. After the completion of the reaction, the mixture was evaporated under vacuum to remove exchanged free acetic acid. The solid was redissolved in 2 mL of dry dichloromethane and stirred for 4 hours, and finally, the product was precipitated with acetone. The precipitation was cycled three times, and the product was dried under vacuum to yield a white solid. Yield: 395 mg.

#### Hf hexyldecylphosphonate

200 mg (0.045 mmol, 1 eq.) of Hf12-acetate cluster was suspended in 4 mL dry dichloromethane in a glass vial. 377 mg (1.19 mmol, 1.1 eq. per acetate) of 2-hexyldecylphosphinic acid dissolved in 2 mL dry dichloromethane was added and stirred overnight at room temperature. The addition of 2-hexyldecylphosphinic acid made the cluster soluble in DCM. After the completion of the reaction, the mixture was evaporated under vacuum to remove exchanged free acetic acid. The solid was redissolved in 2 mL of dry dichloromethane and stirred for 4 hours, and finally, the product was precipitated with acetone. The precipitation was cycled three times, and the product was dried under vacuum to yield a white solid. Yield: 386 mg.

### Mass spectrometry and elemental analysis

Electrospray ionization mass spectra (ESI-MS) were acquired using a Bruker maXis 4G high resolution mass spectrometer in positive ion mode. The cluster was dissolved in a 1 : 1 mixture of ACN : DCM. The samples were directly introduced into the instrument at a rate of 6 μL min^−1^ using a syringe pump. The heated capillary temperature was 180 °C and the capillary voltage was 34.5 kV. The raw data was processed with DataAnalysis 4.4 from Bruker. Simulations were carried out using enviPat.^89^

Quantitative elemental analysis was performed using Vario MICRO CUBE from Elementar.

### Dynamic light scattering analysis

Dynamic light scattering (DLS) measurements were performed on a Malvern Zetasizer Ultra Dynamic Light Scattering system in backscattering mode. 10 mg mL^−1^ solutions of clusters in chloroform after syringe filtering (PTFE 0.2 μm) were subjected to the analysis in a glass cuvette. All measurements were performed in triplicate at 25 °C after equilibrating inside the system for 240 seconds and the sample concentration was tuned to achieve system attenuator values between 9 and 10. For Zr6-hexylphosphinate and Zr12-hexanoate clusters, fitting was performed according to our previously reported procedure*.*^90^

### Ligand stripping experiments

20 mg of the cluster was dissolved in 400 μL CDCl_3_. 100 μL of trifluoroacetic acid was added to the solution. The mixture was centrifuged, the supernatant was collected, and NMR (^1^H and ^31^P) was recorded.

### DFT calculations

Computational calculations were performed with the CP2K program package.^91^ Cell parameters were optimized at the DFT level of theory with the hybrid Gaussian and plane waves (GPW) formalism and the Perdew–Burke–Ernzerhof (PBE) functionals.^92^ Goedecker–Teter–Hutter (GTH) pseudopotentials^93^ and the standard double-*ζ* MOLOPT basis sets (DZVP-MOLOPT-SR-GTH)^94^ have been used for all the atoms (Zr, Hf, C, H, O and P). The cutoff for the plane wave representation of electron density was set to 400 Ry, while the SCF convergence criterion was set to 1 × 10^−6^. All CP2K calculations were performed in vacuum without periodic boundary conditions using Wavelet Poisson solver.^95^ A box of 22 × 22 × 22 Å was used for Zr6 (dimensions: 12 × 12 × 12 Å), whereas a box of 33 × 33 × 33 Å was employed for Zr12-(dimensions: 17 × 22 × 23 Å) to achieve a zero electronic density at the edge of the box. The enthalpy of ligand exchange reactions was calculated as the difference between optimized energies of products and reactants in a balanced chemical equation. The bond lengths and bond angles of the optimized structures were extracted using the ‘xyz2tab′ python code.^96^

### Single crystal XRD

Single crystal data were collected on a STOE STADIVARI diffractometer. Suitable crystals were selected and mounted on a MITIGEN holder in perfluoroether oil. The crystals were kept at 150 K during data collection. Using Olex2,^97^ the structure was solved with the SHELXT^98^ structure solution program using Intrinsic Phasing and refined with the SHELXL^99^ refinement package using Least Squares minimisation.

### Synchrotron X-ray total scattering experiments

Samples were prepared in 1 mm polyimide Kapton tube and were measured either at beamline 11-ID-BM at Advanced Photon Source, Argonne National Laboratory, USA, beamline 28-ID-2 at National Synchrotron Light Source II, Brookhaven National Laboratory, USA or at beamline P21.1 at DESY in Hamburg, Germany. X-ray total scattering data were collected at room temperature in rapid acquisition mode, using a PerkinElmer digital X-ray flat panel amorphous silicon detector (2048 × 2048 pixels and 200 × 200 μm pixel size) with a sample-to-detector distance of 180 mm (11-ID-BM), 267 mm (28-ID-2) or 380 mm (P21.1). The incident wavelength of the X-rays was *λ* = 0.2110 Å (11-ID-BM), 0.1821 Å (28-ID-2) or 0.1220 Å (P21.1). Calibration of the experimental setup was performed using a Ni standard.

### Analysis of synchrotron X-ray total scattering data

Raw 2D data were corrected for geometrical effects and polarization, then azimuthally integrated to produce 1D scattering intensities *versus* the magnitude of the momentum transfer *Q* (where *Q* = 4π sin *θ*/*λ* for elastic scattering) using pyFAI and xpdtools.^100,101^ The program xPDFsuite with PDFgetX3 was used to perform the background subtraction, further corrections, and normalization to obtain the reduced total scattering structure function *F*(*Q*), and Fourier transformation to obtain the pair distribution function (PDF), *G*(*r*).^102,103^ For data reduction, the following parameters were used after proper background subtraction: *Q*_min_ = 0.8 Å ^−1^, *Q*_max_ = 22 Å ^−1^, *R*_poly_ = 0.9 Å. Modeling and fitting were carried out using Diffpy-CMI.^104^ The Debye scattering equation was used to generate the calculated PDF from discrete structure models. The structure models are supplied as xyz files in the ESI.† The refinements were carried out by refining the scale factor, isotropic atomic displacement parameters (Uiso), and delta2 (coefficient for 1/*r*^2^ contribution to the peak sharpening). The exponentially dampening sine-wave contribution was calculated according to the following equation.
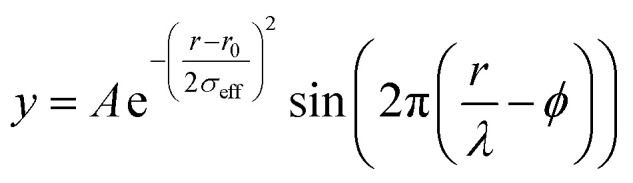
*A* – amplitude of oscillation, *r* – the distance in PDF, *λ* – wavelength, *ϕ* – phase shift, *σ* – effective dampening with *σ*_eff_ = *σ*/*a* for *r* < *r*_0_ and *σ*_eff_ = *σ* × *a* for *r* > *r*_0_*r*_0_, *a* is the asymmetry parameter. *r*_0_ is not a physical parameter in real space and is used to describe different dampening behavior.^105^

Powder XRD patterns were also extracted in the same way using pyFAI and xpdtools. The simulated powder patterns were generated using CCDC Mercury (*λ* = 0.1821 Å, same as experimental wavelength).^106^

## Data availability

The data underlying the figures is available on the Zenodo platform, DOI: https://doi.org/10.5281/zenodo.11484643. Details of DFT calculations, NMR titrations, ligand stripping experiments, NMR, FTIR and DLS data of purified compounds, and PDF refinements are provided in ESI.† The ESI† also contains xyz files of DFT calculated structures, crystallographic data of new metal oxo clusters, python code to extract bond distances and cross fit PDF data.

## Author contributions

A. R. Unniram Parambil: methodology, investigation, analysis, visualization, writing – original draft; R. Pokratath: PDF analysis; M. J. Parammal: PDF analysis, visualization; E. Dhaene: investigation; D. Van den Eynden: methodology; S. Balog: DLS analysis; A. Prescimone: SCXRD data acquisition; I. Infante: validation (DFT); P. Shahgaldian: supervision, funding acquisition; J. De Roo: conceptualization, supervision, funding acquisition, writing – review & editing, visualization.

## Conflicts of interest

There are no conflicts to declare.

## Supplementary Material

SC-OLF-D4SC03859B-s001

SC-OLF-D4SC03859B-s002

SC-OLF-D4SC03859B-s003

SC-OLF-D4SC03859B-s004

## References

[cit1] Dan-Hardi M., Serre C., Frot T., Rozes L., Maurin G., Sanchez C., Férey G. (2009). A New Photoactive Crystalline Highly Porous Titanium(IV) Dicarboxylate. J. Am. Chem. Soc..

[cit2] Cavka J. H., Jakobsen S., Olsbye U., Guillou N., Lamberti C., Bordiga S., Lillerud K. P. (2008). A New Zirconium Inorganic Building Brick Forming Metal Organic Frameworks with Exceptional Stability. J. Am. Chem. Soc..

[cit3] Fidelli A. M., Karadeniz B., Howarth A. J., Huskić I., Germann L. S., Halasz I., Etter M., Moon S.-Y., Dinnebier R. E., Stilinović V., Farha O. K., Friščić T., Užarević K. (2018). Green and rapid mechanosynthesis of high-porosity NU- and UiO-type metal–organic frameworks. Chem. Commun..

[cit4] Dai S., Simms C., Dovgaliuk I., Patriarche G., Tissot A., Parac-Vogt T. N., Serre C. (2021). Monodispersed MOF-808 Nanocrystals Synthesized via a Scalable Room-Temperature Approach for Efficient Heterogeneous Peptide Bond Hydrolysis. Chem. Mater..

[cit5] Bezrukov A. A., Törnroos K. W., Le Roux E., Dietzel P. D. C. (2018). Incorporation of an intact dimeric Zr_12_ oxo cluster from a molecular precursor in a new zirconium metal–organic framework. Chem. Commun..

[cit6] Schubert U. (2001). Polymers Reinforced by Covalently Bonded Inorganic Clusters. Chem. Mater..

[cit7] Gross S. (2011). Oxocluster-Reinforced Organic–Inorganic Hybrid Mater: Effect of Transition Metal Oxoclusters on Structural and Functional Properties. J. Mater. Chem..

[cit8] Murali M., Berne D., Joly-Duhamel C., Caillol S., Leclerc E., Manoury E., Ladmiral V., Poli R. (2022). Coordination Adaptable Networks: Zirconium(IV) Carboxylates. Chem.–Eur. J..

[cit9] Faccioli F., Bauer M., Pedron D., Sorarù A., Carraro M., Gross S. (2015). Hydrolytic Stability and Hydrogen Peroxide Activation of Zirconium-Based Oxoclusters. Eur. J. Inorg. Chem..

[cit10] Moons J., de Azambuja F., Mihailovic J., Kozma K., Smiljanic K., Amiri M., Cirkovic Velickovic T., Nyman M., Parac-Vogt T. N. (2020). Discrete Hf_18_ Metal-oxo Cluster as a Heterogeneous Nanozyme for Site-Specific Proteolysis. Angew. Chem., Int. Ed..

[cit11] Zhang Y., de Azambuja F., Parac-Vogt T. N. (2022). Zirconium oxo clusters as discrete molecular catalysts for the direct amide bond formation. Catal. Sci. Technol..

[cit12] Van den Eynden D., Pokratath R., Mathew J. P., Goossens E., De Buysser K., De Roo J. (2023). Fatty acid capped, metal oxo clusters as the smallest conceivable nanocrystal prototypes. Chem. Sci..

[cit13] Pulparayil Mathew J., Seno C., Jaiswal M., Simms C., Reichholf N., Van den Eynden D., Parac-Vogt T. N., De Roo J. (2024). The Central Role of Oxo Clusters in Zirconium-Based Esterification Catalysis. Small Sci..

[cit14] Van den Eynden D., Pokratath R., De Roo J. (2022). Nonaqueous Chemistry of Group 4 Oxo Clusters and Colloidal Metal Oxide Nanocrystals. Chem. Rev..

[cit15] Schubert U. (2017). Surface chemistry of carboxylato-substituted metal oxo clusters – Model systems for nanoparticles. Coord. Chem. Rev..

[cit16] Kickelbick G., Schubert U. (1997). Oxozirconium Methacrylate Clusters: Zr_6_(OH)_4_O_4_(OMc)_12_ and Zr_4_O_2_(OMc)_12_ (OMc = Methacrylate). Ber. Dtsch. Chem. Ges..

[cit17] Murali M., Bijani C., Daran J.-C., Manoury E., Poli R. (2023). Acetate exchange mechanism on a Zr_12_ oxo hydroxo cluster: relevance for reshaping Zr–carboxylate coordination adaptable networks. Chem. Sci..

[cit18] Choi J. I., Moon D., Chun H. (2021). Static and Dynamic Adsorptions of Water Vapor by Cyclic [Zr_36_] Clusters: Implications for Atmospheric Water Capture Using Molecular Solids. Bull. Korean Chem. Soc..

[cit19] Zhang Y., de Azambuja F., Parac-Vogt T. N. (2021). The forgotten chemistry of group(IV) metals: A survey on the synthesis, structure, and properties of discrete Zr(IV), Hf(IV), and Ti(IV) oxo clusters. Coord. Chem. Rev..

[cit20] Mereacre V., Ako A. M., Akhtar M. N., Lindemann A., Anson C. E., Powell A. K. (2009). Homo- and Heterovalent Polynuclear Cerium and Cerium/Manganese Aggregates. Helv. Chim. Acta.

[cit21] Takao S., Takao K., Kraus W., Emmerling F., Scheinost A. C., Bernhard G., Hennig C. (2009). First Hexanuclear U^IV^ and Th^IV^ Formate Complexes – Structure and Stability Range in Aqueous Solution. Eur. J. Inorg. Chem..

[cit22] Das R., Sarma R., Baruah J. B. (2010). A hexanuclear cerium(IV) cluster with mixed coordination environment. Inorg. Chem. Commun..

[cit23] Knope K. E., Wilson R. E., Vasiliu M., Dixon D. A., Soderholm L. (2011). Thorium(IV) Molecular Clusters with a Hexanuclear Th Core. Inorg. Chem..

[cit24] Frot T., Cochet S., Laurent G., Sassoye C., Popall M., Sanchez C., Rozes L. (2010). Ti_8_O_8_(OOCR)_16_, a New Family of Titanium–Oxo Clusters: Potential NBUs for Reticular Chemistry. Eur. J. Inorg. Chem..

[cit25] Hong K., Bak W., Chun H. (2014). Robust Molecular Crystals of Titanium(IV)-oxo-Carboxylate Clusters Showing Water Stability and CO_2_ Sorption Capability. Inorg. Chem..

[cit26] Turkevich J., Stevenson P. C., Hillier J. (1951). A study of the nucleation and growth processes in the synthesis of colloidal gold. Discuss. Faraday Soc..

[cit27] Brust M., Walker M., Bethell D., Schiffrin D. J., Whyman R. (1994). Synthesis of thiol-derivatised gold nanoparticles in a two-phase Liquid–Liquid system. J. Chem. Soc., Chem. Commun..

[cit28] Lee P. C., Meisel D. (1982). Adsorption and surface-enhanced Raman of dyes on silver and gold sols. J. Phys. Chem..

[cit29] Kang H., Buchman J. T., Rodriguez R. S., Ring H. L., He J., Bantz K. C., Haynes C. L. (2019). Stabilization of Silver and Gold Nanoparticles: Preservation and Improvement of Plasmonic Functionalities. Chem. Rev..

[cit30] Murray C. B., Norris D. J., Bawendi M. G. (1993). Synthesis and characterization of nearly monodisperse CdE (E = S, Se, Te) semiconductor nanocrystallites. J. Am. Chem. Soc..

[cit31] Efros A. L., Brus L. E. (2021). Nanocrystal Quantum Dots: From Discovery to Modern Development. ACS Nano.

[cit32] Niederberger M., Garnweitner G. (2006). Organic reaction pathways in the nonaqueous synthesis of metal oxide nanoparticles. Chem.–Eur. J..

[cit33] Shim M., Guyot-Sionnest P. (2000). n-type colloidal semiconductor nanocrystals. Nature.

[cit34] Yuan M., Liu M., Sargent E. H. (2016). Colloidal quantum dot solids for solution-processed solar cells. Nat. Energy.

[cit35] Frederix F., Friedt J.-M., Choi K.-H., Laureyn W., Campitelli A., Mondelaers D., Maes G., Borghs G. (2003). Biosensing Based on Light Absorption of Nanoscaled Gold and Silver Particles. Anal. Chem..

[cit36] Zimmer J. P., Kim S.-W., Ohnishi S., Tanaka E., Frangioni J. V., Bawendi M. G. (2006). Size Series of Small Indium Arsenide-Zinc Selenide Core-Shell Nanocrystals and Their Application to In Vivo Imaging. J. Am. Chem. Soc..

[cit37] Wang W., Zhang M., Pan Z., Biesold G. M., Liang S., Rao H., Lin Z., Zhong X. (2022). Colloidal Inorganic Ligand-Capped Nanocrystals: Fundamentals, Status, and Insights into Advanced Functional Nanodevices. Chem. Rev..

[cit38] De Roo J. (2023). The Surface Chemistry of Colloidal Nanocrystals Capped by Organic Ligands. Chem. Mater..

[cit39] Yin Y., Alivisatos A. P. (2005). Colloidal nanocrystal synthesis and the organic-inorganic interface. Nature.

[cit40] De Roo J. (2022). Chemical Considerations for Colloidal Nanocrystal Synthesis. Chem. Mater..

[cit41] De Roo J., Van den Broeck F., De Keukeleere K., Martins J. C., Van Driessche I., Hens Z. (2014). Unravelling the Surface Chemistry of Metal Oxide Nanocrystals, the Role of Acids and Bases. J. Am. Chem. Soc..

[cit42] De Roo J., Justo Y., De Keukeleere K., Van den Broeck F., Martins J. C., Van Driessche I., Hens Z. (2015). Carboxylic-Acid-Passivated Metal Oxide Nanocrystals: Ligand Exchange Characteristics of a New Binding Motif. Angew. Chem., Int. Ed..

[cit43] De Roo J., Yazdani N., Drijvers E., Lauria A., Maes J., Owen J. S., Van Driessche I., Niederberger M., Wood V., Martins J. C. (2018). *et al.*, Probing Solvent–Ligand Interactions in Colloidal Nanocrystals by the NMR Line Broadening. Chem. Mater..

[cit44] Hens Z. (2023). Ligands on Nanocrystal Surfaces, the ^1^H Nuclear Magnetic Resonance Fingerprint. Acc. Chem. Res..

[cit45] De Roo J., Zhou Z., Wang J., Deblock L., Crosby A. J., Owen J. S., Nonnenmann S. S. (2018). Synthesis of Phosphonic Acid Ligands for Nanocrystal Surface Functionalization and Solution Processed Memristors. Chem. Mater..

[cit46] Dhaene E., Coppenolle S., Deblock L., De Buysser K., De Roo J. (2023). Binding Affinity of Monoalkyl Phosphinic Acid Ligands toward Nanocrystal Surfaces. Chem. Mater..

[cit47] Edmundson R. , Properties and reactions of phosphonic and phosphinic acids and their derivatives, The Chemistry of Organophosphorus Compounds: Ter-and Quinque-Valent Phosphorus Acids and Their Derivatives, 1996, vol. 4, pp. 495–652

[cit48] De Roo J., Van Driessche I., Martins J. C., Hens Z. (2016). Colloidal Metal Oxide Nanocrystal Catalysis by Sustained Chemically Driven Ligand Displacement. Nat. Mater..

[cit49] De Keukeleere K., Coucke S., De Canck E., Van Der Voort P., Delpech F., Coppel Y., Hens Z., Van Driessche I., Owen J. S., De Roo J. (2017). Stabilization of Colloidal Ti, Zr, and Hf Oxide Nanocrystals by Protonated Tri-n-octylphosphine Oxide (TOPO) and Its Decomposition Products. Chem. Mater..

[cit50] Anderson N. C., Chen P. E., Buckley A. K., De Roo J., Owen J. S. (2018). Stereoelectronic Effects on the Binding of Neutral Lewis Bases to CdSe Nanocrystals. J. Am. Chem. Soc..

[cit51] De Nolf K., Cosseddu S. M., Jasieniak J. J., Drijvers E., Martins J. C., Infante I., Hens Z. (2017). Binding and Packing in Two-Component Colloidal Quantum Dot Ligand Shells: Linear versus Branched Carboxylates. J. Am. Chem. Soc..

[cit52] Drijvers E., De Roo J., Martins J. C., Infante I., Hens Z. (2018). Ligand Displacement Exposes Binding Site Heterogeneity on CdSe Nanocrystal Surfaces. Chem. Mater..

[cit53] Calvin J. J., O'Brien E. A., Sedlak A. B., Balan A. D., Alivisatos A. P. (2021). Thermodynamics of Composition Dependent Ligand Exchange on the Surfaces of Colloidal Indium Phosphide Quantum Dots. ACS Nano.

[cit54] Hens Z., De Roo J. (2020). Atomically Precise Nanocrystals. J. Am. Chem. Soc..

[cit55] Puchberger M., Kogler F. R., Jupa M., Gross S., Fric H., Kickelbick G., Schubert U. (2006). Can the Clusters Zr_6_O_4_(OH)_4_(OOCR)_12_ and [Zr_6_O_4_(OH)_4_(OOCR)_12_]_2_ Be Converted into Each Other?. Eur. J. Inorg. Chem..

[cit56] Chen G.-H., He Y.-P., Zhang S.-H., Zhang L. (2018). A series of zirconium-oxo cluster complexes based on arsenate or phosphonate ligands. Inorg. Chem. Commun..

[cit57] Czakler M., Schubert U. (2015). Phosphonate-substituted zirconium oxo clusters. Monatsh. Chem..

[cit58] Ji Z., Zhang H., Liu H., Yaghi O. M., Yang P. (2018). Cytoprotective metal-organic frameworks for anaerobic bacteria. Proc. Natl. Acad. Sci. U. S. A..

[cit59] Czakler M., Artner C., Schubert U. (2013). Influence of the Phosphonate Ligand on the Structure of Phosphonate-Substituted Titanium Oxo Clusters. Eur. J. Inorg. Chem..

[cit60] Guerrero G., Mehring M., Hubert Mutin P., Dahan F., Vioux A. (1999). Syntheses and single-crystal structures of novel soluble phosphonato- and phosphinato-bridged titanium oxo alkoxides. J. Chem. Soc., Dalton Trans..

[cit61] Mehring M., Guerrero G., Dahan F., Mutin P. H., Vioux A. (2000). Syntheses, Characterizations, and Single-Crystal X-ray Structures of Soluble Titanium Alkoxide Phosphonates. Inorg. Chem..

[cit62] Krämer T., Tuna F., Pike S. D. (2019). Photo-redox reactivity of titanium-oxo clusters: mechanistic insight into a two-electron intramolecular process, and structural characterisation of mixed-valent Ti(III)/Ti(IV) products. Chem. Sci..

[cit63] Russell-Webster B., Lopez-Nieto J., Abboud K. A., Christou G. (2021). Phosphorus-based ligand effects on the structure and radical scavenging ability of molecular nanoparticles of CeO_2_. Dalton Trans..

[cit64] Brown S. E., Warren M. R., Kubicki D. J., Fitzpatrick A., Pike S. D. (2024). Photoinitiated Single-Crystal to Single-Crystal Redox Transformations of Titanium-Oxo Clusters. J. Am. Chem. Soc..

[cit65] Pike S. D., White E. R., Shaffer M. S., Williams C. K. (2016). Simple phosphinate ligands access zinc clusters identified in the synthesis of zinc oxide nanoparticles. Nat. Commun..

[cit66] Xie H., Kirlikovali K. O., Chen Z., Idrees K. B., Islamoglu T., Farha O. K. (2024). A synthetic strategy towards single crystals of Zr_6_ cluster and phosphonate-based metal–organic frameworks. J. Mater. Chem. A.

[cit67] Hennig C., Weiss S., Kraus W., Kretzschmar J., Scheinost A. C. (2017). Solution Species and Crystal Structure of Zr(IV) Acetate. Inorg. Chem..

[cit68] Dhaene E., Pokratath R., Aalling-Frederiksen O., Jensen K. M. O., Smet P. F., De Buysser K., De Roo J. (2022). Monoalkyl Phosphinic Acids as Ligands in Nanocrystal Synthesis. ACS Nano.

[cit69] Kickelbick G., Wiede P., Schubert U. (1999). Variations in capping the Zr_6_O_4_(OH)_4_ cluster core: X-ray structure analyses of [Zr_6_(OH)_4_O_4_(OOC−CH=CH_2_)_10_]_2_(μ−OOC−CH=CH_2_)_4_ and Zr_6_(OH)_4_O_4_(OOCR)_12_(PrOH) (R=Ph, CMe =CH_2_). Inorg. Chim. Acta.

[cit70] Dhaene E., Billet J., Bennett E., Van Driessche I., De Roo J. (2019). The trouble with ODE: polymerization during nanocrystal synthesis. Nano Lett..

[cit71] Calcabrini M., Van den Eynden D., Ribot S. S., Pokratath R., Llorca J., De Roo J., Ibáñez M. (2021). Ligand Conversion in Nanocrystal Synthesis: The Oxidation of Alkylamines to Fatty Acids by Nitrate. JACS Au.

[cit72] Ritchhart A., Cossairt B. M. (2019). Quantifying Ligand Exchange on InP Using an Atomically Precise Cluster Platform. Inorg. Chem..

[cit73] Luschtinetz R., Seifert G., Jaehne E., Adler H.-J. P. (2007). Infrared Spectra of Alkylphosphonic Acid Bound to Aluminium Surfaces. Macromol. Symp..

[cit74] Gao W., Dickinson L., Grozinger C., Morin F. G., Reven L. (1996). Self-Assembled Monolayers of Alkylphosphonic Acids on Metal Oxides. Langmuir.

[cit75] Dufek E. J., Buttry D. A. (2009). Characterization of Zr(IV)–Phosphonate Thin Films Which Inhibit O_2_ Reduction on AA2024-T3. J. Electrochem. Soc..

[cit76] Frey B. L., Hanken D. G., Corn R. M. (1993). Vibrational spectroscopic studies of the attachment chemistry for zirconium phosphonate multilayers at gold and germanium surfaces. Langmuir.

[cit77] Hong H. G., Sackett D. D., Mallouk T. E. (1991). Adsorption of well-ordered zirconium phosphonate multilayer films on high surface area silica. Chem. Mater..

[cit78] Zeng R., Fu X., Gong C., Sui Y. (2006). Synthesis and catalytic application of zirconium-substituted aminoethyl phosphonate. J. Mater. Sci..

[cit79] Vivani R. , Costantino F. and Taddei M., Metal Phosphonate Chemistry: From Synthesis to Applications, The Royal Society of Chemistry, 2011

[cit80] Boyle T. J., Ottley L. A. M., Rodriguez M. A. (2005). Structurally characterized carboxylic acid modified zirconium alkoxides for the production of zirconium oxide thin films. Polyhedron.

[cit81] Kogler F. R., Jupa M., Puchberger M., Schubert U. (2004). Control of the ratio of functional and non-functional ligands in clusters of the type Zr_6_O_4_(OH)_4_(carboxylate)_12_ for their use as building blocks for inorganic–organic hybrid polymers. J. Mater. Chem..

[cit82] Kickelbick G., Schubert U. (1999). Hydroxy carboxylate substituted oxozirconium clusters. J. Chem. Soc., Dalton Trans..

[cit83] Nateghi B., Boldog I., Domasevitch K. V., Janiak C. (2018). More versatility than thought: large {Zr_26_} oxocarboxylate cluster by corner-sharing of standard octahedral subunits. CrystEngComm.

[cit84] Poojary M. D., Hu H.-L., Campbell III F. L., Clearfield A. (1993). Determination of crystal structures from limited powder data sets: crystal structure of zirconium phenylphosphonate. Acta Crystallogr., Sect. B: Struct. Sci..

[cit85] Gomes R., Hassinen A., Szczygiel A., Zhao Q., Vantomme A., Martins J. C., Hens Z. (2011). Binding of Phosphonic Acids to CdSe Quantum Dots: A Solution NMR Study. J. Phys. Chem. Lett..

[cit86] Knauf R. R., Lennox J. C., Dempsey J. L. (2016). Quantifying Ligand Exchange Reactions at CdSe Nanocrystal Surfaces. Chem. Mater..

[cit87] Gagnon K. J., Perry H. P., Clearfield A. (2012). Conventional and Unconventional Metal–Organic Frameworks Based on Phosphonate Ligands: MOFs and UMOFs. Chem. Rev..

[cit88] Wang F., Tang R., Buhro W. E. (2008). The Trouble with TOPO; Identification of Adventitious Impurities Beneficial to the Growth of Cadmium Selenide Quantum Dots, Rods, and Wires. Nano Lett..

[cit89] Loos M., Gerber C., Corona F., Hollender J., Singer H. (2015). Accelerated Isotope Fine Structure Calculation Using Pruned Transition Trees. Anal. Chem..

[cit90] Van den Eynden D. , Unniram Parambil A. R., Balog S. and De Roo J., Solution Characterization of Zirconium Oxo Clusters, ChemRxiv, 2024, preprint, 10.26434/chemrxiv-2024-4l8b2

[cit91] Kühne T. D. (2020). *et al.*, CP2K: An electronic structure and molecular dynamics software package - Quickstep: Efficient and accurate electronic structure calculations. J. Chem. Phys..

[cit92] Perdew J. P., Burke K., Ernzerhof M. (1996). Generalized Gradient Approximation Made Simple. Phys. Rev. Lett..

[cit93] Goedecker S., Teter M., Hutter J. (1996). Separable dual-space Gaussian pseudopotentials. Phys. Rev. B: Condens. Matter Mater. Phys..

[cit94] VandeVondele J., Hutter J. (2007). Gaussian basis sets for accurate calculations on molecular systems in gas and condensed phases. J. Chem. Phys..

[cit95] Genovese L., Deutsch T., Goedecker S. (2007). Efficient and accurate three-dimensional Poisson solver for surface problems. J. Chem. Phys..

[cit96] Dechert S. , xyz2tab, 2016, https://github.com/radi0sus/xyz2tab

[cit97] Dolomanov O. V., Bourhis L. J., Gildea R. J., Howard J. A. K., Puschmann H. (2009). *OLEX2*: a complete structure solution, refinement and analysis program. J. Appl. Crystallogr..

[cit98] Sheldrick G. M. (2015). *SHELXT* – Integrated space-group and crystal-structure determination. Acta Crystallogr., Sect. A: Found. Adv..

[cit99] Sheldrick G. M. (2015). Crystal structure refinement with *SHELXL*. Acta Crystallogr., Sect. C: Struct. Chem..

[cit100] Ashiotis G., Deschildre A., Nawaz Z., Wright J. P., Karkoulis D., Picca F. E., Kieffer J. (2015). The fast azimuthal integration Python library:pyFAI. J. Appl. Crystallogr..

[cit101] Wright C. J., Zhou X.-D. (2017). Computer-assisted area detector masking. J. Synchrotron Radiat..

[cit102] Juhas P., Davis T., Farrow C. L., Billinge S. J. L. (2013). PDFgetX3: a rapid and highly automatable program for processing powder diffraction data into total scattering pair distribution functions. J. Appl. Crystallogr..

[cit103] Yang X. , Juhas P., Farrow C. L. and Billinge S. J., xPDFsuite: an end-to-end software solution for high throughput pair distribution function transformation, visualization and analysis, arXiv, 2014, preprint, arXiv:1402.3163, 10.48550/arXiv.1402.3163

[cit104] Juhas P., Farrow C. L., Yang X., Knox K. R., Billinge S. J. L. (2015). Complex modeling: a strategy and software program for combining multiple information sources to solve ill posed structure and nanostructure inverse problems. Acta Crystallogr..

[cit105] Zobel M., Neder R. B., Kimber S. A. J. (2015). Universal solvent restructuring induced by colloidal nanoparticles. Science.

[cit106] Macrae C. F., Sovago I., Cottrell S. J., Galek P. T., McCabe P., Pidcock E., Platings M., Shields G. P., Stevens J. S., Towler M., Wood P. A. (2020). Mercury 4.0: From visualization to analysis, design and prediction. Appl. Crystallogr..

