# Supplementary material for: Atomically precise surface chemistry of zirconium and hafnium metal oxo clusters beyond carboxylate ligands
Source: Chem Sci. 2024 Oct 7;15(42):17380–96. doi: 10.1039/d4sc03859b (PMC11457152; doi:10.1039/d4sc03859b)
Supplement: SC-015-D4SC03859B-s003 [file SC-015-D4SC03859B-s003.pdf]

Supplementary Information (SI) for Chemical Science.
This journal is © The Royal Society of Chemistry 2024

Supporting Information: Atomically precise surface chemistry of zirconium and hafnium metal oxo clusters beyond carboxylate ligands

Ajmal Roshan Unniram Parambil,†,‡,¶ Rohan Pokratath,‡ Muhammed Jibin Parammal,‡ Evert Dhaene,‡ Dietger Van den Eynden,‡,§ Sandor Balog,‖ Alessandro Prescimone,‡ Ivan Infante,⊥ Patrick Shahgaldian,† and Jonathan De Roo*,‡

†Institute of Chemistry and Bioanalytics, School of Life Sciences, University of Applied Sciences and Arts Northwestern Switzerland, 4132 Muttenz, Switzerland

‡Department of Chemistry, University of Basel, Mattenstrasse 22, 4058 Basel, Switzerland

¶Swiss Nanoscience Institute, Klingelbergstrasse 82, 4056 Basel

§Department of Chemistry, University of Ghent, Krijgslaan 281, 9000 Ghent, Belgium

‖Adolphe Merkle Institute, University of Fribourg, 1700 Fribourg, Switzerland

⊥BCMaterials, Spain

E-mail: jonathan.deroo@unibas.ch

Contents

1 *In silico* ligand exchange **S-3**

2 Ligand exchange for dialkylphosphinic acid **S-18**

2.1 NMR titrations of clusters S-18

2.2 Reference spectra S-20

3 Ligand exchange for aryl or alkyl phosphinic acids **S-21**

3.1 NMR titrations of clusters S-21

3.2 Reference spectra S-24

3.3 Characterization of purified clusters S-25

3.4 Single crystal data S-26

3.5 PDF refinement data S-28

3.6 Dynamic light scattering analysis S-32

3.7 ^{1}H and ^{31}P NMR spectra of purified clusters S-33

3.8 ESI-HRMS analysis S-36

3.9 Powder X-ray diffraction data S-37

3.10 Ligand stripping experiments S-38

4 Ligand Exchange with phosphonic acids **S-40**

4.1 NMR titrations of clusters S-40

4.2 Reference spectra S-44

4.3 ^{1}H and ^{31}P NMR spectra of purified clusters S-45

4.4 PDF refinement data S-46

4.5 Dynamic light scattering analysis S-51

4.6 FT-IR spectra S-52

1 *In silico* ligand exchange

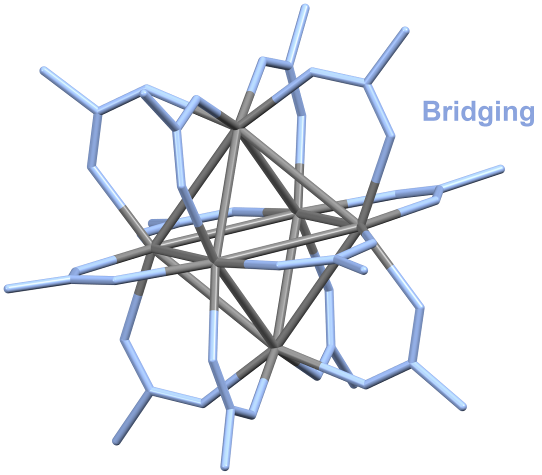

Figure S1: Crystal structure of **Zr6**-acetate cluster ($Zr_6O_4(OH)_4(OOCCH_3)_{12}$) - CCDC-1051013.[S1] All twelve carboxylate ligands are in bridging mode. Hydrogen atoms and core oxygen atoms are omitted for clarity.

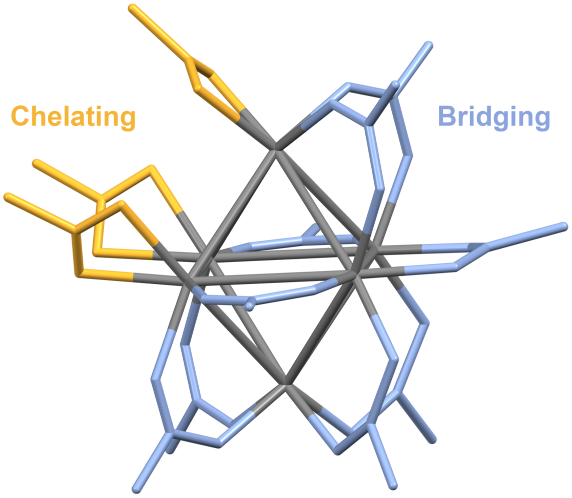

Figure S2: Crystal structure of **Zr6**-methacrylate cluster ($Zr_6O_4(OH)_4(OOC(CH_3)C{=}CH_2)_{12}$) - CCDC-106826.[S2] Ligand shell contains 9 bridging carboxylates and 3 chelating carboxylates. Hydrogen atoms, aliphatic chain carbon atoms and core oxygen atoms are omitted for clarity.

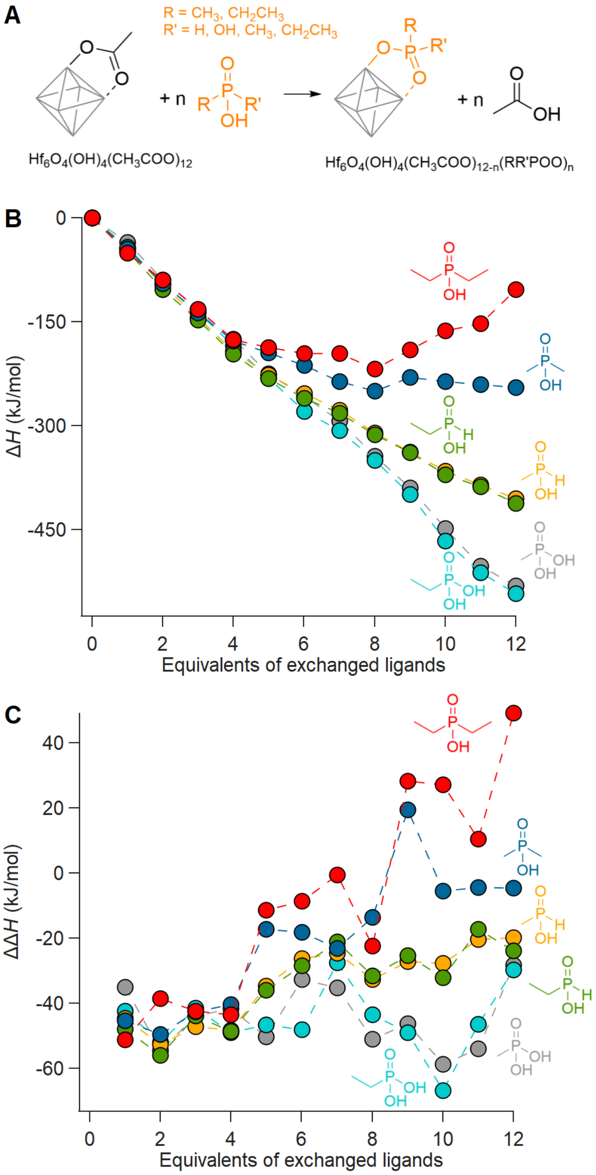

Figure S3: (A) Scheme representing the exchange of acetate ligands for phosphorus-based ligands on a fully bridged **Hf6** cluster. (B) Enthalpy of ligand exchange reactions as a function of equivalents of exchanged ligands, ΔH. (C) The enthalpy change for every step, $\Delta\Delta H$.

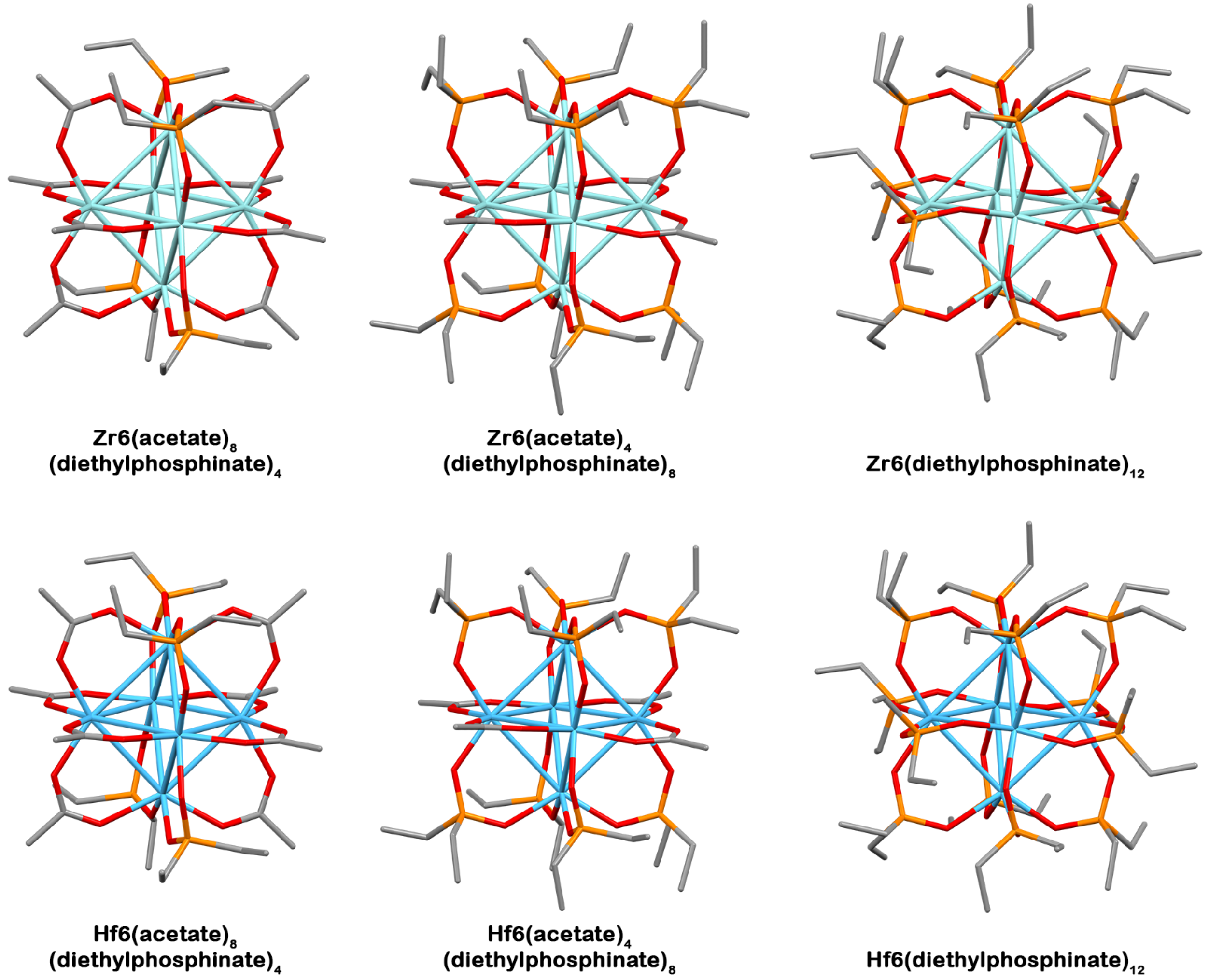

Figure S4: DFT optimized structures of Zr/Hf acetate clusters partially or fully exchanged with diethylphosphinic acid. Cyan and blue atoms represent zirconium and hafnium, respectively; all other atoms follow conventional CPK coloring. Hydrogen atoms and core oxygen atoms are omitted for clarity.

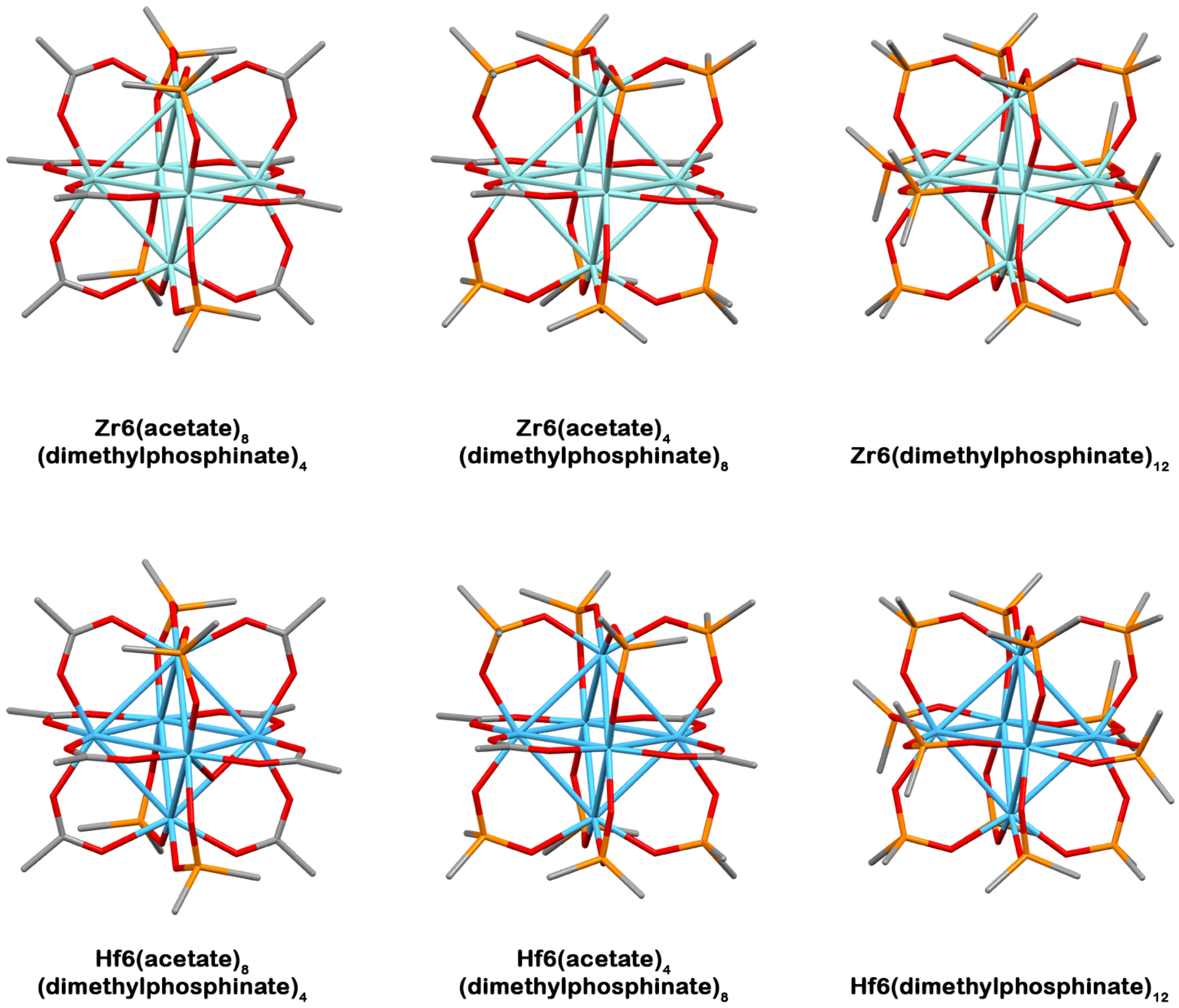

Figure S5: DFT optimized structures of Zr/Hf acetate clusters partially or fully exchanged with dimethylphosphinic acid. Cyan and blue atoms represent zirconium and hafnium, respectively; all other atoms follow conventional CPK coloring. Hydrogen atoms and core oxygen atoms are omitted for clarity.

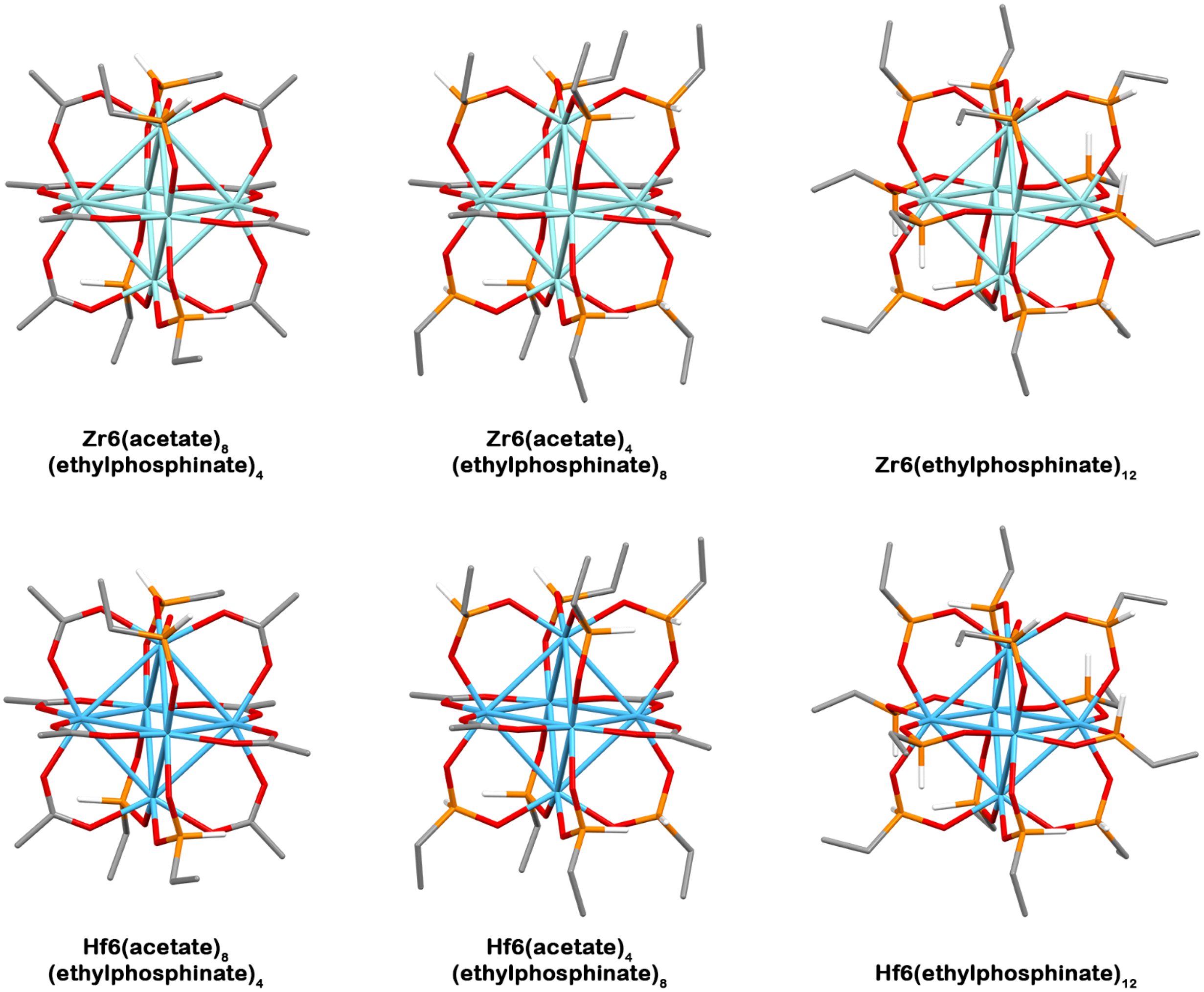

Figure S6: DFT optimized structures of Zr/Hf acetate clusters partially or fully exchanged with ethylphosphinic acid. Cyan and blue atoms represent zirconium and hafnium, respectively; all other atoms follow conventional CPK coloring. Hydrogen atoms (except P-H) and core oxygen atoms are omitted for clarity.

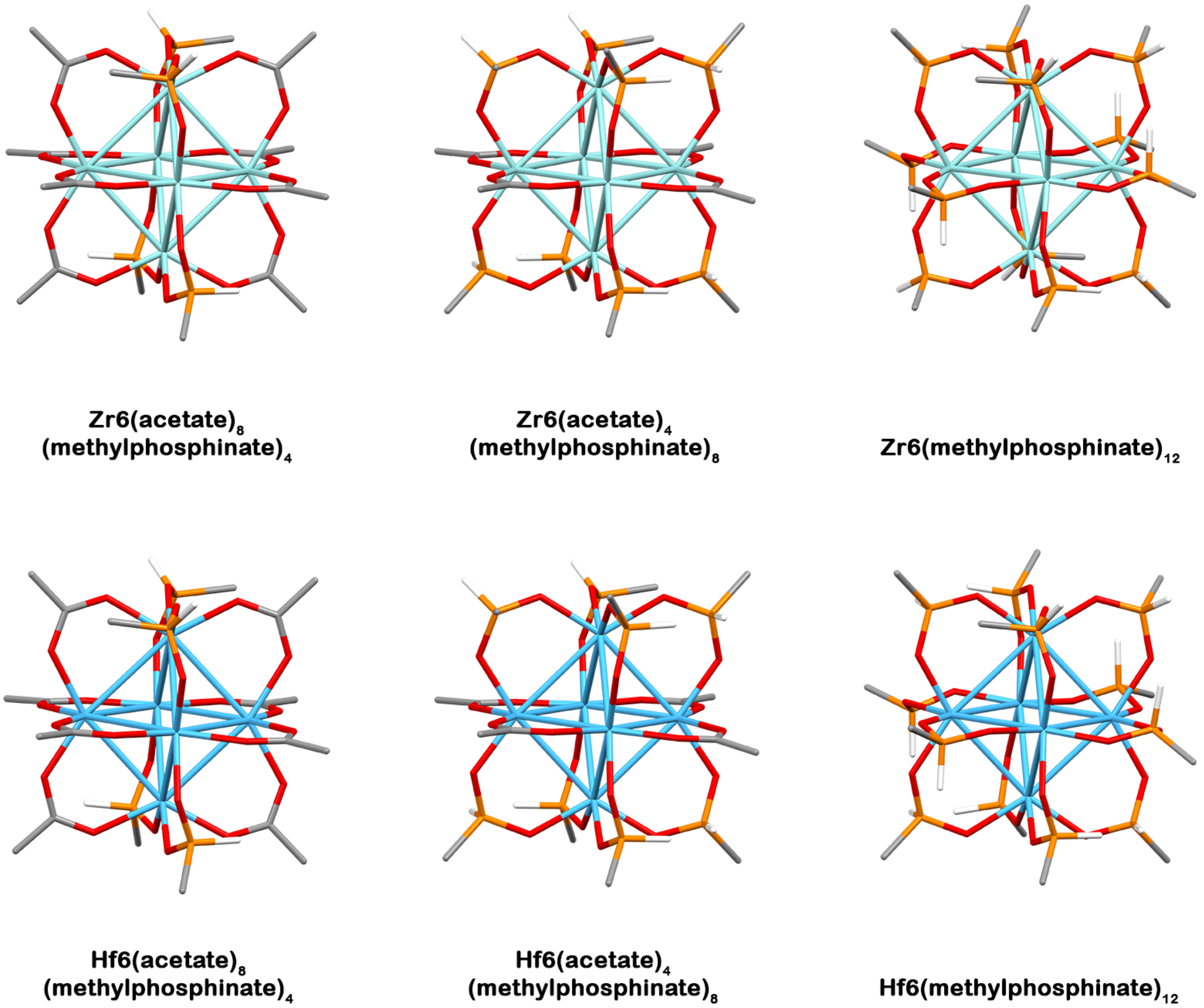

Figure S7: DFT optimized structures of Zr/Hf acetate clusters partially or fully exchanged with methylphosphinic acid. Cyan and blue atoms represent zirconium and hafnium, respectively; all other atoms follow conventional CPK coloring. Hydrogen atoms (except P-H) and core oxygen atoms are omitted for clarity.

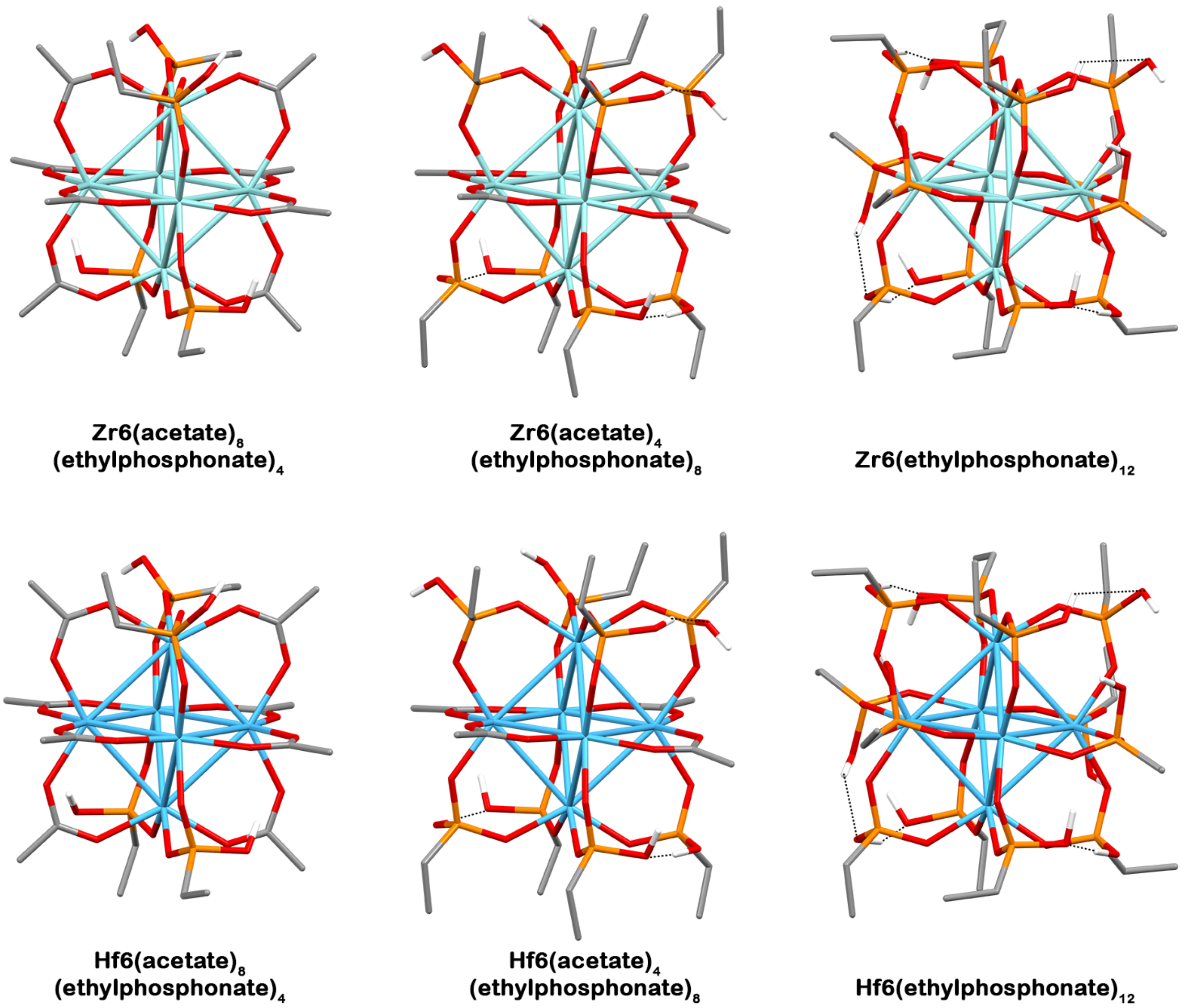

Figure S8: DFT optimized structures of Zr/Hf acetate clusters partially or fully exchanged with ethylphosphonic acid. Cyan and blue atoms represent zirconium and hafnium, respectively; all other atoms follow conventional CPK coloring. The dotted lines indicate some hydrogen bonds formed due to the second acidic group. Hydrogen atoms (except P-OH) and core oxygen atoms are omitted for clarity.

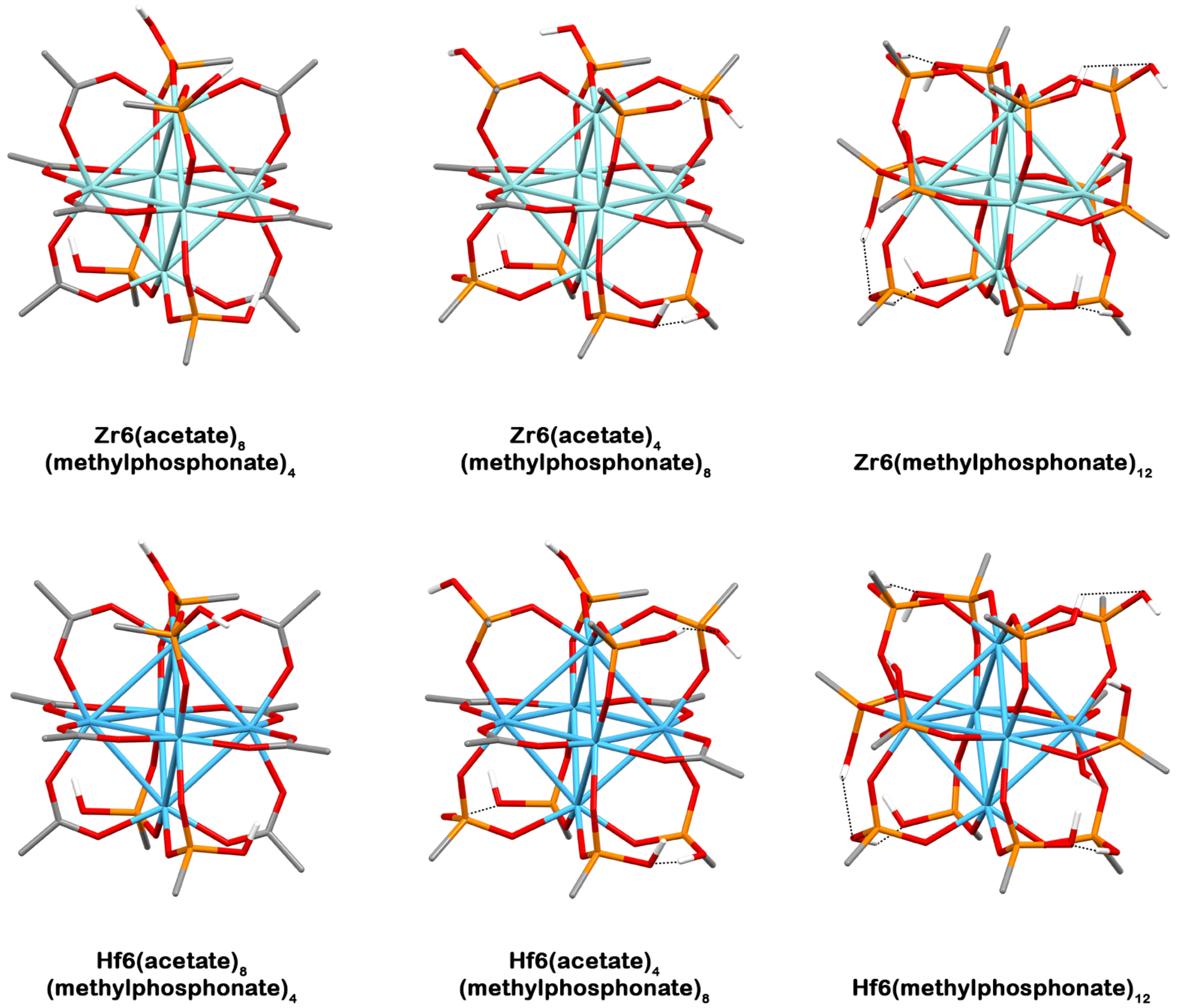

Figure S9: DFT optimized structures of Zr/Hf acetate clusters partially or fully exchanged with methylphosphonic acid. Cyan and blue atoms represent zirconium and hafnium, respectively; all other atoms follow conventional CPK coloring. The dotted lines indicate some hydrogen bonds formed due to the second acidic group. Hydrogen atoms (except P-OH) and core oxygen atoms are omitted for clarity.

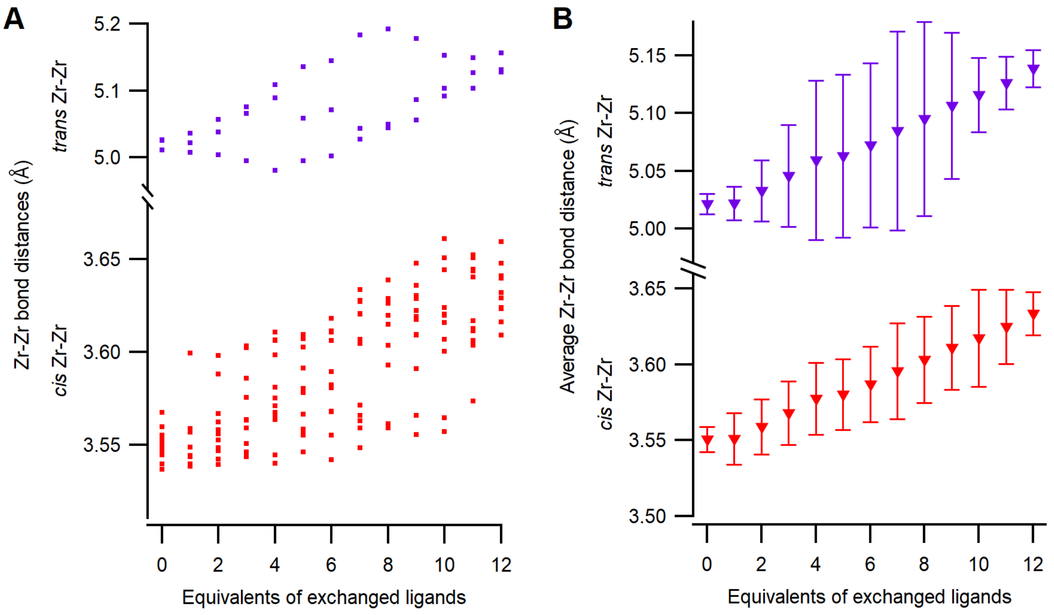

Figure S10: (A) Both *cis* and *trans* Zr-Zr distances as a function of the equivalents of exchanged methylphosphinate ligands obtained from DFT calculations. The averaged distances with standard deviation are shown in B.

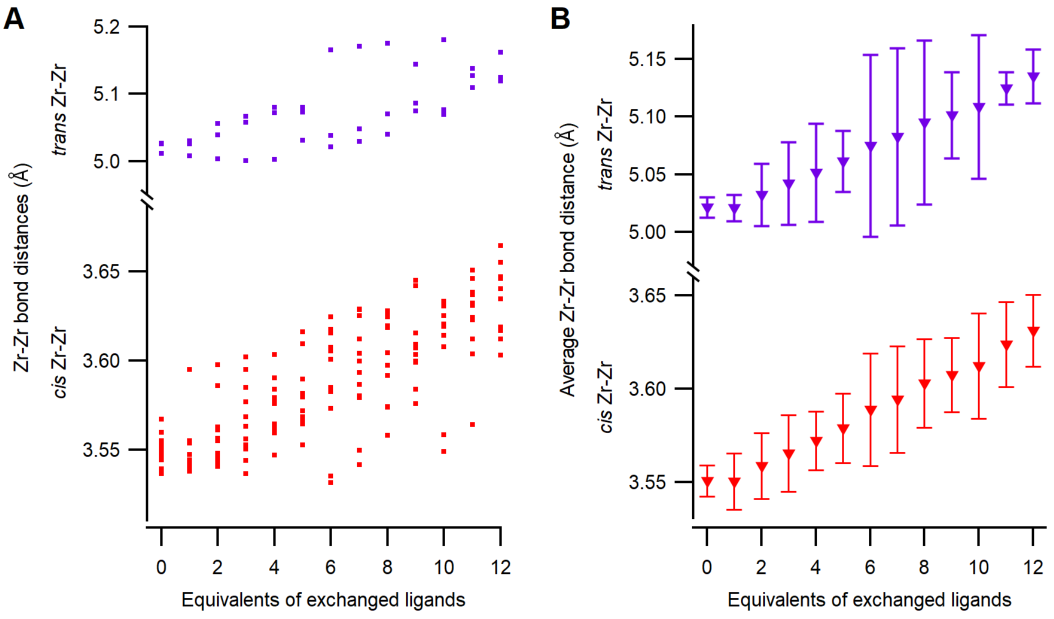

Figure S11: (A) Both *cis* and *trans* Zr-Zr distances as a function of the equivalents of exchanged ethylphosphinate ligands obtained from DFT calculations. The averaged distances with standard deviation are shown in B.

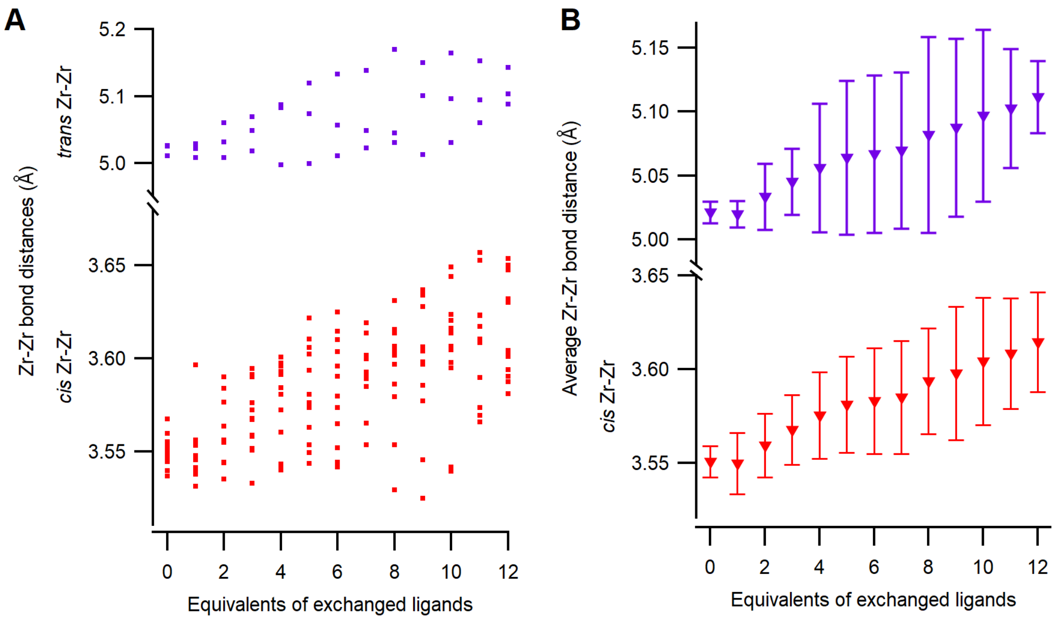

Figure S12: (A) Both *cis* and *trans* Zr-Zr distances as a function of the equivalents of exchanged methylphosphonate ligands obtained from DFT calculations. The averaged distances with standard deviation are shown in B.

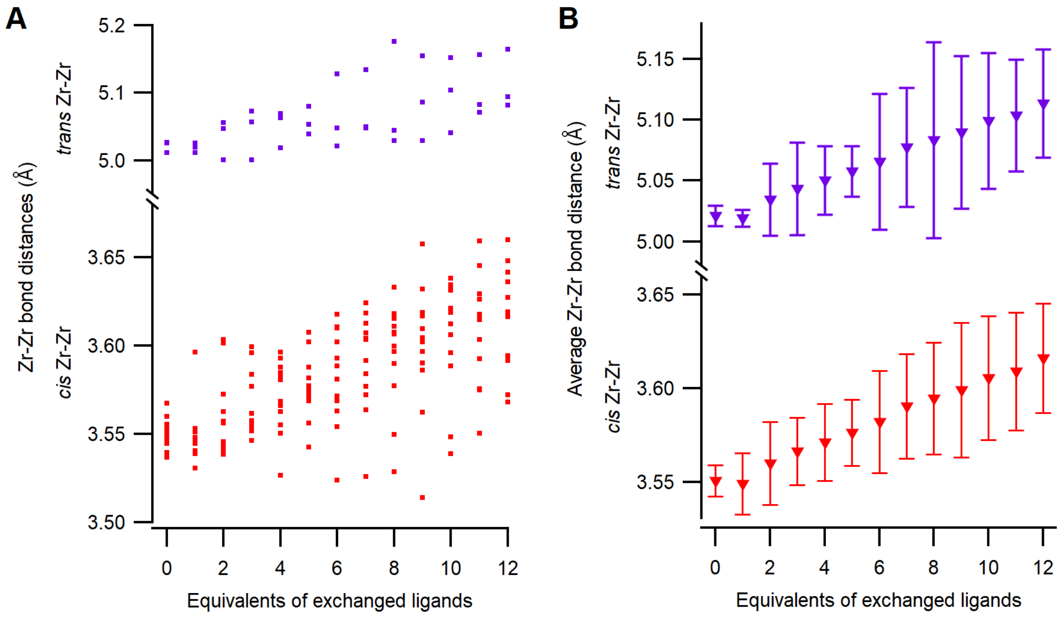

Figure S13: (A) Both *cis* and *trans* Zr-Zr distances as a function of the equivalents of exchanged ethylphosphonate ligands obtained from DFT calculations. The averaged distances with standard deviation are shown in B.

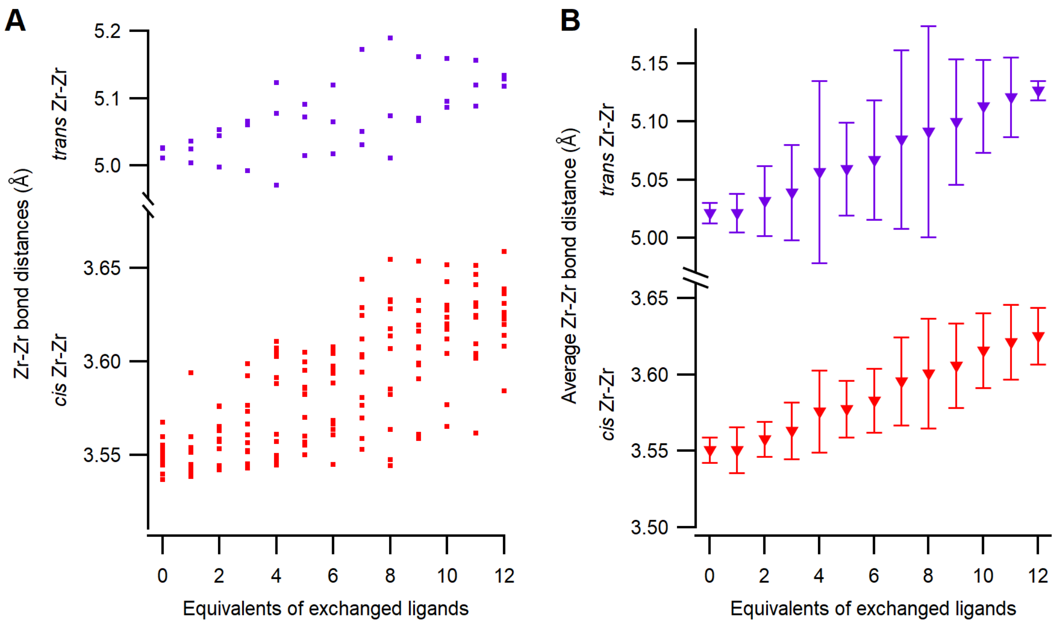

Figure S14: (A) Both *cis* and *trans* Zr-Zr distances as a function of the equivalents of exchanged dimethylphosphinate ligands obtained from DFT calculations. The averaged distances with standard deviation are shown in B.

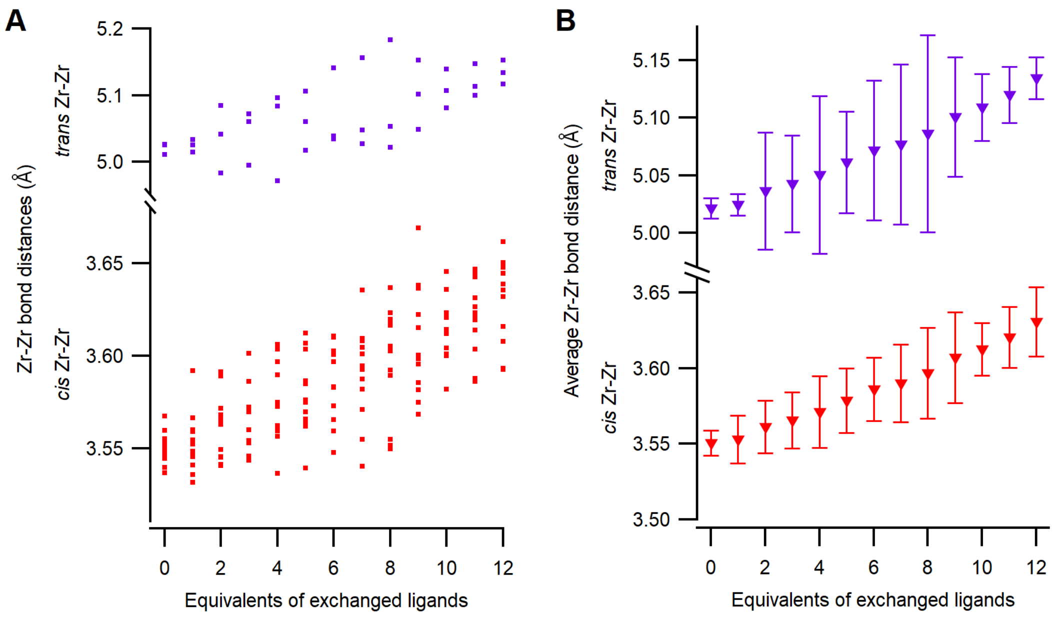

Figure S15: (A) Both *cis* and *trans* Zr-Zr distances as a function of the equivalents of exchanged diethylphosphinate ligands obtained from DFT calculations. The averaged distances with standard deviation are shown in B.

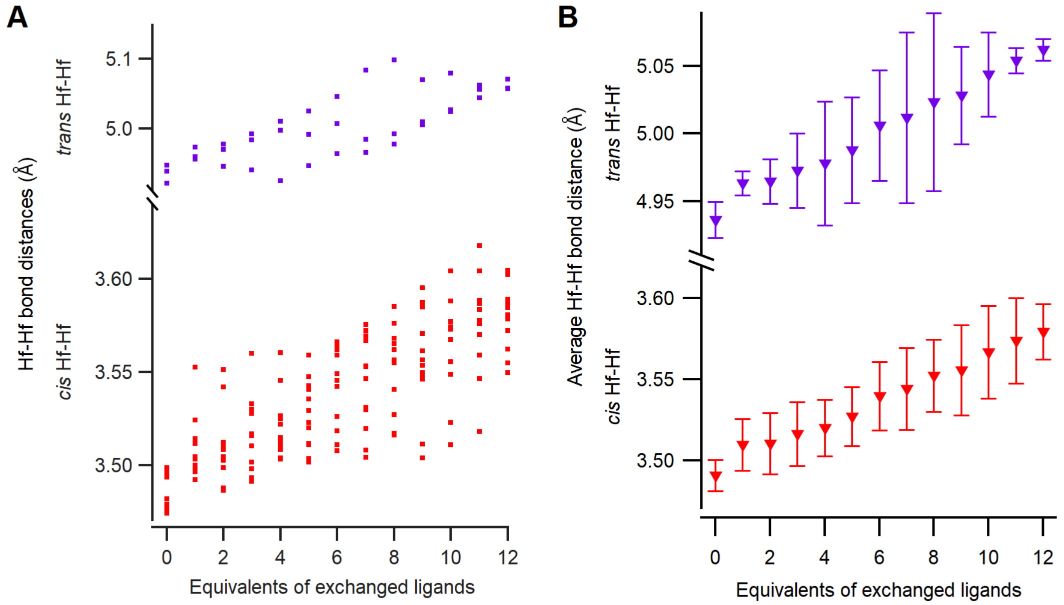

Figure S16: (A) Both *cis* and *trans* Hf-Hf distances as a function of the equivalents of exchanged methylphosphinate ligands obtained from DFT calculations. The averaged distances with standard deviation are shown in B.

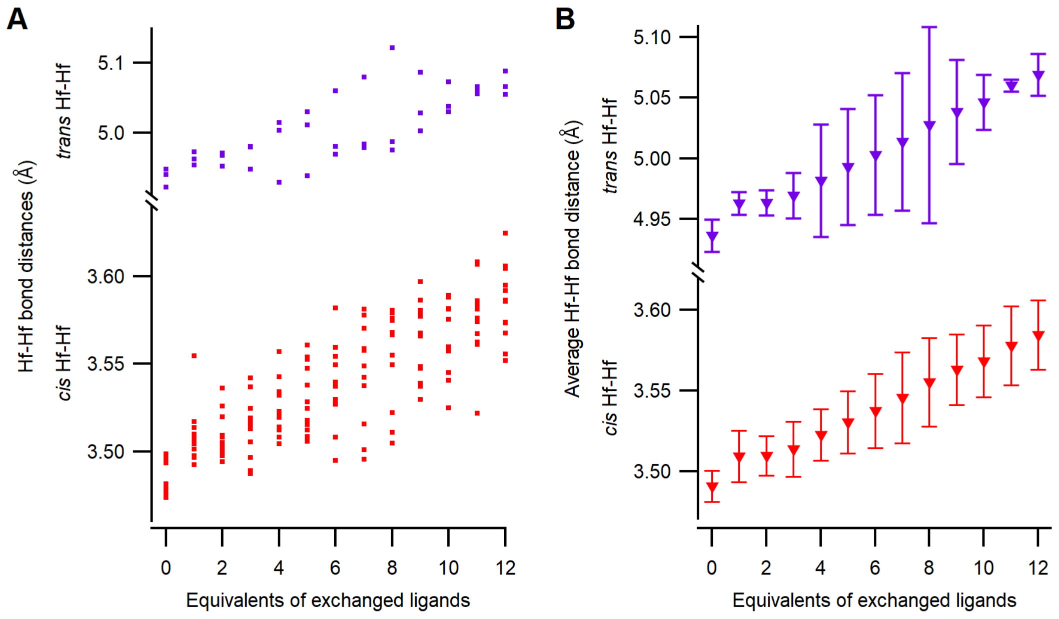

Figure S17: (A) Both *cis* and *trans* Hf-Hf distances as a function of the equivalents of exchanged ethylphosphinate ligands obtained from DFT calculations. The averaged distances with standard deviation are shown in B.

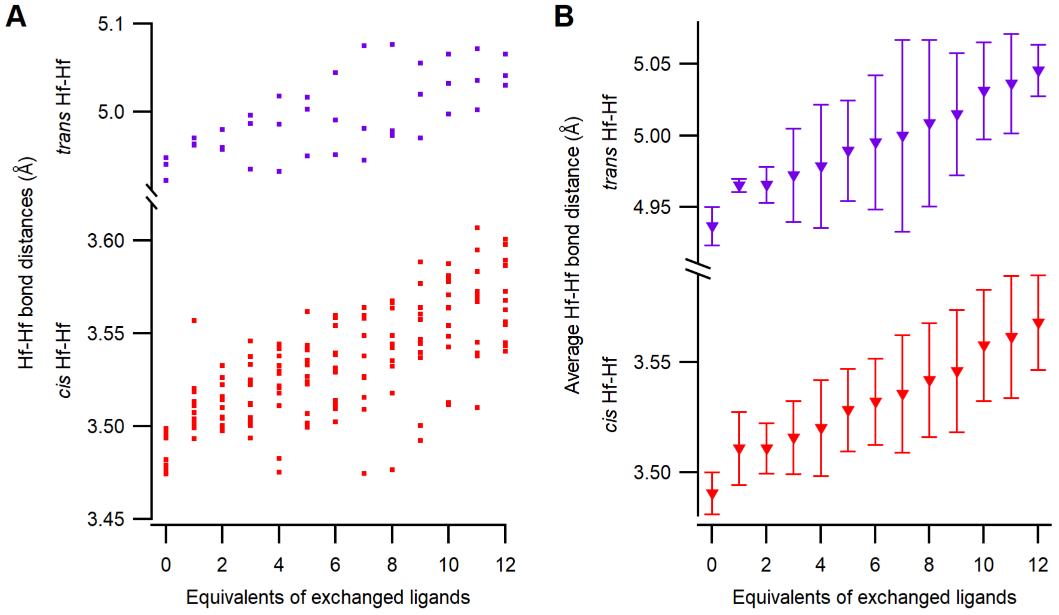

Figure S18: (A) Both *cis* and *trans* Hf-Hf distances as a function of the equivalents of exchanged methylphosphonate ligands obtained from DFT calculations. The averaged distances with standard deviation are shown in B.

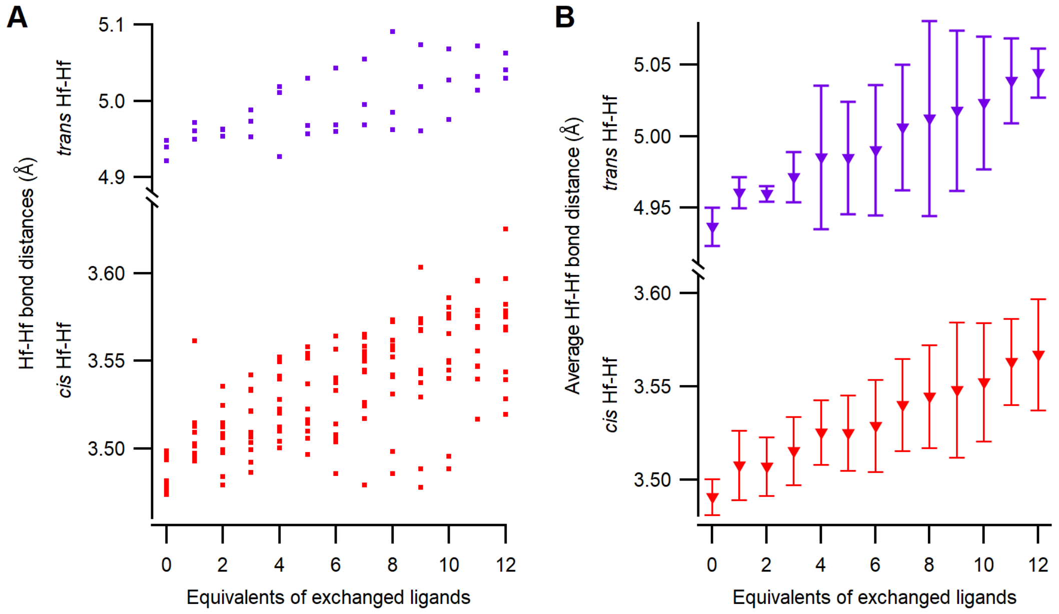

Figure S19: (A) Both *cis* and *trans* Hf-Hf distances as a function of the equivalents of exchanged ethylphosphonate ligands obtained from DFT calculations. The averaged distances with standard deviation are shown in B.

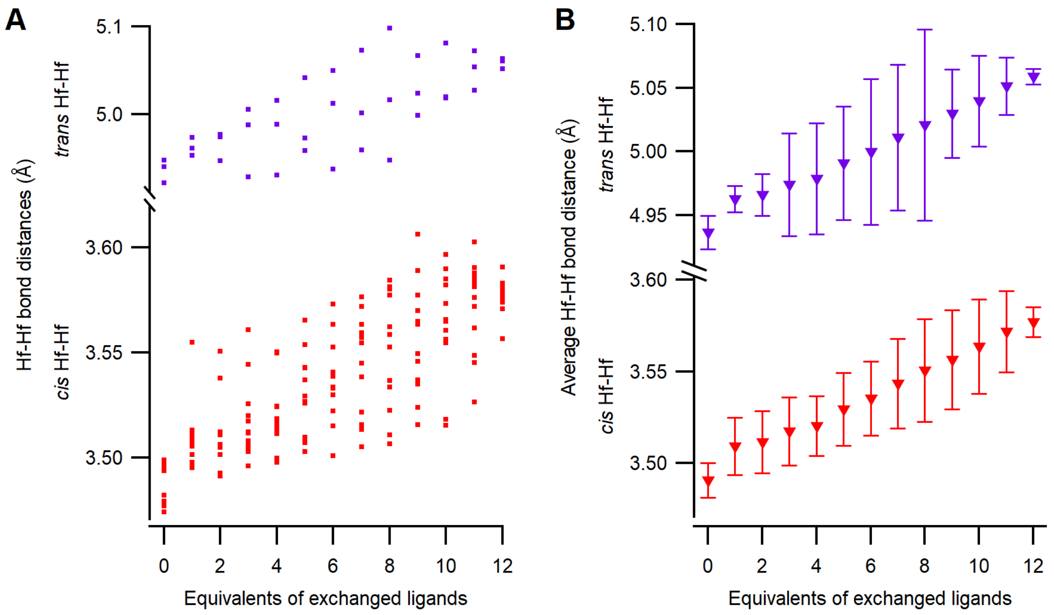

Figure S20: (A) Both *cis* and *trans* Hf-Hf distances as a function of the equivalents of exchanged dimethylphosphinate ligands obtained from DFT calculations. The averaged distances with standard deviation are shown in B.

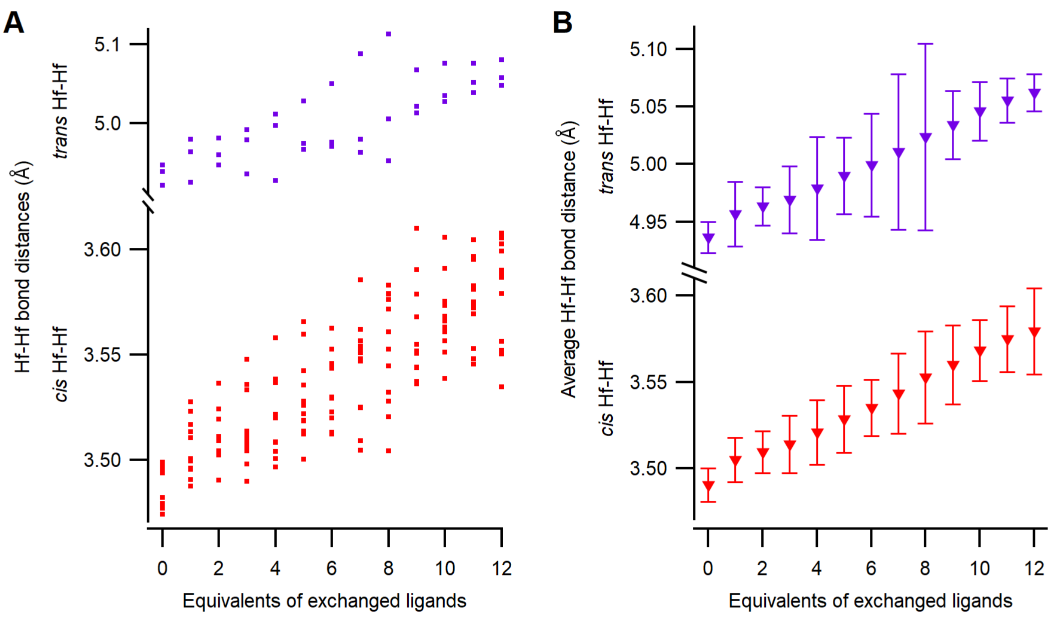

Figure S21: (A) Both *cis* and *trans* Hf-Hf distances as a function of the equivalents of exchanged diethylphosphinate ligands obtained from DFT calculations. The averaged distances with standard deviation are shown in B.

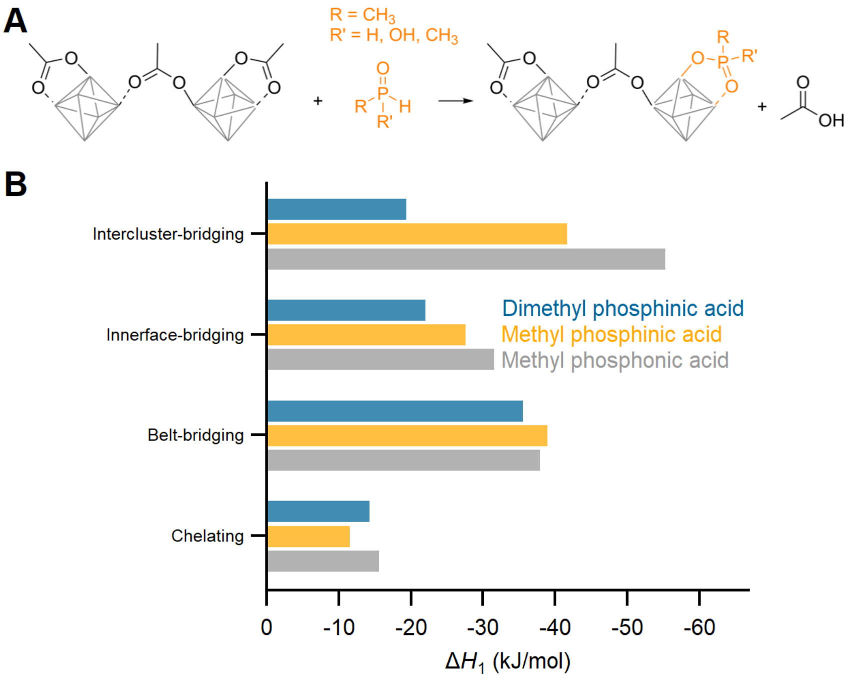

Figure S22: (A) Scheme representing the exchange of one equivalent of phosphonic acid or mono or dialkyl phosphinic acid with acetate ligand on a **Hf12** cluster. Exchanged acetate can be chelating, belt-bridging, innerface-bridging, or intercluster-bridging. Enthalpy of ligand exchange reactions on **Hf12** (B) Enthalpy of ligand exchange reactions at different binding sites.

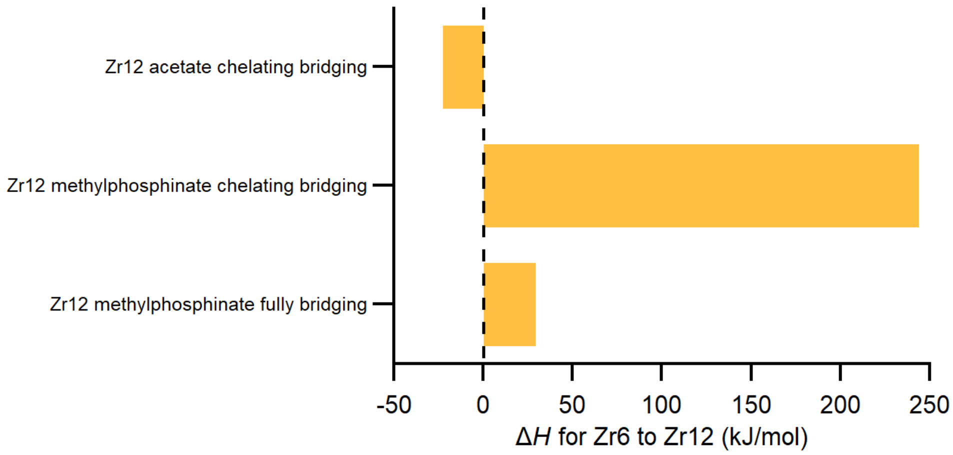

Figure S23: Enthalpy of dimerisation of fully bridged **Zr6** carboxylate or phosphinate clusters to their corresponding **Zr12**. The ligand binding modes in the **Zr12** is also indicated.

2 Ligand exchange for dialkylphosphinic acid

2.1 NMR titrations of clusters

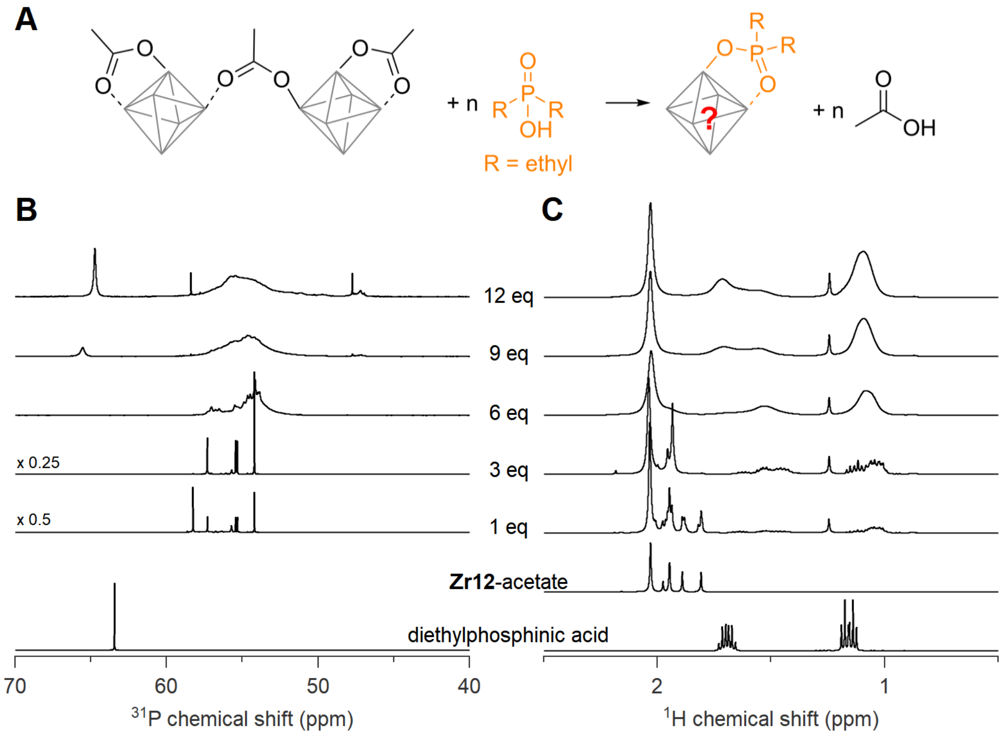

Figure S24: (A) Scheme for the titration of **Zr12**-acetate cluster with diethylphosphinic acid. (B) ^{31}P and (C) ^{1}H NMR of the titration of **Zr12**-acetate cluster with increasing equivalents of diethylphosphinic acid (expressed as equivalents with respect to a monomer unit). The cluster concentration is 20 mg/mL in $CDCl_3$. ^{31}P and ^{1}H NMR of free phosphinic acid are also provided (with one equivalent acetic acid added for the ^{31}P NMR reference).

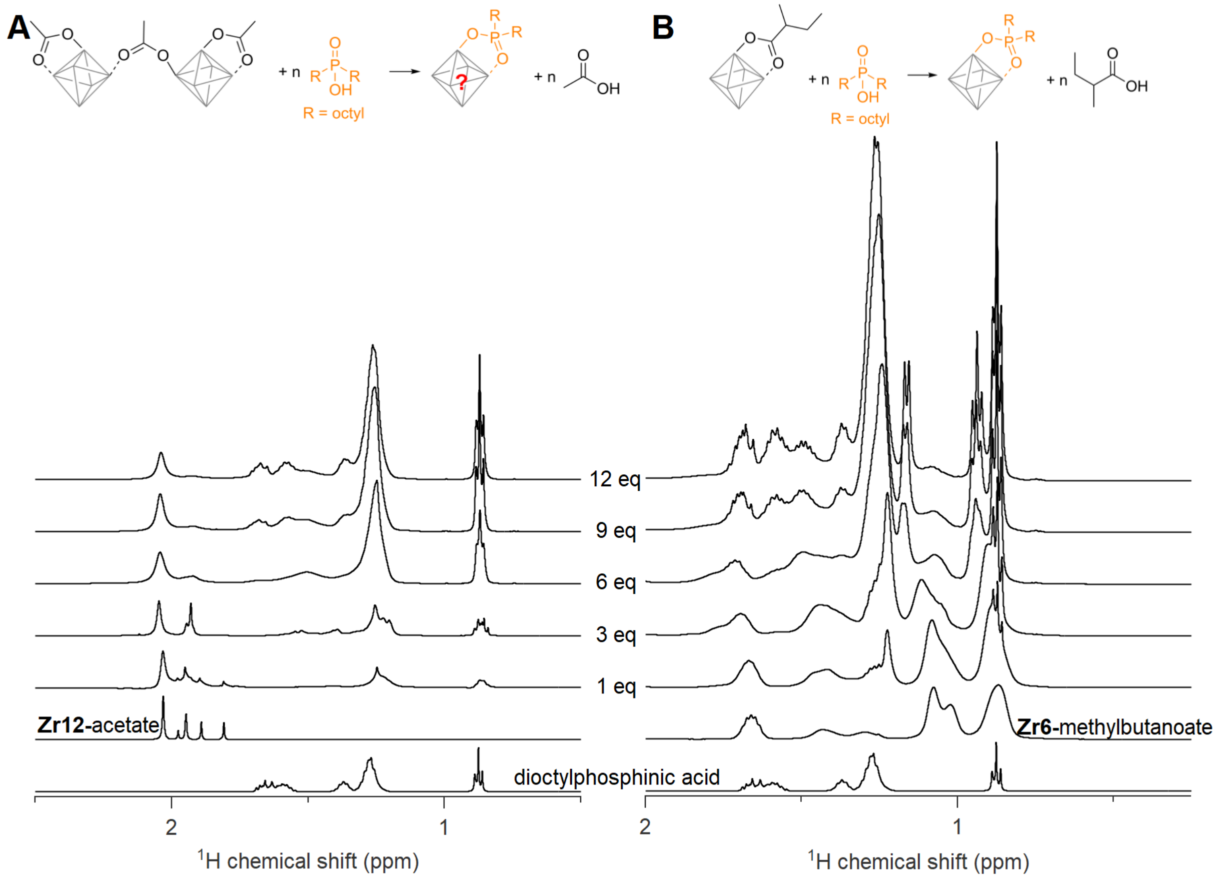

Figure S25: ^{1}H NMR spectra of the titrations of (A) **Zr12**-acetate and (B) **Zr6**-methylbutanoate with dioctylphosphinic acid (expressed as equivalents with respect to a monomer unit). The cluster concentration is 20 mg/mL in $CDCl_3$. The reference ^{1}H NMR spectrum of dioctylphosphinic acid and carboxylate clusters are also provided.

2.2 Reference spectra

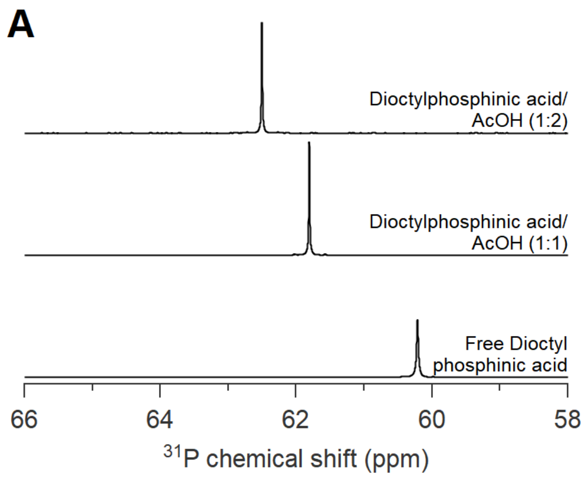

Figure S26: ^{31}P NMR of (A) dioctylphosphinic acid and (B) diethylphosphinic acid with increasing equivalents of acetic acid in $CDCl_3$. The more the acetic acid, the more deshielded the phosphorus signal.

3 Ligand exchange for aryl or alkyl phosphinic acids

3.1 NMR titrations of clusters

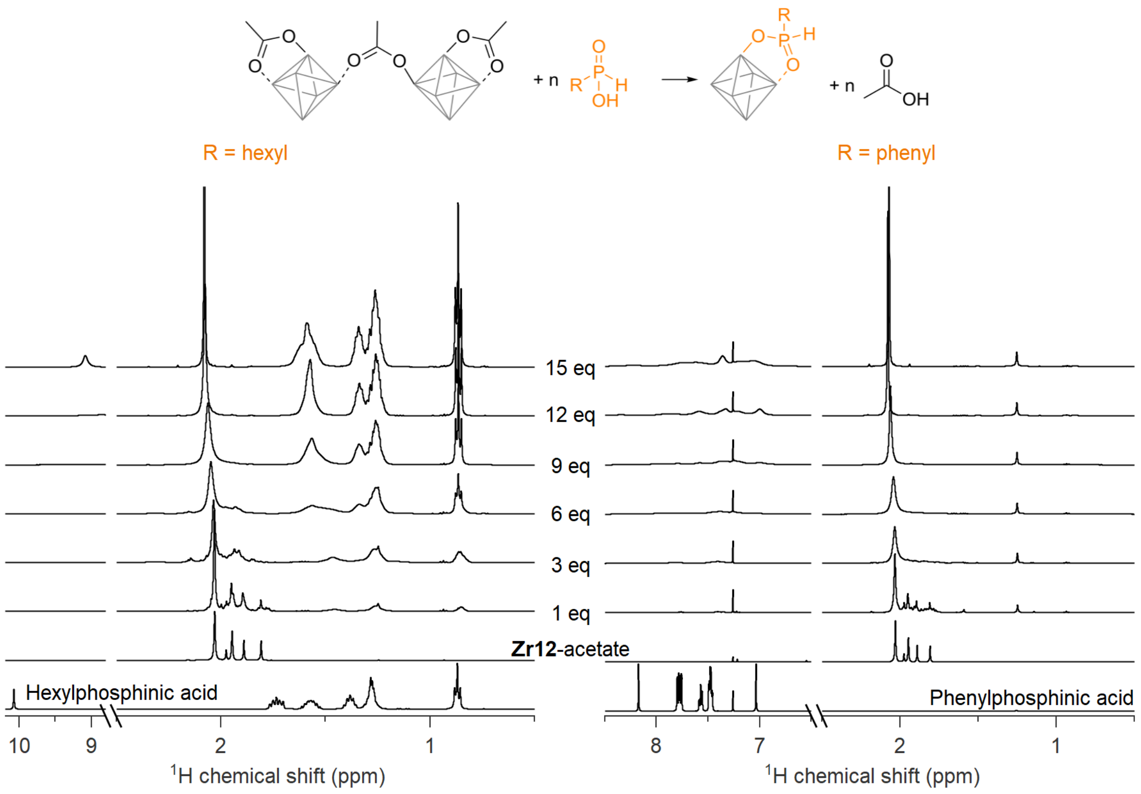

Figure S27: ^{1}H NMR spectra of the titrations of **Zr12**-acetate with phenyl and hexyl phosphinic acid (expressed as equivalents with respect to a monomer unit). The cluster concentration is 20 mg/mL in $CDCl_3$. ^{1}H NMR spectra of the free phosphinic acids and acetate cluster are provided as reference.

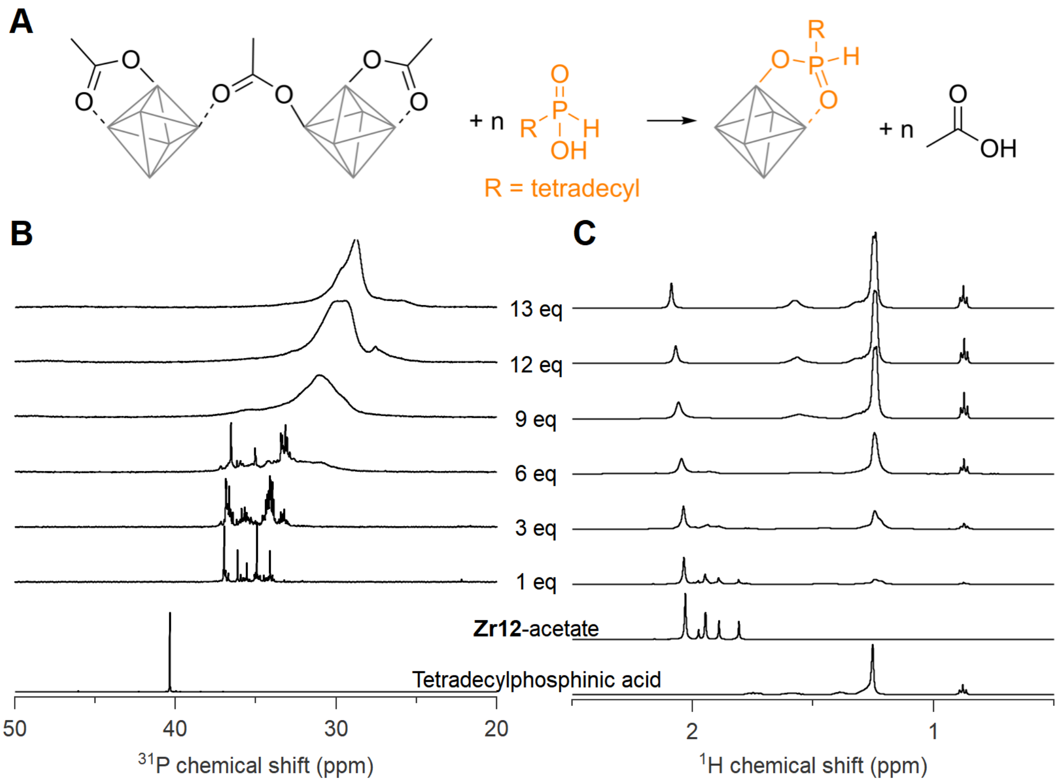

Figure S28: (A) Scheme for the titration of **Zr12**-acetate cluster with tetradecylphosphinic acid. ^{31}P (B) and ^{1}H (C) NMR of the titration of **Zr12**-acetate cluster with increasing equivalents of tetradecylphosphinic acid (expressed as equivalents with respect to a monomer unit). The cluster concentration is 20 mg/mL in $CDCl_3$. ^{31}P and ^{1}H NMR of tetradecylphosphinic acid and carboxylate clusters are also provided (with one equivalent acetic acid added for the ^{31}P NMR reference).

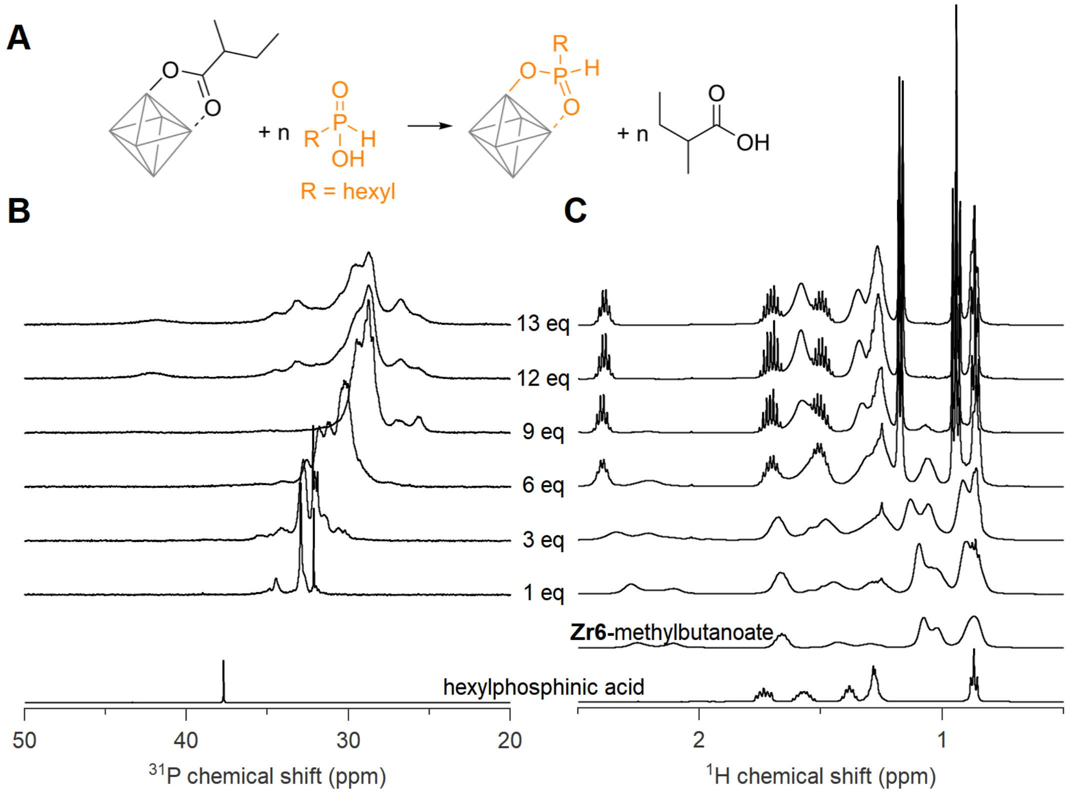

Figure S29: (A) Scheme for the titration of **Zr6**-methylbutanoate cluster with hexylphosphinic acid. ^{31}P (B) and ^{1}H (C) NMR of the titration of **Zr6**-methylbutanoate cluster with increasing equivalents of hexylphosphinic acid. The cluster concentration is 20 mg/mL in $CDCl_3$. The appearance of free 2-methylbutanoic acid confirms the ligand exchange. ^{31}P and ^{1}H NMR of hexylphosphinic acid and carboxylate clusters are also provided (with one equivalent acetic acid added for the ^{31}P NMR reference).

3.2 Reference spectra

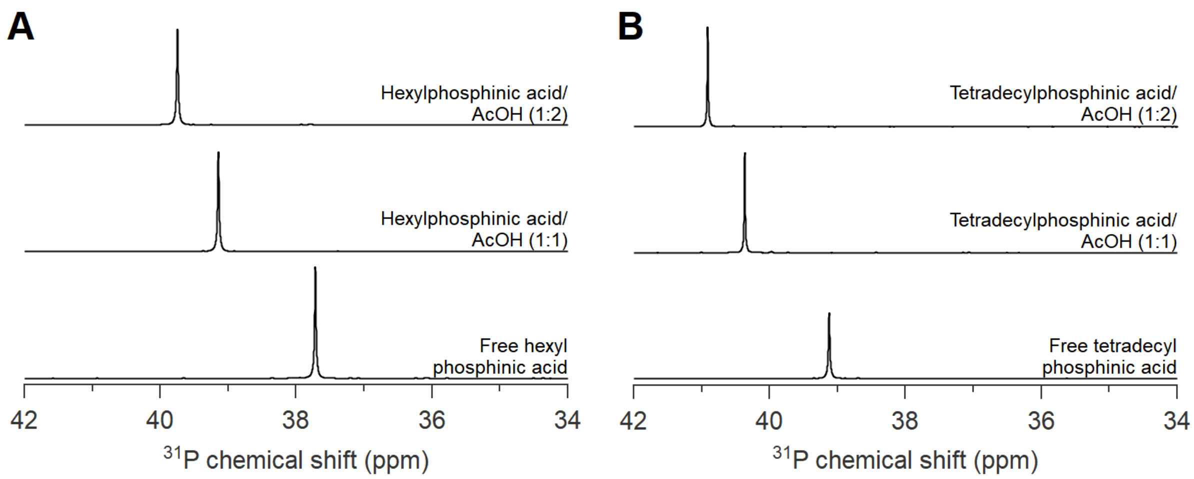

Figure S30: ^{31}P NMR of (A) hexylphosphinic acid and (B) tetradecylphosphinic acid with increasing equivalents of acetic acid in $CDCl_3$. The more the acetic acid, the more deshielded the phosphorus signal.

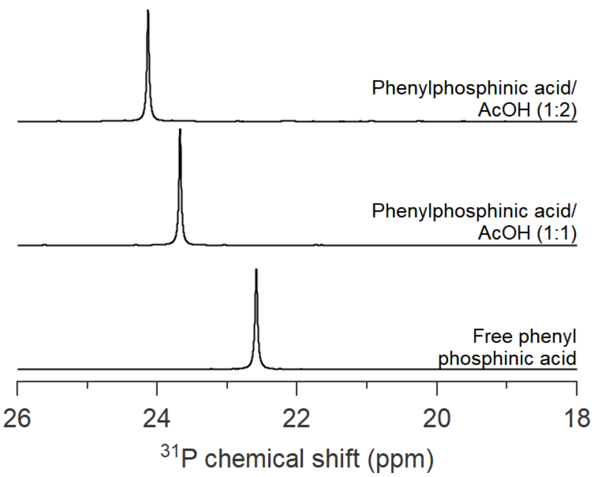

Figure S31: ^{31}P NMR of phenylphosphinic acid with increasing equivalents of acetic acid in $CDCl_3$. The more the acetic acid, the more deshielded the phosphorus signal.

3.3 Characterization of purified clusters

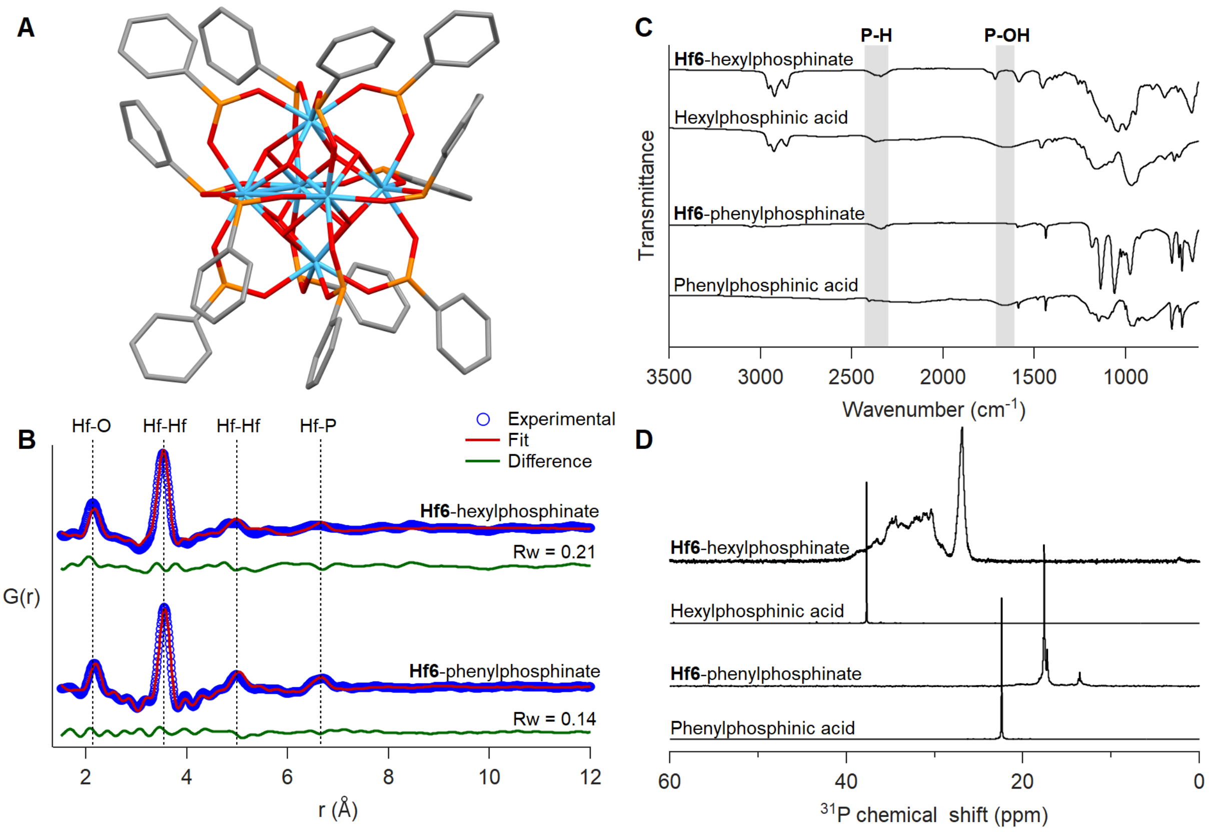

Figure S32: (A) Crystal structure of **Hf6**-phenylphosphinate cluster - $Hf_6O_4(OH)_4(OOPHPh)_{12}$. Blue atoms represent hafnium and all other atoms follow conventional CPK coloring. The co-crystallized dichloromethane molecules and hydrogen atoms are omitted for clarity. (B) PDF fit for **Hf6**-phenylphosphinate cluster with its crystal structure. PDF fit of **Hf6**-hexylphosphinate cluster with distorted **Hf6**-phosphinate cluster is also shown. (C) FTIR spectra of **Hf6**-phosphinate clusters. IR spectra of free ligands are also provided for reference. (D) ^{31}P NMR of purified **Hf6**-phosphinate clusters. ^{31}P NMR of free acids are provided as reference.

3.4 Single crystal data

Table S1: Crystallographic data and structure refinement parameters for the **Zr6** and **Hf6** phenylphosphinate clusters.

Structure	**Zr6**-phenylphosphinate	**Hf6**-phenylphosphinate
CCDC	2358676	2358675
Empirical formula	$C_{73}H_{78}Cl_2O_{32}P_{12}Zr_6$	$C_{73.5}H_{78.9}Cl_{2.9}O_{32}P_{12}Hf_6$
Formula weight	2445.11	3019.05
Temperature/K	150	150
Crystal system	triclinic	triclinic
Space group	P-1	P-1
a/Å	14.4003 (3)	14.3943 (3)
b/Å	15.2252 (4)	15.1871 (3)
c/Å	24.9863 (6)	24.9430 (5)
$\alpha/^\circ$	89.897 (2)	89.932 (2)
$\beta/^\circ$	80.487 (2)	99.740 (2)
$\gamma/^\circ$	64.685 (2)	115.133 (2)
Volume/Å^3	4869.4(2)	4849.75(19)
Z	2	2
ρ_{calc}/gcm^{-3}	1.668	2.067
μ/mm^{-1}	5.401	9.992
F(000)	2428.0	2874.0
Crystal size/mm^3	0.22 × 0.197 × 0.17	0.18 × 0.173 × 0.16
Radiation	GaKα (λ = 1.34143)	GaKα (λ = 1.34143)
2Θ range for data collection/°	6.008 to 111.626	8.336 to 111.808
Index ranges	$-13 \leq h \leq 17$, $-18 \leq k \leq 18$, $-28 \leq l \leq 30$	$-15 \leq h \leq 17$, $-18 \leq k \leq 10$, $-30 \leq l \leq 30$
Reflections collected	91695	69963
Independent reflections	18798 [R_{int} = 0.0713, R_{sigma} = 0.0416]	18736 [R_{int} = 0.0541, R_{sigma} = 0.0306]
Data/restraints/parameters	18798/333/1127	18736/793/976
Goodness-of-fit on F^2	1.048	1.046
Final R indexes [I>= 2σ(I)]	R_1 = 0.0875, wR_2 = 0.2465	R_1 = 0.0899, wR_2 = 0.2276
Final R indexes [all data]	R_1 = 0.0976, wR_2 = 0.2591	R_1 = 0.0984, wR_2 = 0.2348
Largest diff. peak/hole/eÅ^{-3}	1.99/-1.77	3.04/-1.26

Table S2: Average *cis* and *trans* M-M distances of cluster cores from crystal structures.

Cluster	Average ***cis*** M-M distance (Å)	Average ***trans*** M-M distance (Å)
**Zr6**-acetate	3.534	4.997
**Zr6**-benzoate	3.525	4.985
**Zr12**-acetate	3.509	4.962
**Hf12**-acetate	3.494	4.941
**Zr6**-dimethylphosphate	3.575	5.055
**Zr6**-phenylphosphinate	3.581	5.064
**Hf6**-phenylphosphinate	3.563	5.039

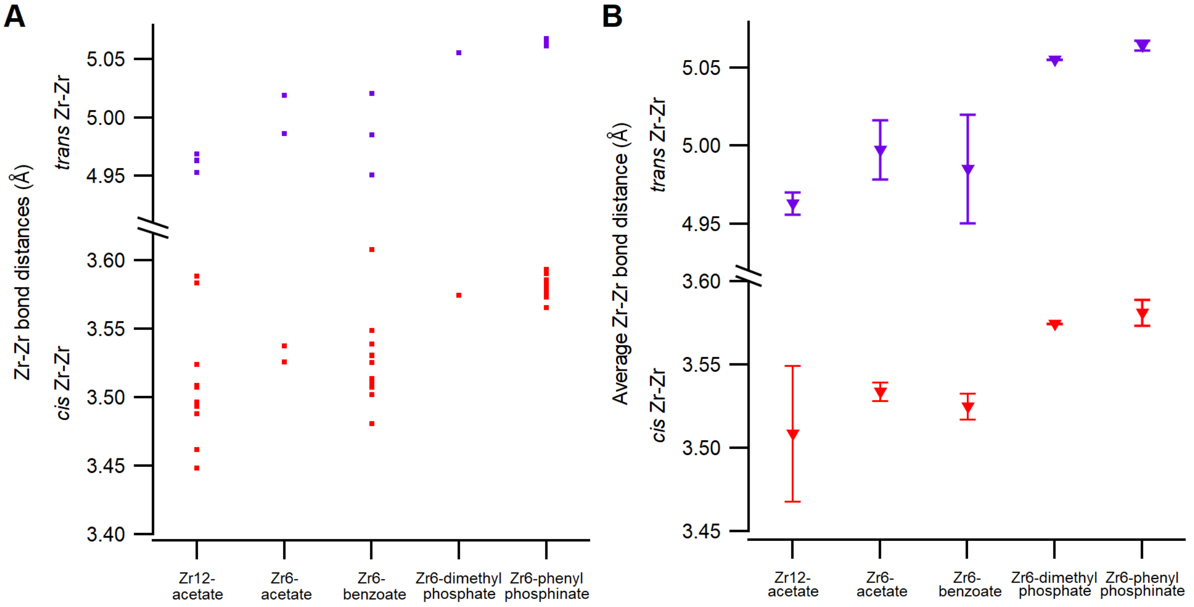

Figure S33: (A) Both *cis* and *trans* Zr-Zr distances of different zirconium crystal structures - **Zr12**-acetate (CCDC-604528),[S3] **Zr6**-acetate (CCDC-1051013),[S1] **Zr6**-benzoate (CCDC-117768),[S4] **Zr6**-dimethylphosphate (CCDC-1863035),[S5] and **Zr6**-phenylphosphinate (this work). The averaged distances with standard deviation are shown in B.

3.5 PDF refinement data

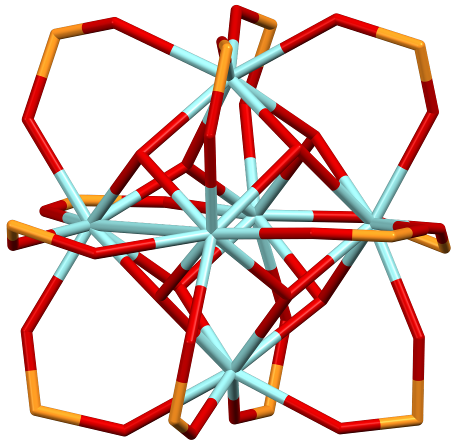

Figure S34: Structure of distorted **Zr6** phosphinate predicted from PDF refinement. Cyan atoms represent zirconium and all other atoms follow conventional CPK coloring.

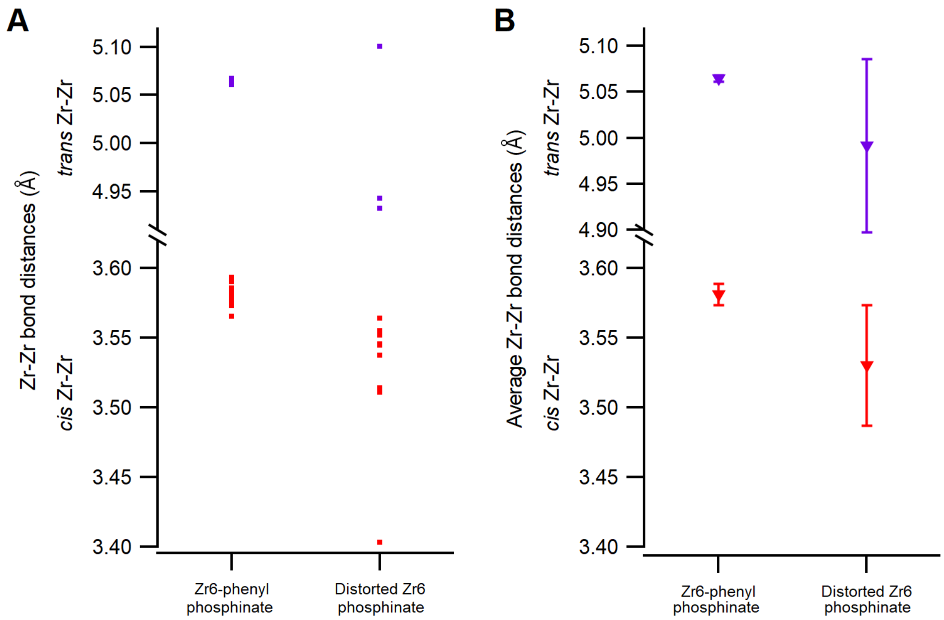

Figure S35: (A) Both *cis* and *trans* Zr-Zr distances of **Zr6**-phenylphosphinate and distorted **Zr6**-phosphinate structure. The averaged distances with standard deviation are shown in B.

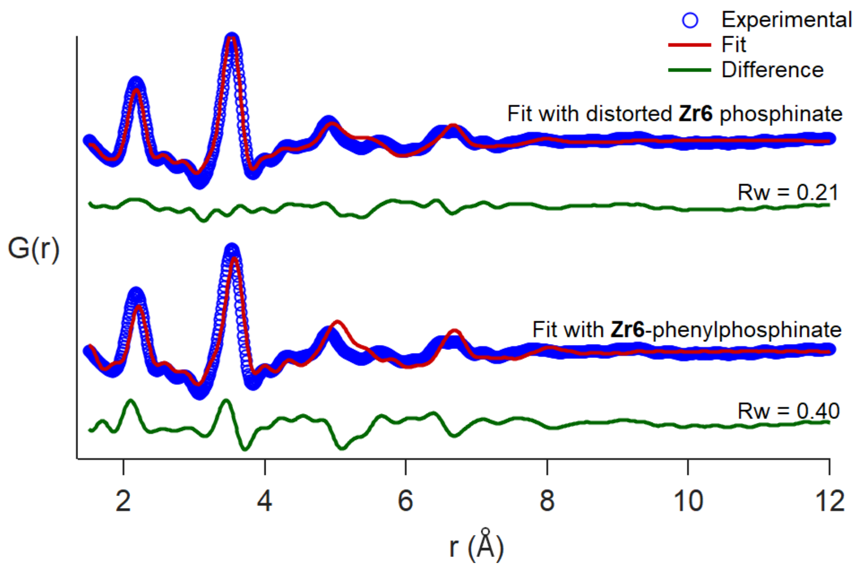

Figure S36: PDF fit for synthesized **Zr6**-hexylphosphinate cluster with the crystal structure of **Zr6**-phenylphosphinate and distorted phosphinate clusters.

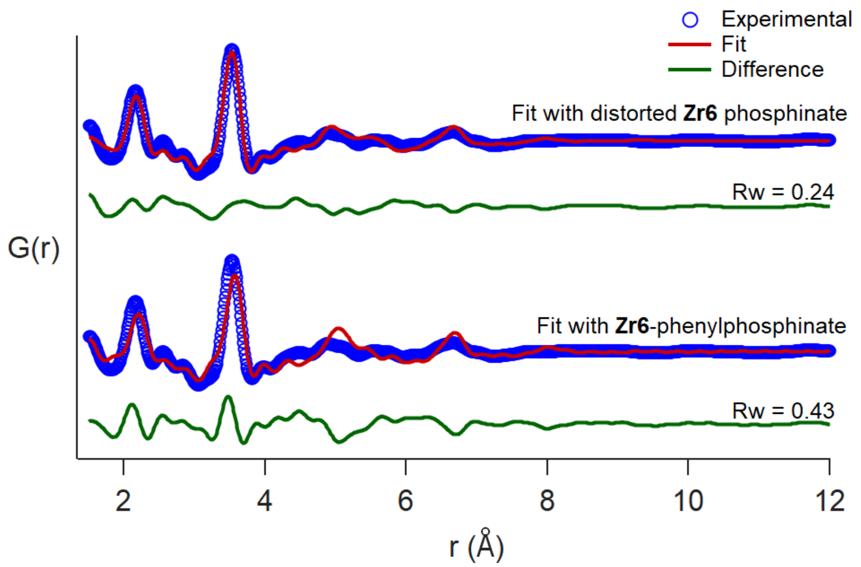

Figure S37: PDF fit for synthesized **Zr6**-tetradecylphosphinate cluster with the crystal structure of **Zr6** phenylphosphinate and distorted phosphinate clusters.

Table S3: Refined parameters after fitting monoalkylphosphinate capped **Zr6** clusters with the crystal structure of **Zr6**-phenylphosphinate and distorted phosphinate clusters.

**Experimental data**	**Zr6**-phenylphosphinate	**Zr6**-hexylphosphinate		**Zr6**-tetradecylphosphinate	
**Model**	**Zr6**-phenyl phosphinate	**Zr6**-phenyl phosphinate	distorted **Zr6** phosphinate	**Zr6**-phenyl phosphinate	distorted **Zr6** phosphinate
**scale**	0.50	0.81	0.87	0.49	0.51
**Uiso Zr**[$Å^2$]	0.003	0.004	0.005	0.003	0.003
**Uiso O**[$Å^2$]	0.010	0.013	0.012	0.015	0.010
**Uiso P**[$Å^2$]	0.009	0.008	0.010	0.008	0.010
**Uiso C**[$Å^2$]	0.020	0.030	-	0.060	-
**delta2**	2.1	2.1	2.4	2.2	2.2
Rw	0.18	0.40	0.21	0.43	0.24

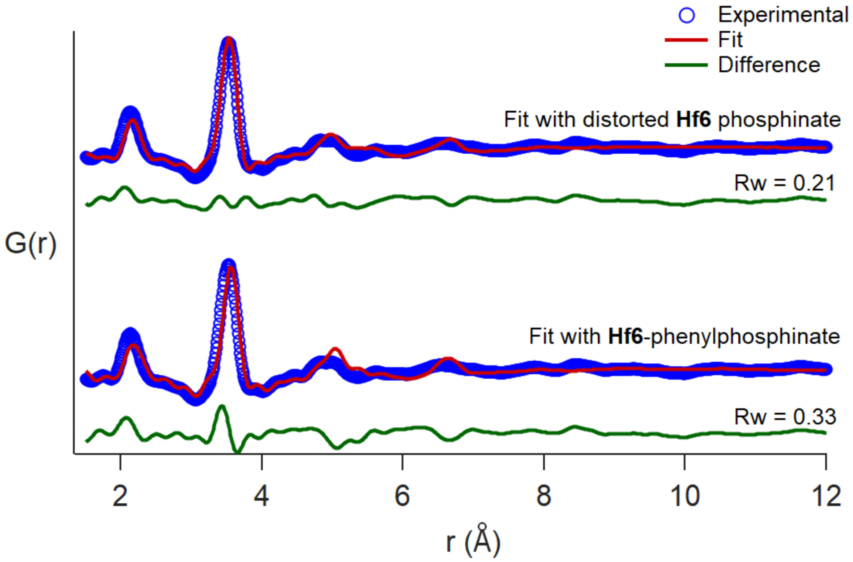

Figure S38: PDF fit for synthesized **Hf6**-hexylphosphinate cluster with the crystal structure of **Hf6**-phenylphosphinate and distorted phosphinate clusters.

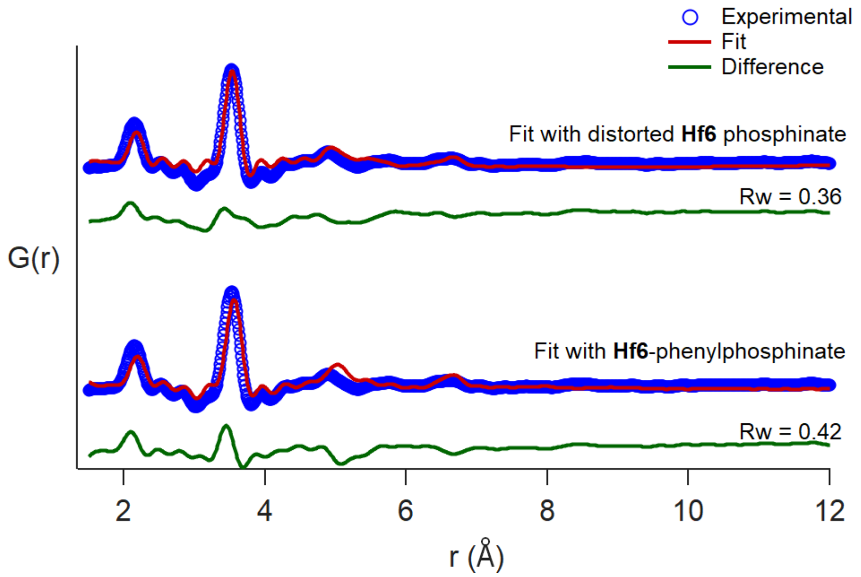

Figure S39: PDF fit for synthesized **Hf6**-tetradecylphosphinate cluster with the crystal structure of **Hf6**-phenylphosphinate and distorted phosphinate clusters.

Table S4: Refined parameters after fitting monoalkylphosphinate capped **Hf6** clusters with the crystal structure of **Hf6**-phenylphosphinate and distorted phosphinate clusters.

**Experimental data**	**Hf6**-phenylphosphinate	**Hf6**-hexylphosphinate		**Hf6**-tetradecylphosphinate	
**Model**	**Hf6**-phenyl phosphinate	**Hf6**-phenyl phosphinate	distorted **Hf6** phosphinate	**Hf6**-phenyl phosphinate	distorted **Hf6** phosphinate
**scale**	0.18	2.61	3.58	0.21	0.26
**Uiso Hf[Å^2]**	0.005	0.005	0.005	0.004	0.003
**Uiso O[Å^2]**	0.021	0.011	0.010	0.017	0.010
**Uiso P[Å^2]**	0.010	0.008	0.008	0.008	0.008
**Uiso C[Å^2]**	0.030	0.060	-	0.060	-
**delta2**	3.5	1.9	2.5	3.5	3.5
Rw	0.14	0.33	0.21	0.42	0.36

3.6 Dynamic light scattering analysis

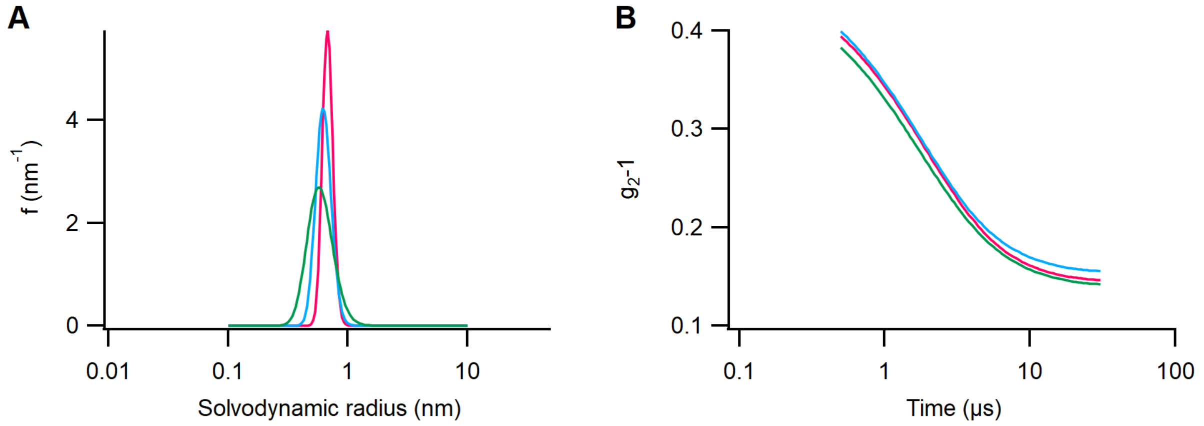

Figure S40: (A) DLS particle size distribution and (B) correlogram for measurements of 40 mg/mL solution of **Zr6**-hexylphosphinate in dichloromethane after manual fitting. Average solvodynamic radius = 0.64 ± 0.04 nm, polydispersity index = 0.03 ± 0.02. The different colors represent individual measurements taken in triplicate.

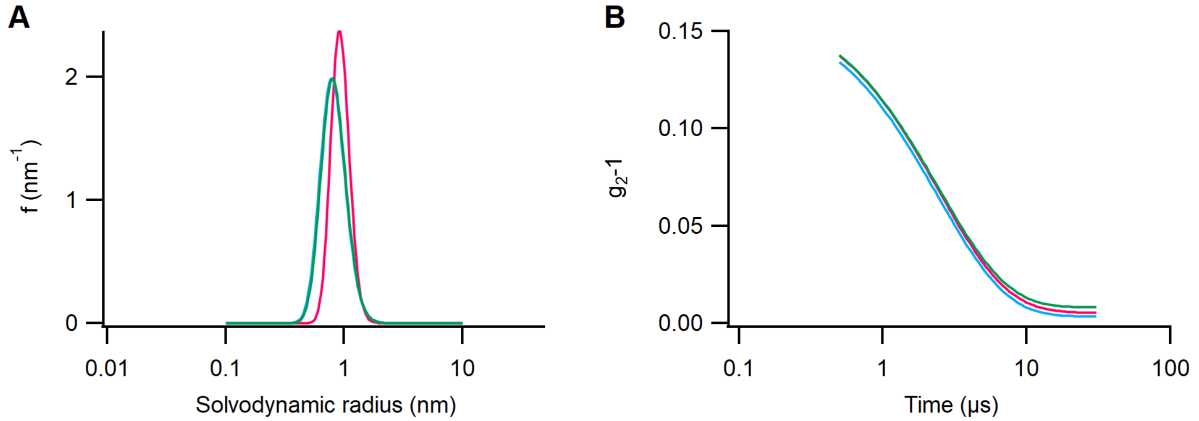

Figure S41: (A) DLS particle size distribution and (B) correlogram for measurements of 40 mg/mL solution of **Zr12**-hexanoate in dichloromethane after manual fitting. Average solvodynamic radius = 0.91 ± 0.05 nm, polydispersity index = 0.05 ± 0.02. The different colors represent individual measurements taken in triplicate.

3.7 ^{1}H and ^{31}P NMR spectra of purified clusters

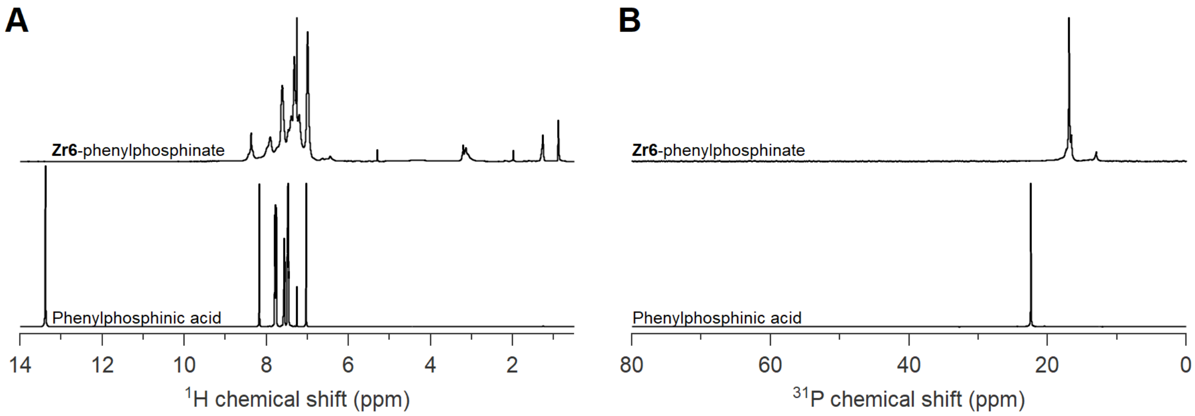

Figure S42: ^{1}H (A) and ^{31}P (B) NMR of purified **Zr6**-phenylphosphinate cluster in $CDCl_3$. ^{1}H and ^{31}P NMR of free acids are provided as reference. The broadening of NMR signals confirms the cluster binding of new phenylphosphinate ligands.

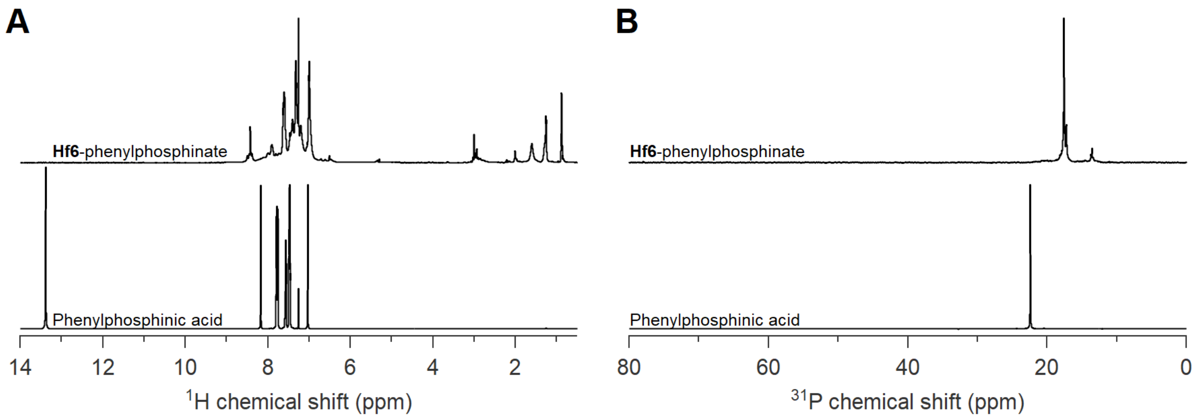

Figure S43: ^{1}H (A) and ^{31}P (B) NMR of purified **Hf6**-phenylphosphinate cluster in $CDCl_3$. ^{1}H and ^{31}P NMR of free acids are provided as reference. The broadening of NMR signals confirms the cluster binding of new phenylphosphinate ligands.

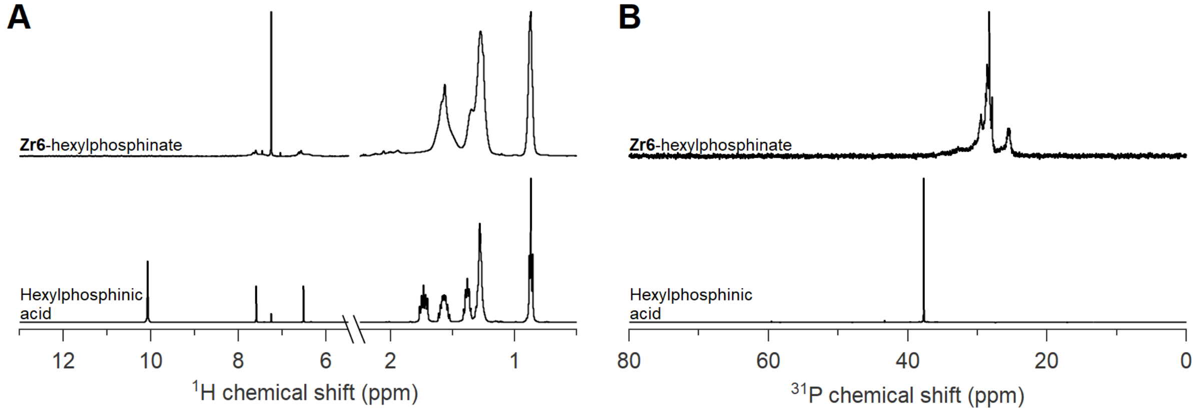

Figure S44: ^{1}H (A) and ^{31}P (B) NMR of purified **Zr6**-hexylphosphinate cluster in $CDCl_3$. ^{1}H and ^{31}P NMR of free acids are provided as reference. The broadening of NMR signals confirms the cluster binding of new hexylphosphinate ligands.

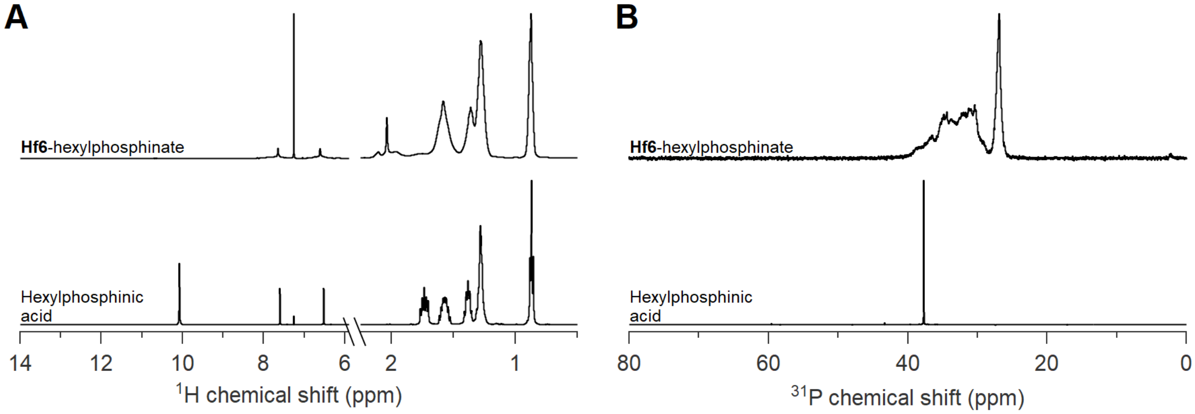

Figure S45: ^{1}H (A) and ^{31}P (B) NMR of purified **Hf6**-hexylphosphinate cluster in $CDCl_3$. ^{1}H and ^{31}P NMR of free acids are provided as reference. The broadening of NMR signals confirms the cluster binding of new hexylphosphinate ligands.

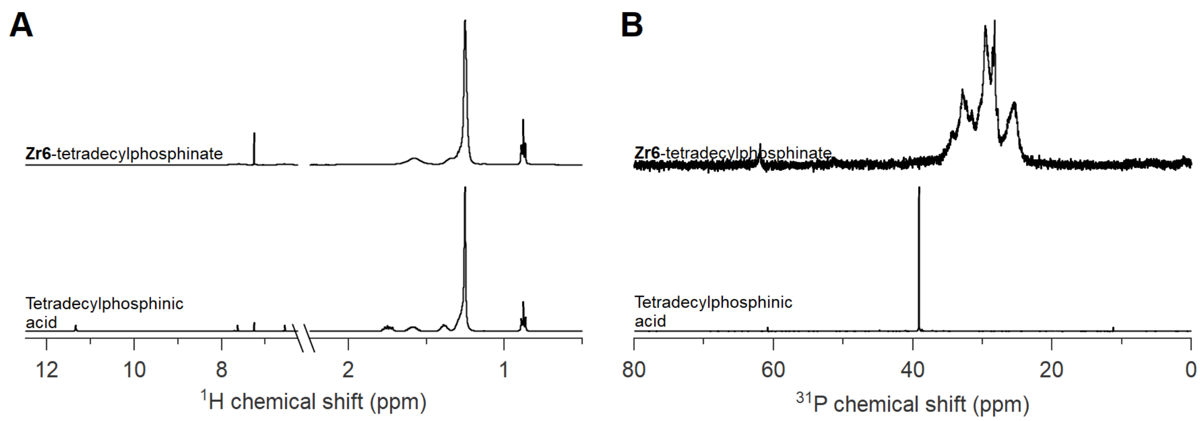

Figure S46: ^{1}H (a) and ^{31}P (b) NMR of purified **Zr6**-tetradecylphosphinate cluster in $CDCl_3$. ^{1}H and ^{31}P NMR of free acids are provided as reference. The broadening of NMR signals confirms the cluster binding of new phenylphosphinate ligands.

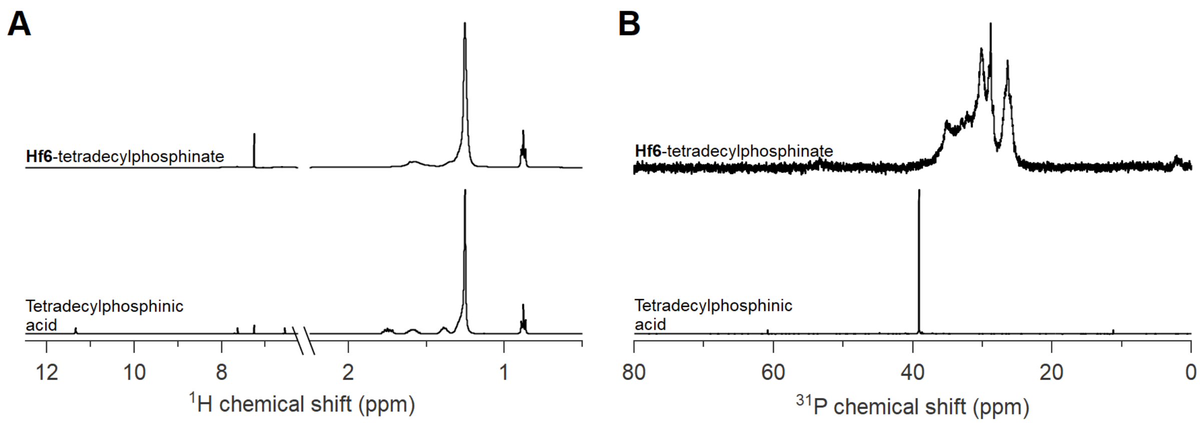

Figure S47: ^{1}H (a) and ^{31}P (b) NMR of purified **Hf6**-tetradecylphosphinate cluster in $CDCl_3$. ^{1}H and ^{31}P NMR of free acids are provided as reference. The broadening of NMR signals confirms the cluster binding of new phenylphosphinate ligands.

3.8 ESI-HRMS analysis

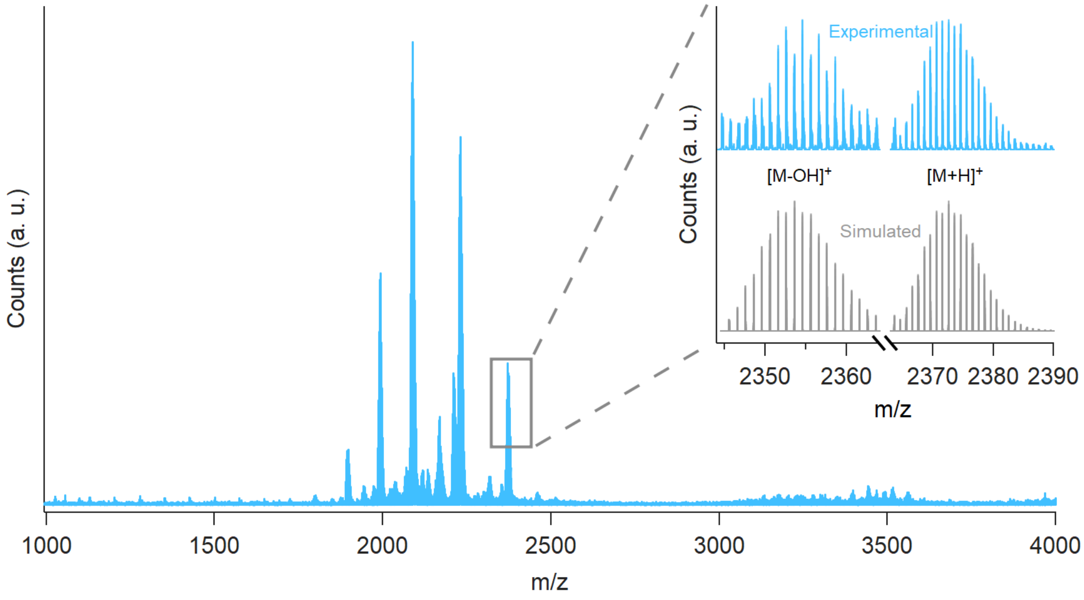

Figure S48: ESI-HRMS analysis of **Zr6**-phenylphosphinate cluster - $Zr_6O_4(OH)_4(C_6H_5PHOO)_{12}$. Both the experimental and simulated spectra are shown.

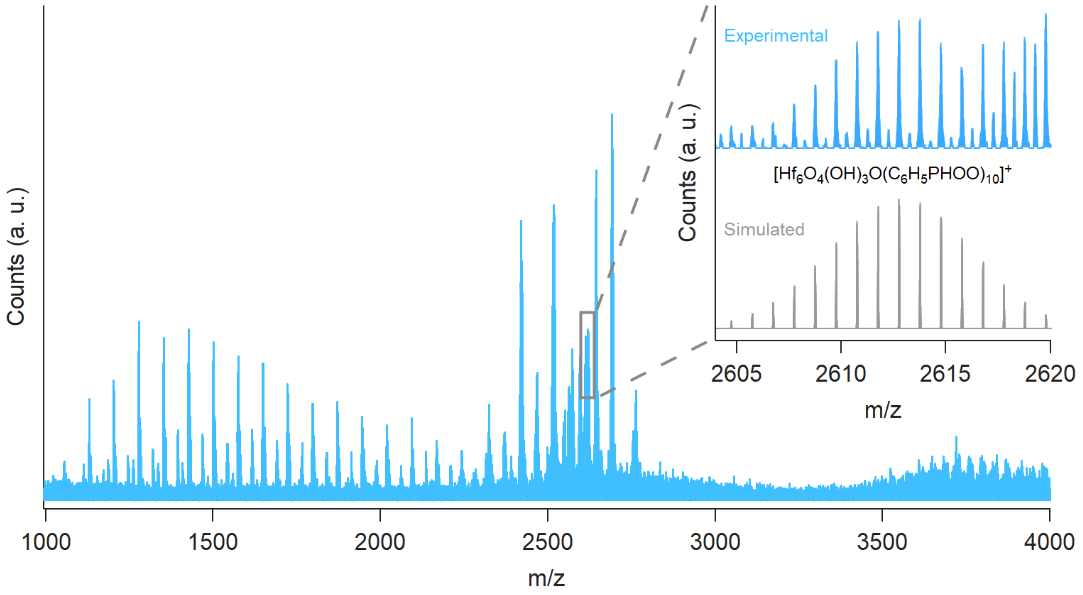

Figure S49: ESI-HRMS analysis of **Hf6**-phenylphosphinate cluster - $Hf_6O_4(OH)_4(C_6H_5PHOO)_{12}$. Both the experimental and simulated spectra are shown.

3.9 Powder X-ray diffraction data

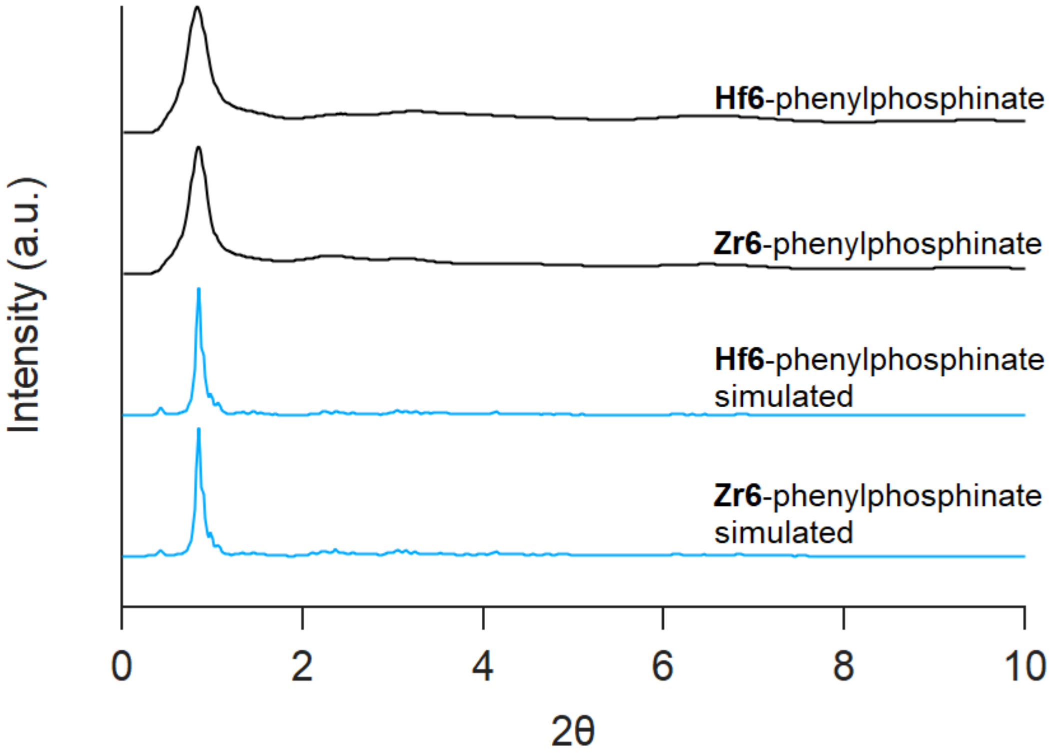

Figure S50: Powder X-ray diffraction data of **Zr6**- and **Hf6**-phenylphosphinate clusters. The simulated powder patterns (λ = 0.1821 Å, same as experimental wavelength) are provided as reference.

3.10 Ligand stripping experiments

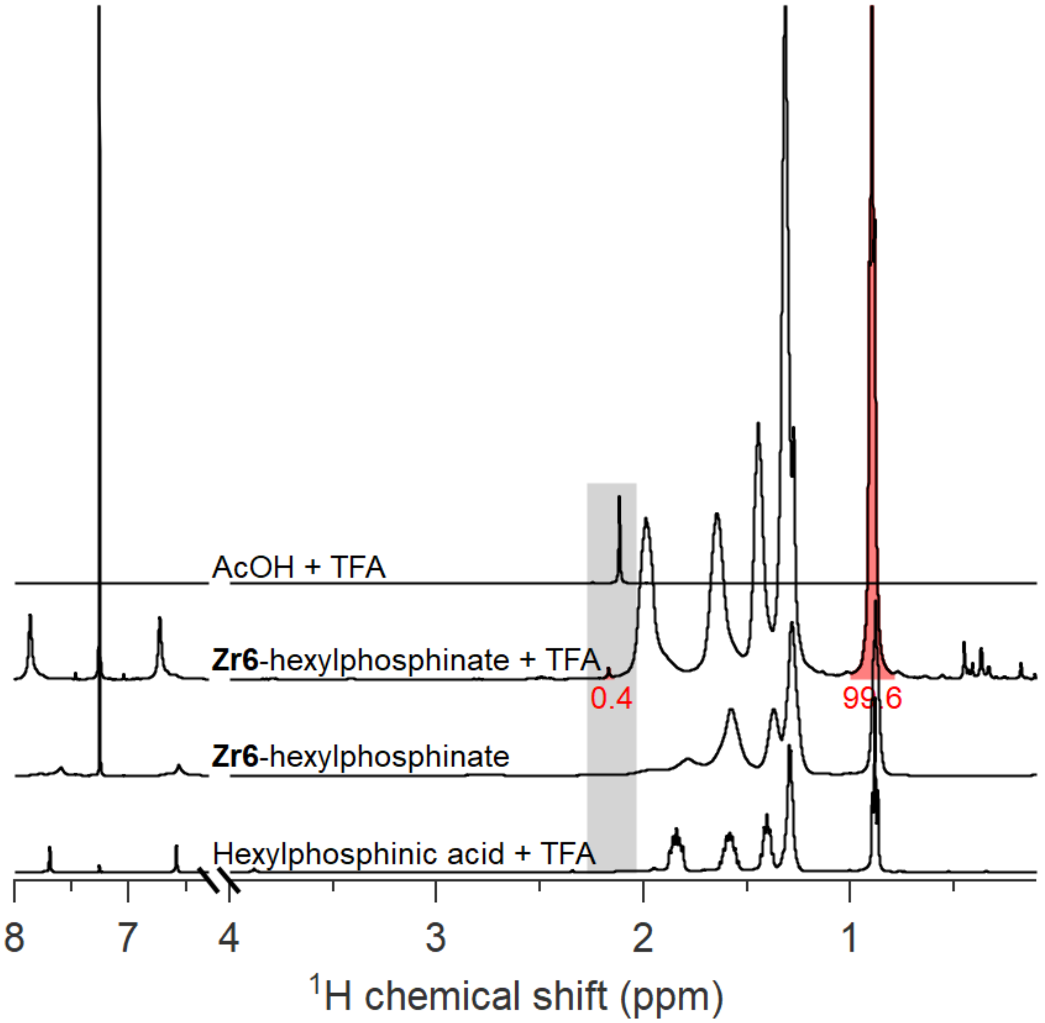

Figure S51: ^{1}H NMR of **Zr6**-hexylphosphinate cluster after ligand stripping experiments with trifluoroacetic acid. ^{1}H NMR of hexylphosphinic acid and acetic acid in trifluoroacetic acid are provided as references. The integral values corresponding to the methyl group of both ligands are also mentioned.

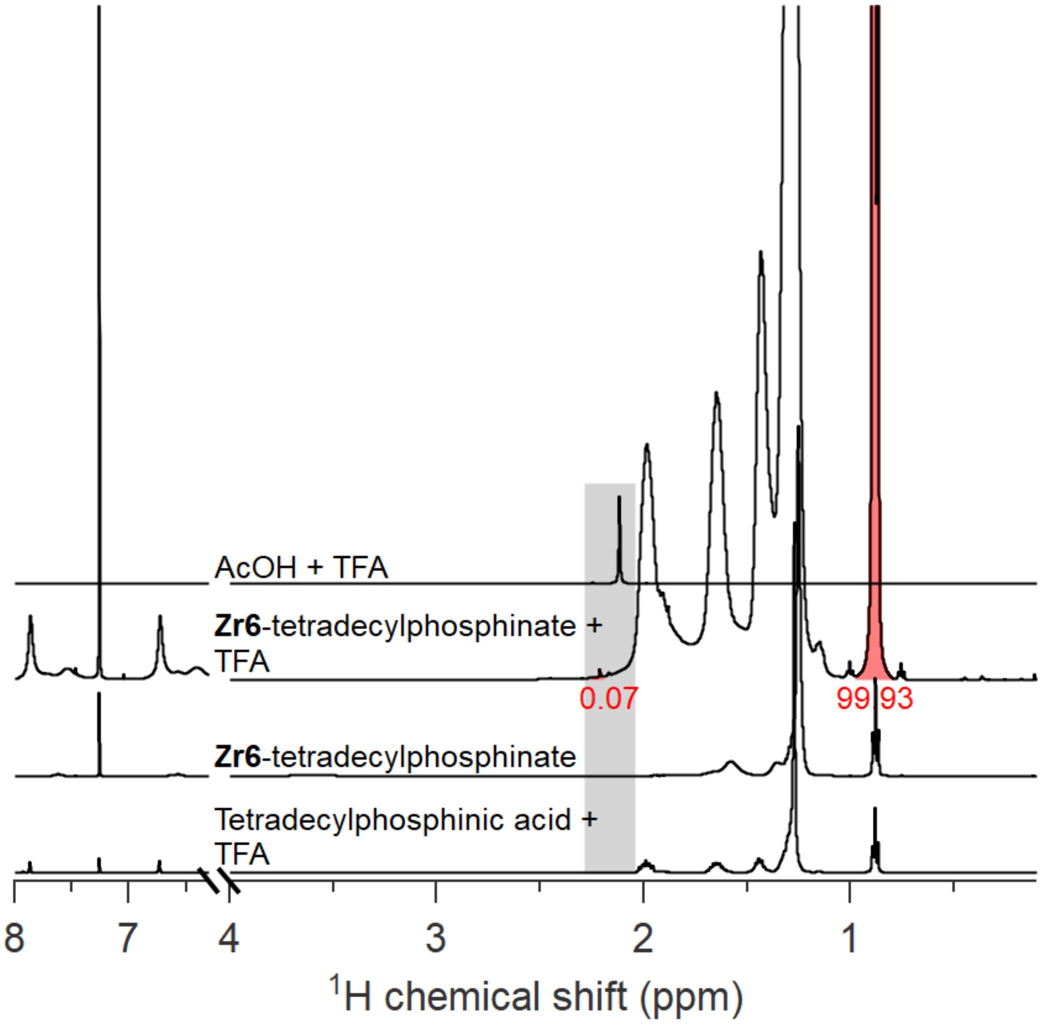

Figure S52: ^{1}H NMR of **Zr6**-tetradecylphosphinate cluster after ligand stripping experiments with trifluoroacetic acid. ^{1}H NMR of tetradecylphosphinic acid and acetic acid in trifluoroacetic acid are provided as references. The integral values corresponding to the methyl group of both ligands are also mentioned.

4 Ligand Exchange with phosphonic acids

4.1 NMR titrations of clusters

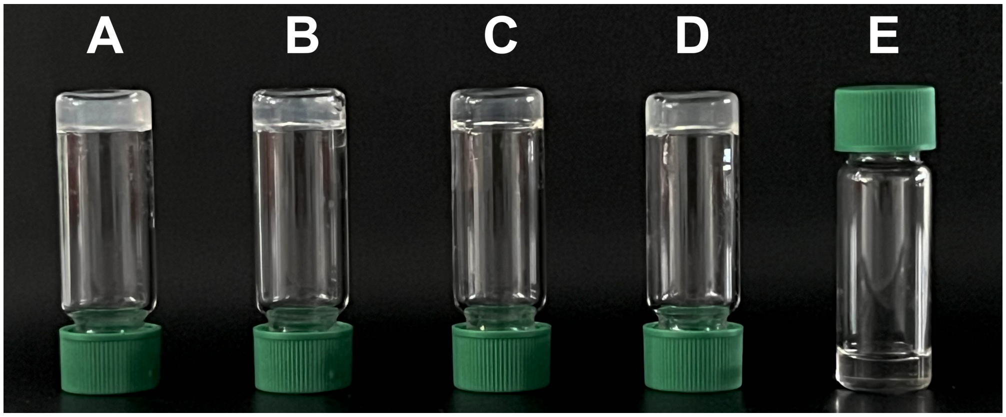

Figure S53: Gelation of **Zr12**-acetate cluster solution in $CDCl_3$ with 12 equivalents (per cluster monomer) of hexylphosphonic acid (A), dodecylphosphonic acid (B), oleylphosphonic acid (C) and 2-ethylhexylphosphonic acid (D). No gelation was observed for 2-hexyldecylphosphonic acid (E). The cluster concentration is 40 mg/mL.

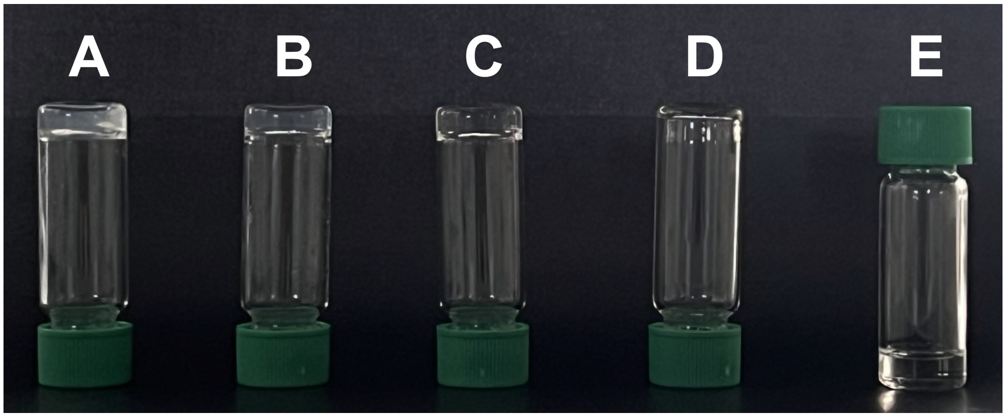

Figure S54: Gelation of **Zr6**-methylbutanoate cluster solution in $CDCl_3$ with 12 equivalents (per cluster monomer) of hexylphosphonic acid (A), dodecylphosphonic acid (B), oleylphosphonic acid (C) and 2-ethylhexylphosphonic acid (D). No gelation was observed for 2-hexyldecylphosphonic acid (E). The cluster concentration is 40 mg/mL.

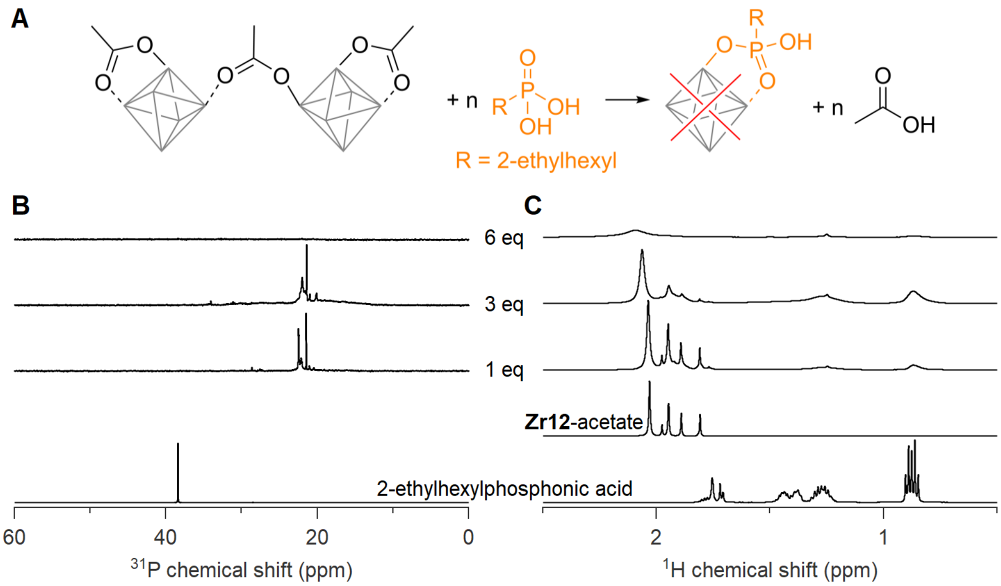

Figure S55: (A) Scheme for the titration of **Zr12**-acetate cluster with 2-ethylhexylphosphonic acid. ^{31}P (B) and ^{1}H (C) NMR of the titration of **Zr12**-acetate cluster with increasing equivalents of 2-ethylhexylphosphonic acid (expressed as equivalents with respect to a monomer unit). The cluster concentration is 20 mg/mL in $CDCl_3$. ^{31}P and ^{1}H NMR of 2-ethylhexylphosphonic acid and carboxylate clusters are also provided (with one equivalent acetic acid added for the ^{31}P NMR reference).

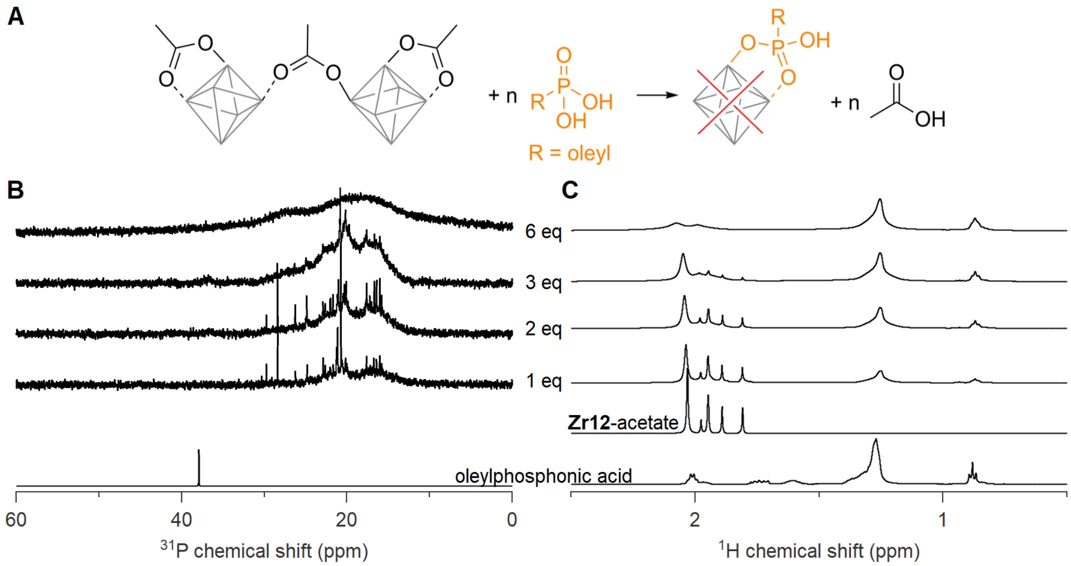

Figure S56: (A) Scheme for the titration of **Zr12**-acetate cluster with oleylphosphonic acid. ^{31}P (B) and ^{1}H (C) NMR of the titration of **Zr12**-acetate cluster with oleylphosphonic acid. Gelation prevented the data acquisition at high equivalents. ^{31}P and ^{1}H NMR of oleyl phosphonic acid and carboxylate clusters are also provided (with one equivalent acetic acid added for the ^{31}P NMR reference).

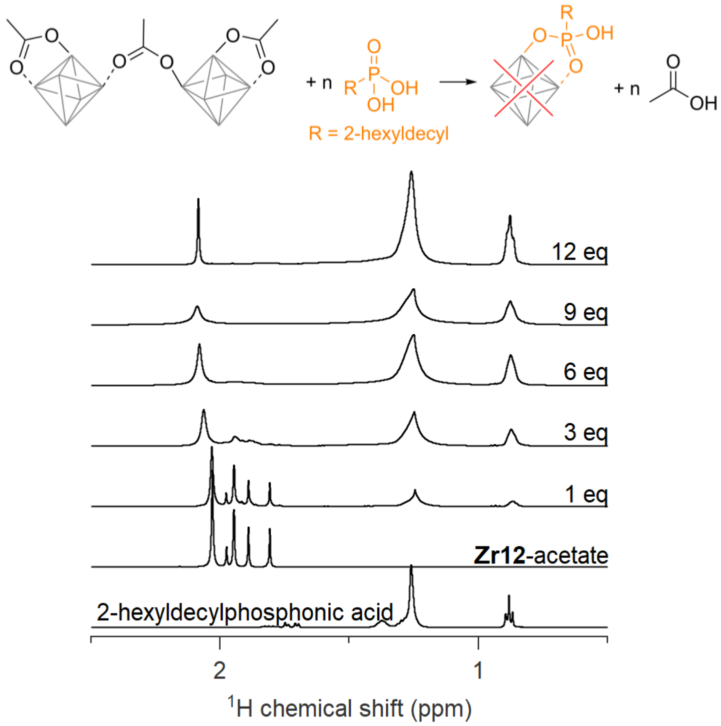

Figure S57: ^{1}H NMR of the titration of a solution of **Zr12**-acetate cluster with increasing 2-hexyldecylphosphonic acid (expressed as equivalents with respect to a monomer unit). The cluster concentration is 20 mg/mL in $CDCl_3$. ^{1}H NMR of 2-hexyldecylphosphonic acid and acetate clusters are also provided.

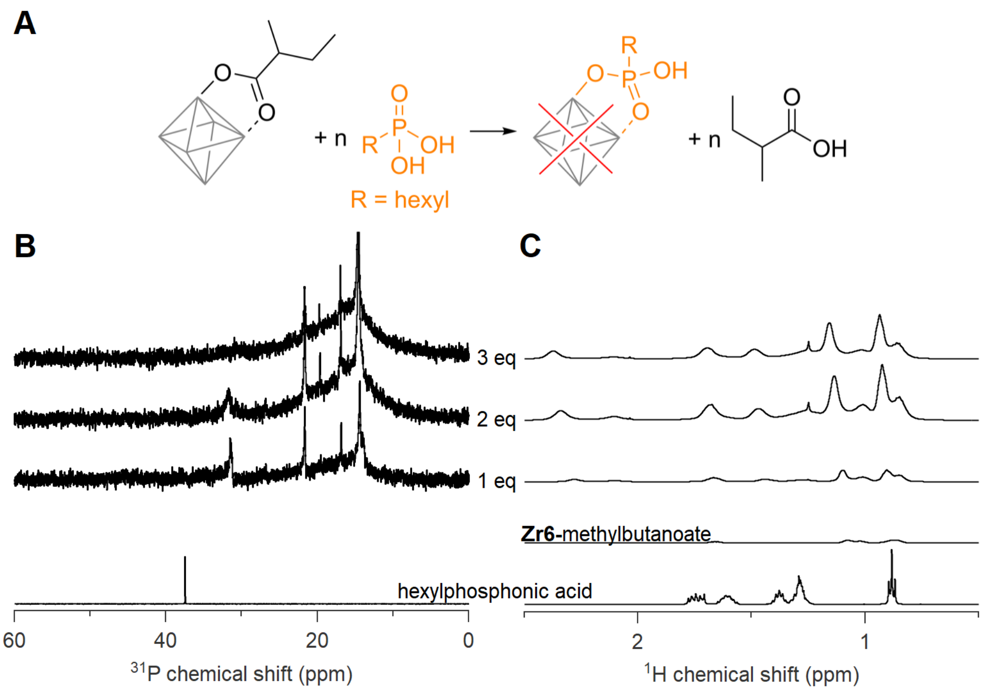

Figure S58: (A) Scheme for the titration of **Zr6**-methylbutanoate cluster with hexylphosphonic acid. ^{31}P (B) and ^{1}H (C) NMR of the titration of **Zr6**-methylbutanoate cluster with increasing equivalents of hexylphosphonic acid. The cluster concentration is 20 mg/mL in $CDCl_3$. Gelation prevented the data acquisition at high equivalents. ^{31}P and ^{1}H NMR of hexylphosphonic acid and methylbutanoate cluster are also provided (with one equivalent acetic acid added for the ^{31}P NMR reference).

4.2 Reference spectra

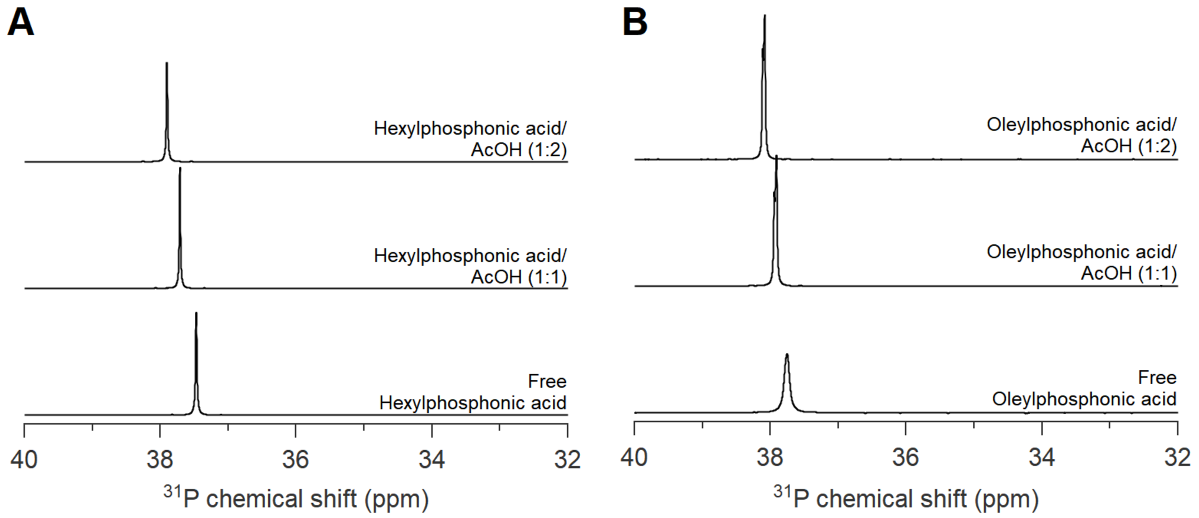

Figure S59: ^{31}P NMR of (A) hexylphosphonic acid and (B) oleylphosphonic acid with increasing equivalents of acetic acid. The more the acetic acid, the more deshielded the phosphorus signal.

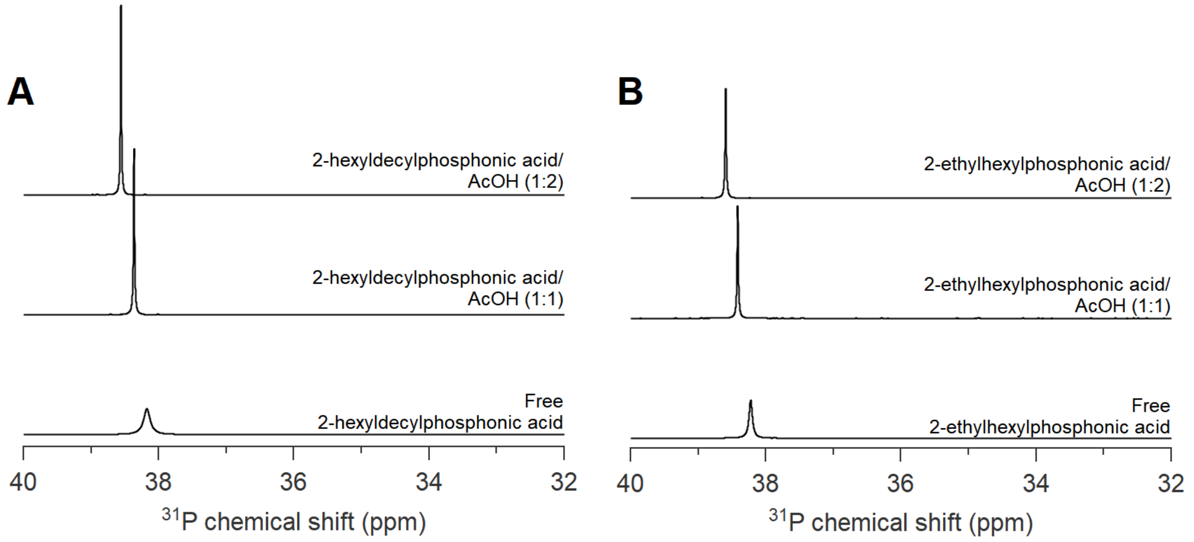

Figure S60: ^{31}P NMR of (A) 2-hexyldecylphosphonic acid and (B) 2-ethylhexylphosphonic acid with increasing equivalents of acetic acid. The more the acetic acid, the more deshielded the phosphorus signal.

4.3 ^{1}H and ^{31}P NMR spectra of purified clusters

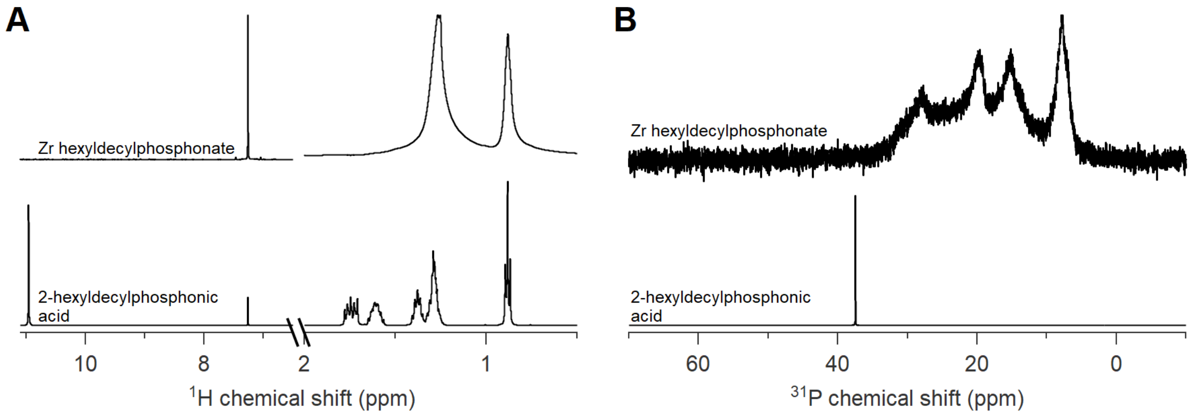

Figure S61: ^{1}H (A) and ^{31}P (B) NMR of purified ligand exchanged **Zr12**-acetate cluster with 2-hexyldecylphosphonic acid in $CDCl_3$. ^{1}H and ^{31}P NMR of free acids are provided as reference. The broadening of NMR signals confirms the cluster binding of new 2-hexyldecylphosphonate ligands.

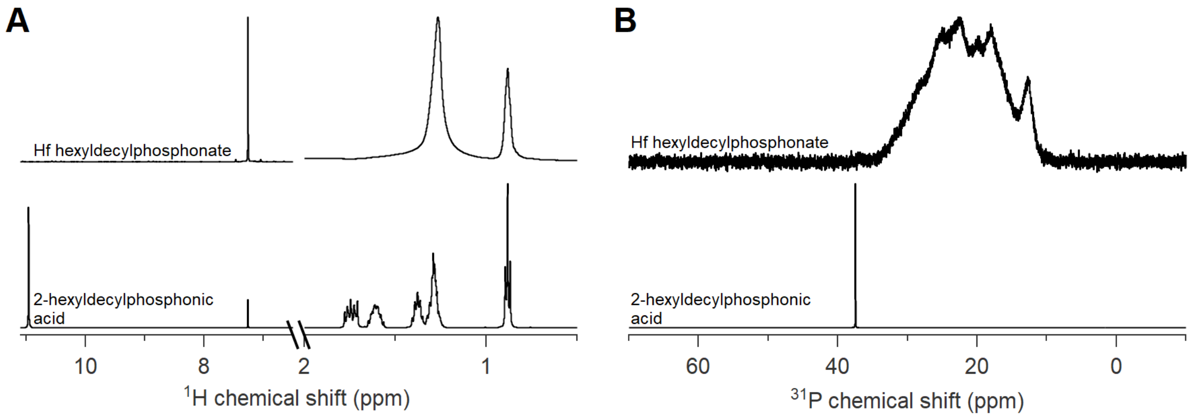

Figure S62: ^{1}H (A) and ^{31}P (B) NMR of purified ligand exchanged **Hf12**-acetate cluster with 2-hexyldecylphosphonic acid in $CDCl_3$. ^{1}H and ^{31}P NMR of free acids are provided as reference. The broadening of NMR signals confirms the cluster binding of new 2-hexyldecylphosphonate ligands.

4.4 PDF refinement data

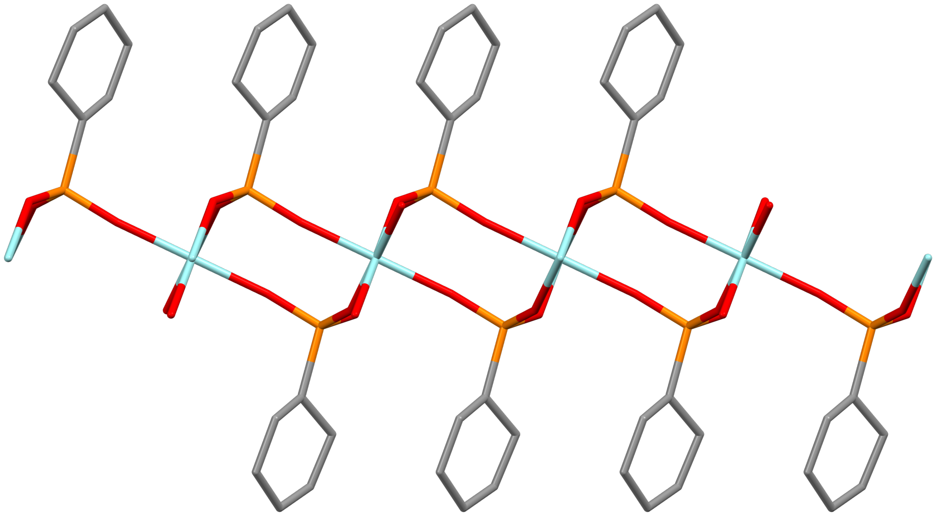

Figure S63: Crystal structure of layered zirconium phenylphosphonate (JPCDS:44-2000). Cyan atoms represent zirconium; all other atoms follow conventional CPK coloring. Hydrogen atoms are omitted for clarity. M, O, and P represent metal (zirconium), oxygen and phosphorus, respectively.

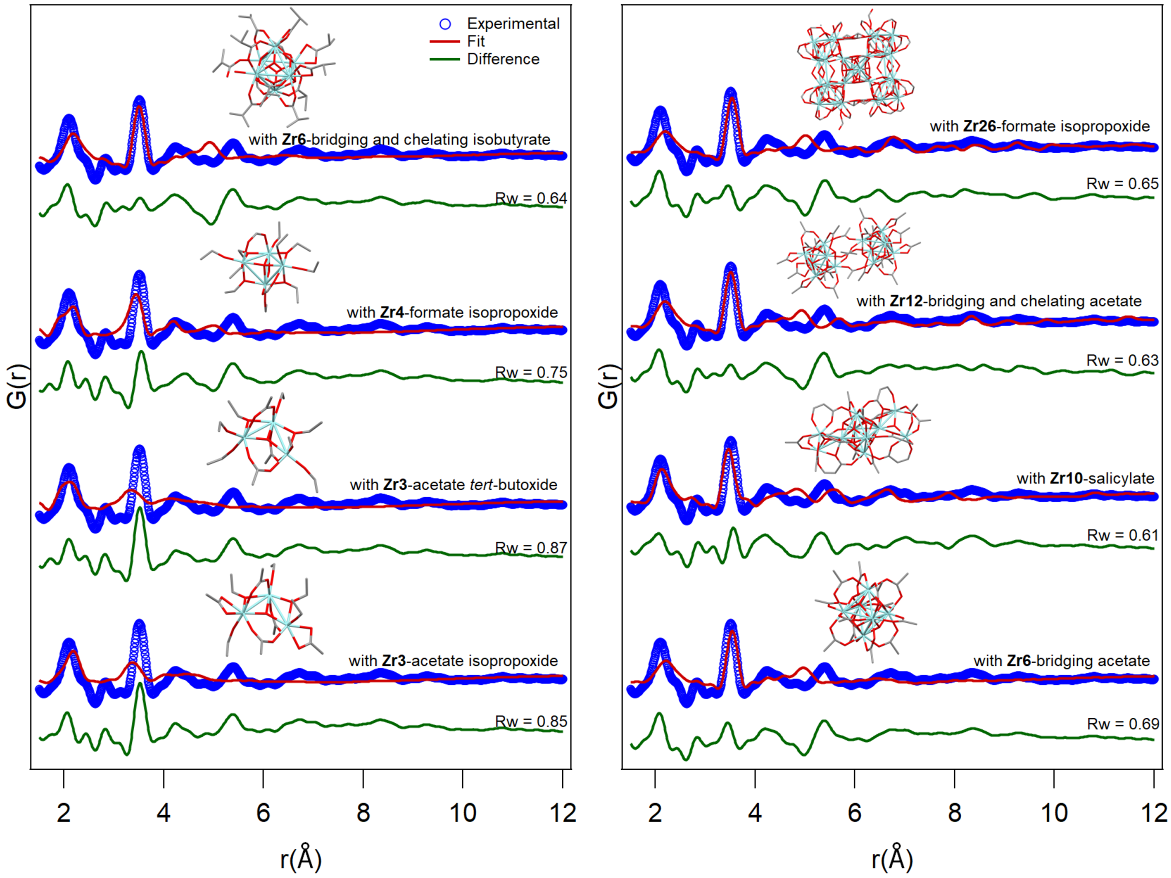

Figure S64: Single-phase PDF fit for hexyldecylphosphonate exchanged Zr clusters with various cluster structures reported in the literature. For each of the structural models, we removed the excess carbon atoms to arrive at a model with acetate ligands.

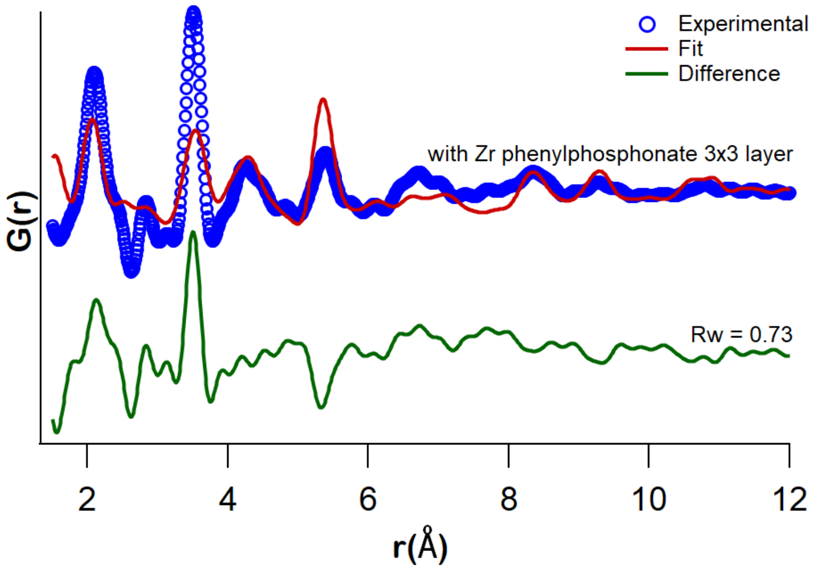

Figure S65: Single-phase PDF fit for hexyldecylphosphonate exchanged Zr clusters with a 3 x 3 layer of Zr phenylphosphonate (contains 9 zirconium atoms in total). The phenyl ring is removed from the structure model.

Table S5: Refined parameters after single-phase PDF fit for hexyldecylphosphonate exchanged Zr clusters.

**Model**	**Zr3**-acetate isopropoxide	**Zr3**-acetate *tert*-butoxide	**Zr4**-formate isopropoxide	**Zr6**-bridging chelating isobutyurate	**Zr6**-bridging acetate
**scale**	0.72	0.73	0.71	0.56	0.53
**Uiso Zr[Å^2]**	0.013	0.021	0.005	0.005	0.005
**Uiso O[Å^2]**	0.005	0.005	0.005	0.040	0.79
**Uiso C[Å^2]**	0.090	0.090	0.090	0.090	0.090
**delta2**	2.1	3.0	2.0	3.0	3.0
Rw	0.85	0.87	0.75	0.64	0.69
**Model**	**Zr10**-salicylate	**Zr12**-bridging chelating acetate	**Zr26**-formate isopropoxide	Zr phenylphosphonate 3 x 3 layer	
**scale**	0.65	0.58	0.52	0.93	
**Uiso Zr[Å^2]**	0.005	0.005	0.005	0.007	
**Uiso O[Å^2]**	0.011	0.055	0.057	0.017	
**Uiso P[Å^2]**	-	-	- 0.005		
**Uiso C[Å^2]**	0.090	0.090	0.090	0.085	
**delta2**	3.0	3.0	3.0	1.7	
Rw	0.61	0.63	0.65	0.73	

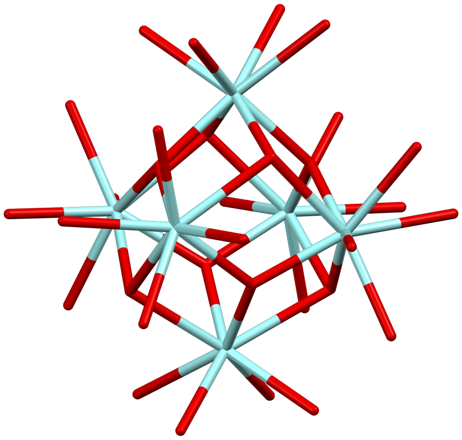

Figure S66: Structure model of **Zr6**-chelating bridging acetate cut from the crystal structure of **Zr12**-acetate.[S3] Carbon atoms are removed from structure model. Cyan atoms represent zirconium; all other atoms follow conventional CPK coloring.

Table S6: Refined parameters after dual-phase fitting phosphonate exchanged Hf clusters.

	Hf hexylphosphonate		Hf hexyldecylphosphonate	
**Model**	Phase I<br>Hf phosphonate<br>3 x 3 layer	Phase II<br>**Hf6**-acetate<br>chelating bridging	Phase I<br>Hf phosphonate<br>3 x 3 layer	Phase II<br>**Hf6**-acetate<br>chelating bridging
**scale**	3.43	2.75	1.88	2.58
**Uiso Hf[Å^2]**	0.006	0.005	0.006	0.006
**Uiso O[Å^2]**	0.010	0.025	0.010	0.026
**Uiso P[Å^2]**	0.007	-	0.009	-
**Uiso C[Å^2]**	0.056	-	0.100	-
**delta2**	3.50	3.44	3.50	3.50
Rw	0.23		0.25	
**Amplitude (A)**	-		1.45	
**wasyn**	-		5.56	
λ	-		6.30	
ϕ	-		-0.90	
θ	-		2.04	
**wsig**	-		0.81	

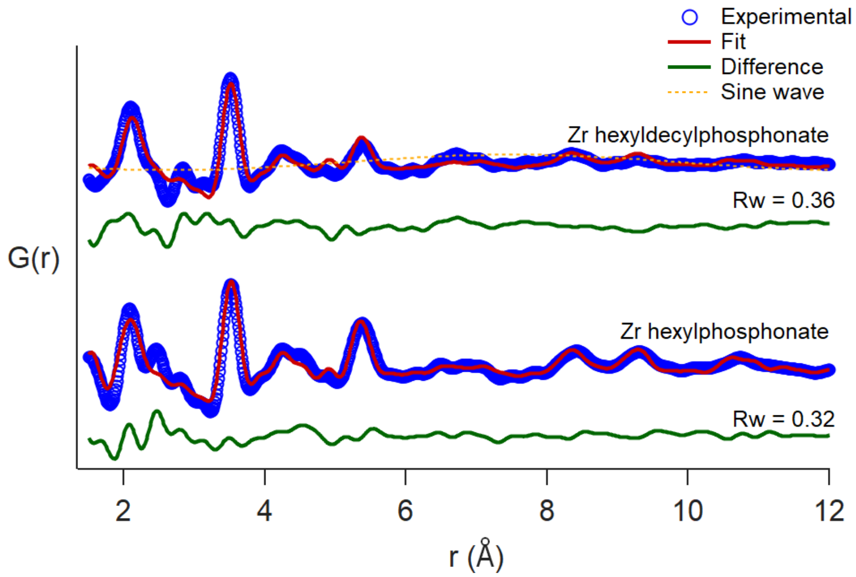

Figure S67: Dual phase PDF fit for phosphonate-exchanged Zr clusters with a 3 x 3 layer of Zr phenylphosphonate (which contains 9 zirconium atoms in total) and **Zr6**-chelating bridging acetate. Refined parameters are tabulated in Table S7.

Table S7: Refined parameters after dual-phase fitting phosphonate exchanged Zr clusters.

	Zr hexylphosphonate		Zr hexyldecylphosphonate	
**Model**	Phase I<br>Zr phosphonate<br>5 x 5 layer	Phase II<br>**Zr6**-acetate<br>chelating bridging	Phase I<br>Zr phosphonate<br>3 x 3 layer	Phase II<br>**Zr6**-acetate<br>chelating bridging
**scale**	0.079	0.044	0.69	0.56
**Uiso Zr[Å^2]**	0.006	0.003	0.006	0.004
**Uiso O[Å^2]**	0.020	0.043	0.010	0.032
**Uiso P[Å^2]**	0.010	-	0.009	-
**Uiso C[Å^2]**	0.09	-	0.040	-
**delta2**	2.5	2.5	1.0	2.5
Rw	0.32		0.36	
**Amplitude (A)**	-		0.60	
**wasyn**	-		-143.3	
λ	-		-9.7	
ϕ	-		-162.0	
θ	-		-62.2	
**wsig**	-		-37.9	

4.5 Dynamic light scattering analysis

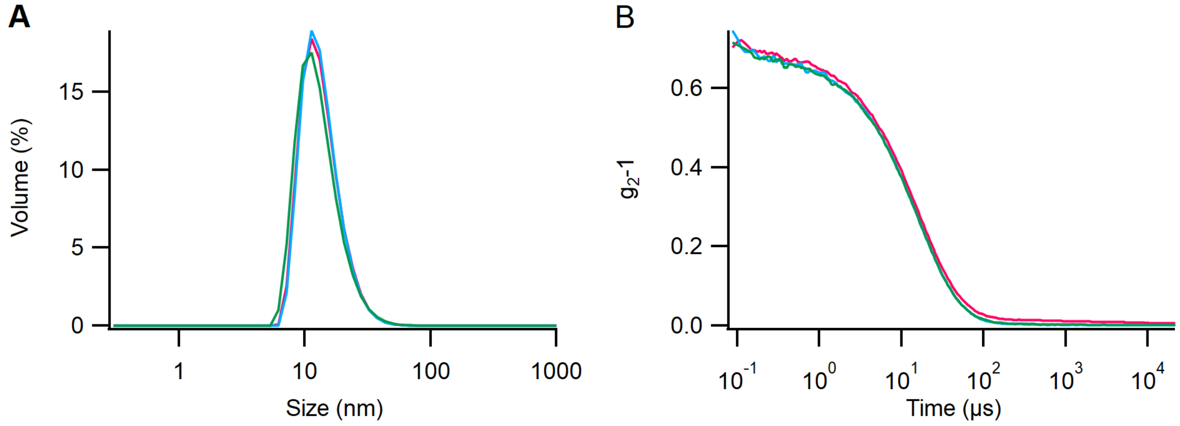

Figure S68: (A) DLS particle size distribution (by volume) and (B) correlogram for measurements of 10 mg/mL solution of zirconium hexyldecylphosphonate in chloroform. Z-average = 22.74 ± 2.13 nm, polydispersity index = 0.2251 ± 0.0403. The different colors represent individual measurements taken in triplicate.

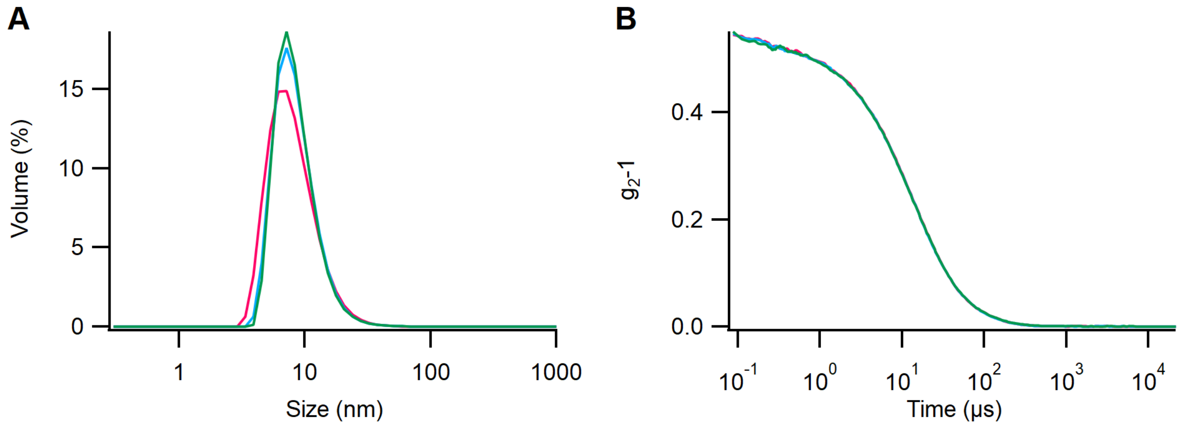

Figure S69: (A) DLS particle size distribution (by volume) and (B) correlogram for measurements of 10 mg/mL solution of hafnium hexyldecylphosphonate in chloroform. Z-average = 20.19 ± 0.05 nm, polydispersity index = 0.433 ± 0.0022. The different colors represent individual measurements taken in triplicate.

4.6 FT-IR spectra

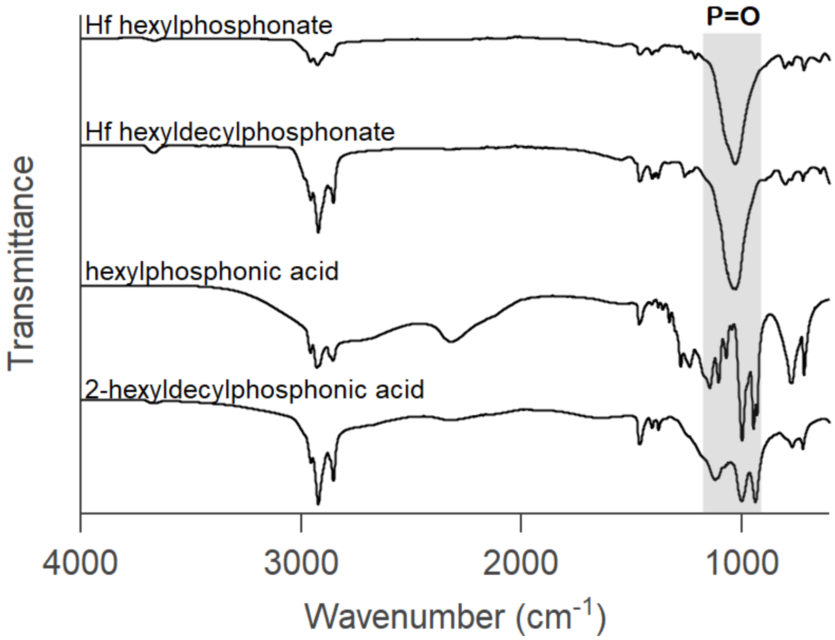

Figure S70: IR spectra of phosphonate exchanged hafnium clusters after isolation and purification. IR spectra of free acids are provided as reference.

References

(S1) Hennig, C.; Weiss, S.; Kraus, W.; Kretzschmar, J.; Scheinost, A. C. Solution Species and Crystal Structure of Zr(IV) Acetate. *Inorg. Chem.* **2017**, *56*, 2473–2480.

(S2) Kickelbick, G.; Schubert, U. Oxozirconium Methacrylate Clusters: $Zr_6(OH)_4O_4(OMc)_{12}$ and $Zr_4O_2(OMc)_{12}$ (OMc = Methacrylate). *Ber. Dtsch. Chem. Ges.* **1997**, *130*, 473–478.

(S3) Puchberger, M.; Kogler, F. R.; Jupa, M.; Gross, S.; Fric, H.; Kickelbick, G.; Schubert, U. Can the Clusters $Zr_6O_4(OH)_4(OOCR)_{12}$ and $[Zr_6O_4(OH)_4(OOCR)_{12}]_2$ Be Converted into Each Other? *Eur. J. Inorg. Chem.* **2006**, *2006*, 3283–3293.

(S4) Kickelbick, G.; Wiede, P.; Schubert, U. Variations in cap-

ping the $Zr_6O_4(OH)_4$ cluster core: X-ray structure analyses of $[Zr_6(OH)_4O_4(OOC–CH=CH_2)_{10}]_2(\mu–OOC–CH=CH_2)_4$ and $Zr_6(OH)_4O_4(OOCR)_{12}(PrOH)$ (R=Ph, CMe=CH_2). *Inorg. Chim. Acta* **1999**, *284*, 1–7.

(S5) Ji, Z.; Zhang, H.; Liu, H.; Yaghi, O. M.; Yang, P. Cytoprotective metal-organic frameworks for anaerobic bacteria. *Proc. Natl. Acad. Sci.* **2018**, *115*, 10582–10587.
